# Adjustments of the Phytochemical Profile of Broccoli to Low and High Growing Temperatures: Implications for the Bioactivity of Its Extracts

**DOI:** 10.3390/ijms25073677

**Published:** 2024-03-26

**Authors:** Ivana Šola, Daria Gmižić, Marija Pinterić, Ana Tot, Jutta Ludwig-Müller

**Affiliations:** 1 Department of Biology, Faculty of Science, University of Zagreb, Horvatovac 102a, 10000 Zagreb, Croatia; 2 Division of Molecular Medicine, Ruđer Bošković Institute, Bijenička 54, 10000 Zagreb, Croatia; 3 Andrija Štampar Teaching Institute of Public Health, Mirogojska 16, 10000 Zagreb, Croatia; 4 Faculty of Biology, Technische Universität Dresden, Zellescher Weg 20b, 01217 Dresden, Germany

**Keywords:** auxins, Brassicaceae, climate change, metabolic response, microgreens, photosynthetic pigments, polyphenolics, ROS, temperature stress, vitamin C

## Abstract

Climate change causes shifts in temperature patterns, and plants adapt their chemical content in order to survive. We compared the effect of low (LT) and high (HT) growing temperatures on the phytochemical content of broccoli (*Brassica oleracea* L. convar. *botrytis* (L.) Alef. var. *cymosa* Duch.) microgreens and the bioactivity of their extracts. Using different spectrophotometric, LC-MS/MS, GC-MS, and statistical methods, we found that LT increased the total phenolics and tannins in broccoli. The total glucosinolates were also increased by LT; however, they were decreased by HT. Soluble sugars, known osmoprotectants, were increased by both types of stress, considerably more by HT than LT, suggesting that HT causes a more intense osmotic imbalance. Both temperatures were detrimental for chlorophyll, with HT being more impactful than LT. HT increased hormone indole-3-acetic acid, implying an important role in broccoli’s defense. Ferulic and sinapic acid showed a trade-off scheme: HT increased ferulic while LT increased sinapic acid. Both stresses decreased the potential of broccoli to act against H_2_O_2_ damage in mouse embryonal fibroblasts (MEF), human keratinocytes, and liver cancer cells. Among the tested cell types treated by H_2_O_2_, the most significant reduction in ROS (36.61%) was recorded in MEF cells treated with RT extracts. The potential of broccoli extracts to inhibit α-amylase increased following both temperature stresses; however, the inhibition of pancreatic lipase was increased by LT only. From the perspective of nutritional value, and based on the obtained results, we conclude that LT conditions result in more nutritious broccoli microgreens than HT.

## 1. Introduction

Brassicaceae plants, commonly known as the mustard or cabbage family, are of great scientific interest due to their economic, agricultural, and nutritional significance. Aside from their very well-known in vitro activity, such as antioxidant, anti-inflammatory, antiproliferative, antimicrobial, and cytotoxic activity [1], they are also commonly included in preclinical trials on mice and rats [2], and now human clinical trials as well [3]. Their use has been extended such that even the seed meal produced after the extraction of oil from their seeds is being applied for the management of plant-parasitic nematodes [4]. Moreover, due to their strong antioxidant, antifungal, and antibacterial capacity, their extracts are used as food preservatives [5].

Microgreens have become increasingly popular in recent years, both in culinary circles and among health-conscious consumers. Unlike sprouts, which are harvested very early in the plant’s growth, often when the root and shoot are still intact and tightly packed together, microgreens are harvested slightly later in the growth cycle. The edible portion consists of the stem, cotyledons (seed leaves), and possibly the emerging first true leaves [6]. In this stage of growth, plants hyperaccumulate nutrients, often containing higher concentrations of vitamins, minerals, and antioxidants compared to their mature counterparts [7,8,9]. Among them, *Brassica oleracea* microgreens stand out for their rich content of various beneficial compounds [10,11]. They are especially rich in sulforaphane, a cancer chemoprotective compound [12], which is why they have been used in clinical settings among humans for more than 10 years [13,14].

A plant’s resistance in the environment is based on two main types of reaction, avoidance and tolerance [15]. The reaction depends on the type and intensity of the environmental challenge. One of the most evident challenges is variations in temperature, which can have a major impact on the physiological and biochemical processes in plants [16,17]. The regulation of metabolite biosynthesis in response to temperature stress is tightly controlled by complex signaling networks involving transcription factors, hormone signaling pathways (e.g., abscisic acid, jasmonic acid, salicylic acid), and environmental sensing mechanisms. The stress-responsive genes encoding the enzymes involved in metabolite biosynthesis are often transcriptionally activated or repressed in response to stress signals, leading to dynamic changes in metabolite profiles. The crosstalk between the different signaling pathways allows plants to integrate multiple stress signals and fine-tune their metabolic responses for optimal adaptation to changing environmental conditions [18]. Such metabolic adjustments may very well have a significant impact on the nutritional potential of plants and the biological activity of their extracts.

The predicted increase in global air temperature by 0.2 °C per decade is consistent with many climate change projections based on current trends and greenhouse gas emissions. If this trend continues, it could lead to temperatures that are 1.8–4.0 °C higher than the current levels by the year 2100 [16]. Plants can enhance their tolerance to heat stress by modulating the expression of the genes involved in osmoprotectant biosynthesis, detoxification, ion transport, and stress response regulation [19]. These multiple parallel effects consequently change plant–nutrient relations [20].

On the other hand, low-temperature stress contributes to the rigidification of biological membranes, which can compromise cellular integrity and function, ultimately resulting in cell death [21]. It disrupts the electron transport chains in chloroplasts and mitochondria, leading to the generation of reactive oxygen species (ROS). Reduced photosynthetic activity limits energy production and carbon assimilation, compromising plant growth and development. Among the genes responsive to low temperatures are those that code for soluble sugars and proline, xanthophylls and carotenoids, late embryogenesis abundant proteins, heat shock proteins, cold shock proteins, and dehydrins [22]. Aside from genetic factors, epigenetic factors also participate in a plant’s adjustment to temperature stress [23].

The purpose of this work was to assess the effect of low (LT) and high (HT) growing temperatures on the phytochemical profile of broccoli (*Brassica oleracea* L. convar. *botrytis* (L.) Alef. var. *cymosa* Duch.) microgreens, as well as the biological effects of its extracts. We aimed to determine the level of susceptibility/resistance of broccoli’s phytochemical parameters and its extracts’ bioactivity in response to temperature stress and single out sensitive ones as potential markers of LT/HT stress. Moreover, these data can contribute to the maximum utilization of the benefits of this plant material to maintain health and might be useful to the producers of microgreens in the sense of adjusting the temperature conditions in order to achieve the maximum concentration of a certain compound and/or the maximum antioxidant effect. For this purpose, we measured the impact of LT and HT on *(a)* different groups of phenolics; *(b)* total glucosinolates, proteins, and soluble sugars; *(c)* photosynthetic pigments; *(d)* plant hormones; *(e)* vitamin C; and *(f)* individual phenolic acids and flavonoids in broccoli microgreens. In addition, we screened for the possible effects of LT and HT on *(g)* antioxidant capacity, *(h)* the potential of extracts to change the in vitro activity of enzymes α-amylase and lipase, and *(i*) their potential to change the levels of reactive oxygen species (ROS) in different types of cells. The collected data were *(j)* statistically processed using one-way analysis of variance (ANOVA) and Student’s *t*-test to assess the significance of differences between the samples, Pearson’s correlations for the analysis of the relationships between the measured parameters, and principal component analysis (PCA) and hierarchical clustering in order to visualize the proximity/distance of the samples. The results revealed that two of the most significant changes were under LT: soluble sugars increased by 137%, whereas total anthocyanins decreased by 81%. The third most significant change was under the HT effect—an IAA increase of 78%. Based on all of the collected data, we concluded that both HT and LT significantly changed the broccoli microgreens at the level of the parameters analyzed, by 82% and 81%, respectively. Among the altered parameters, HT increased 44% of them, while LT increased 55%. The parameters that were significantly changed by HT, but not LT, were the total proanthocyanidins, kaempferol, sinapic acid, the hormone IAA, the antioxidant capacity measured by the ABTS method, and the concentration of ROS in HepG2 cells treated with H_2_O_2_ and broccoli extract at 0.05 mg/mL. On the other hand, the parameters significantly affected by LT, but not HT, were the total proteins and tannins, the antioxidant capacity measured by the DPPH and FRAP methods, and the potential to inhibit the enzyme lipase. We assumed that, among these parameters responsive to one but not to the other temperature stress type, we could search for the mediators that are crucial for plants’ adjustment to HT/LT stress.

## 2. Results and Discussion

Different temperature ranges can lead to distinct changes in the morphology, physiology, and biochemistry of plants. Several components function as potential cold sensors: membranes, calcium channels, and G-protein regulators encoded by the rice chilling tolerance divergence 1 (COLD1) gene [24]. HT stress also causes changes in plasma membrane fluidity and can induce the formation of non-functional proteins and an increase in reactive oxygen species (ROS). The regulatory network activated by the transduction of the temperature stress signal initiates multiple responses within the plant, including adjustments in metabolic pathways and the activation of specific stress-related mechanisms. Understanding these temperature-dependent processes is essential in addressing the impacts of climate change on plant ecosystems and developing strategies to enhance the resilience of plants in the face of environmental fluctuations. Within the scope of this work, we compared the effects of HT and LT on the phytochemical responses of broccoli microgreens and consequently the biological effects of their extracts.

### 2.1. Effect of High and Low Growing Temperatures on Polyphenolics in Broccoli Microgreens

One of the stress-responsive metabolic pathways adopted by plants to cope with unfavorable conditions is that of polyphenolic compounds. Polyphenolics have strong antioxidant capacities, which are essential in scavenging reactive oxygen species (ROS) generated during abiotic stress [25]. By adjusting the levels of polyphenolics, plants can enhance their resilience, protect cellular components, and optimize resource utilization to cope with challenging temperature conditions. In our work, we screened for the effects of HT and LT on the major groups of polyphenolics with the aim of distinguishing susceptible from resistant ones. As shown in Table 1, the highest level of change, both under HT and LT effects, was recorded for total anthocyanins, a decrease of 33% under HT and 81% under LT conditions. On the other hand, no group of polyphenolics showed resistance toward HT, while, under LT, the most resistant were the total flavonoids and phenolic acids. Interestingly, all of the measured groups of polyphenolics showed a negative change under the effect of HT; however, LT affected some of the groups positively and others negatively. We observed a positive effect of LT (15% increase) on total phenolics. The same result was recorded in turnip tops [26]. Further, total tannins were significantly increased under the effect of LT (increase by 24%); however, total proanthocyanidins (condensed tannins) were significantly affected by HT and, conversely, decreased. This shows that temperature stress affected both the amount and composition of tannins in the broccoli microgreens. The effect of HT on total proanthocyanidins was investigated in the grains of different sorghum (*Sorghum bicolor*) genotypes and the result was the same, with a decrease [27]. Although the effect of warm temperatures on the content of total tannins in *Quercus rubra* leaves [28] was different than in our study on broccoli microgreens, the same conclusion was drawn regarding the effect of temperature stress on the content and composition of tannins. Furthermore, since the content of total tannins in the broccoli microgreens under the effect of LT significantly increased, and the condensed tannins did not change, we assumed that another sub-group of tannins, hydrolysable tannins, was increased. The same result regarding total proanthocyanidins under the effect of HT and LT as in our study was recorded in buckwheat (*Fagopyrum esculentum* Moench) seeds [29].

### 2.2. Effects of High and Low Growing Temperatures on Total Glucosinolates, Proteins, and Sugars in Broccoli Microgreens

The level of glucosinolates in a plant is influenced by the genotype, developmental stage, and environmental factors [30]. For example, when the tissues of plants from the Brassicales order are mechanically damaged, the enzyme myrosinase comes into contact with glucosinolates and hydrolyzes them into volatile compounds. This process is part of the plant’s defense mechanism against herbivores and pathogens [31]. A similar mechanism takes place when plants are grown under temperature stress. More specifically, the membrane structure can be damaged, myrosinase and glucosinolates may react, and toxic products may be released. In addition, different temperatures during growth can affect the expression of the genes involved in the synthesis of glucosinolates [32]. In our study, the total glucosinolates in broccoli microgreens grown under HT were significantly decreased, while being increased under LT (Table 2). We assumed that the decrease under HT was due to the increased activity of myrosinase with higher daily temperatures [33]. The same result was recorded in our previous work [34] on broccoli seedlings.

The total glucosinolates in kale (*Brassica oleracea* var. *alboglabra* Bailey) were decreased under HT [35]. In rocket salad grown under high summer temperatures, specific glucosinolates were decreased [36]. In curly kale (*Brassica oleracea* L. var *acephala*), the total glucosinolates decreased under HT; however, in this study, we used 21/15 °C (day/night) as the high temperature and 15/9 °C as low [37]. This might suggest that in different temperature range combinations, the higher one will result in lower amounts of glucosinolates. The glucosinolate concentration in field-grown rutabaga (*Brassica napus* spp. *rapifera*) roots without peel was higher in the group stored for two weeks at 0 °C than in that stored at 10 °C [38]. However, in rutabaga grown at higher (21 °C) and lower (9 °C) temperatures and analyzed immediately after collection, without storage, more glucosinolates were present in the roots grown at 21 °C [30]. In young broccoli plants treated with cold water, the total glucosinolates were increased, while treatment with warm water decreased them [11]. Short-term HT (40 °C for 8 h) increased the total glucosinolates in pakchoi seedlings (*Brassica rapa*) [39]. The content of glucosinolates in broccoli sprouts cultivated at 30/15 °C (day/night) was higher than in those grown at lower temperatures (22/15 and 18/12 °C) [40].

During temperature stress, proteins undergo many post-translational modifications, which ultimately directs their function, localization, and interactions with other molecules and their stability [18]. The total proteins significantly differed between the LT (53.87 ± 3.02 mg bovine serum albumine equivalent (BSAE/g dm)) and HT (49.10 ± 3.39 mg BSAE/g dm) groups of broccoli microgreens, while the amount in the control group (51.35 ± 2.54 mg BSAE/g dm) was between these two, i.e., it did not differ from the test groups. In our previous work with a longer HT period, the decrease in proteins was significant [34]. This indicated that the proteins in broccoli microgreens are able to resist HT for a shorter period of time (5 days), but that a longer period (14 days) is detrimental. One of the possible reasons behind this is the fact that HT decreases the rate of protein synthesis due to the non-availability of active mRNA [41]. Additionally, in terms of post-translational modifications to proteins, as an adjustment to stress, it might be that the degradation of proteins decreased their concentration [42]. In mungbean (*Vigna radiata*) sown at HT, the total seed protein content was decreased compared to plants sown at a regular temperature [43]. Regarding the LT effect, we observed a tendency toward an increase (positive change of 5%). For comparison, using two-dimensional gel electrophoresis and MALDI-TOF-TOF mass spectroscopy, significant increases in the relative abundance of antioxidant-related proteins were recorded in LT-tolerant winter wheat cultivars [44]. Moreover, a significant number of genes that encode signal transduction and regulatory proteins, like mitogen-activated protein (MAP) kinase, MAP kinase kinase kinase, calmodulin-related proteins, and 14-3-3 proteins, are induced during cold acclimation [45]. Since temperature acclimation requires energy, we hypothesize that the proteins involved in carbohydrate catabolism (glycolysis enzymes) [46] might also be increased.

Soluble sugars play a crucial role in protecting plant cells from the damage caused by temperature stress. They act as osmoprotectants and nutrients, interact with the lipid bilayer, and exhibit hormone-like activities in signal transduction pathways [47]. We recorded a significant increase in soluble sugars in broccoli microgreens after both HT and LT stress. Moreover, HT increased the sugars significantly more than LT, with an increase of 137%, compared to 73% in the LT group. This suggests that, in broccoli microgreens, the formation of sugars is much greater under the influence of HT than LT conditions. We observed a similar effect after the treatment of young broccoli plants with hot and cold water [11], with the exception that, in this developmental stage, cold water even reduced the amount of soluble sugars. In comparison, the soluble sugars were significantly increased by LT in seven out of fourteen tested broccoli (*Brassica oleracea* L. var. *italica*) cultivars [48]. In six cultivars, it showed a tendency to increase, but not significantly (*p* ≤ 0.05), while it decreased significantly in only one cultivar [48]. In both the young and mature leaves of winter oilseed rape (*B. napus* L. ssp. *oleifera*) cultivars, the Eurol and Hansen soluble sugars were increased under LT stress [49]. We assume that one of these sugars was sucrose, as in the case of potato (*Solanum tuberosum* L.) leaves [50]. Moreover, the exogenous application of soluble sugars has been shown to enhance freezing tolerance in various higher plant species [51]. This phenomenon highlights the role of sugars not only as endogenous signaling and protective molecules but also as external factors that can confer stress tolerance.

Very recently, Hahn et al. [52] suggested that the large amount of glucose available as a result of LT could be used to build more glucosinolates. If LT leads to changes in carbohydrate metabolism, there might be increased availability of glucose for the biosynthesis of glucosinolates. Describing the increase in glucosinolates as an unintentional side effect implies that the plant, in response to LT, inadvertently produces more of these specialized metabolites. This suggests that the plant may not necessarily intend to produce more glucosinolates but does so as a consequence of metabolic changes.

### 2.3. Effects of High and Low Growing Temperatures on Photosynthetic Pigments in Broccoli Microgreens

Photosynthesis, a fundamental mechanism primarily facilitated by photosynthetic pigments, by which plants convert light into chemical energy [53], is able to adjust to the growing temperature [54]. It is often the first cellular mechanism that is inhibited by extreme temperatures [55]. Understanding how photosynthetic pigments react to various temperature regimes is crucial in clarifying the adaptation mechanisms that plants use to deal with temperature stress. In our study, a significant reduction in the concentrations of both chlorophyll *a* (HT −3%, LT −3%) and chlorophyll *b* (HT −23%, LT −15%), as well as in the overall content of chlorophyll (HT –12%, LT −8%), under HT and LT conditions compared to the RT was observed (Table 3). Under both types of temperature stress, chlorophyll *b* was more affected than chlorophyll *a*, indicating that the aldehyde group in chlorophyll *a* might contribute to the resistance toward temperature stress. This is consistent with previous research on the subject and may also be attributed to the fact that chlorophyll *b* undergoes faster degradation, ultimately transforming into chlorophyll *a* as part of the breakdown process [56,57]. A reduction in total chlorophyll content under temperature stress has already been observed in other plant species, e.g., broccoli [34], cabbage [56], kale [56,58], *Thalassia hemprichii* [59], watermelon [60], and squash [61]. These results indicate the sensitivity of chlorophyll *a* and *b* to temperature, whether increased or decreased. We hypothesize that the reduction in chlorophyll levels under HT and LT is probably caused by damage to the photosynthetic apparatus, disturbances in chlorophyll biosynthesis, accelerated degradation, or a synergistic interplay between these processes [62,63].

Since the baseline Chl *a*/*b* is an indicator of a functional photosynthetic system, any significant changes suggest adjustments to mitigate the effects of stress on the photosynthetic apparatus. The ratio of Chl *a* and Chl *b* was increased after both types of temperature stress (HT 24%, LT 14%), but it is important to note that it was significantly greater under the influence of HT than under LT. Based on this result, we hypothesized that the broccoli microgreens’ chlorophyll system is more susceptible to elevated than to lower temperatures. More precisely, chlorophyll *b* was the one that was significantly more affected by HT than by LT. The same result regarding HT was recorded in three genotypes of the *Cucurbita* species [61]. This is not consistent with studies on cabbage and kale [56] and paprika [64], which showed the higher susceptibility of chlorophyll *b* to LT than to HT, emphasizing the variety- and species-specific response of the photosynthetic apparatus to different temperatures.

Contrary to chlorophyll, the carotenoid and non-enzymatic antioxidant content increased under both HT (17%) and LT (14%). The same result was recorded previously in broccoli seedlings collected after a longer period of HT [34] stress than in the current experiment. For comparison, in sweet osmanthus (*Osmanthus fragrans* Lour.), HT and LT had an opposite effect on total carotenoids; HT decreased while LT increased the total carotenoids. Under HT, lutein and *β*-carotene increased in kale (*B. oleracea* L.), while, in spinach (*Spinacia oleracea* L.), they decreased [65]. In both young and old leaves of *Eucalyptus parramattensis*, the total carotenoid content increased with temperature; however, data on the statistical significance were not presented [66]. In contrast, in the young and mature leaves of winter oilseed rape (*B. napus* L. ssp. *oleifera*) cultivars, the Eurol and Hansen carotenoids decreased after LT stress [49]. Three cultivars of tomato (*Lycopersicon esculentum*) showed different responses to HT at the level of total carotenoids [67], as well as three genotypes of *Cucurbita* species [61], which indicated a high degree of response specificity.

The two test groups and the control group of broccoli that we analyzed significantly differed from each other in the sum of total chlorophyll and carotenoids, as well as in the share of total chlorophyll or carotenoids. The sum of total chlorophyll and carotenoids was the highest in the control group and the lowest in the HT group, and the same was observed for the share of total chlorophyll. The opposite was noted for the share of carotenoids: its sum was the highest in the HT group and the lowest in the control group. We hypothesize that carotenoids play an important role in the acclimation of broccoli microgreens to HT.

The generation of reactive oxygen species (ROS) in plants is a common response to various environmental stresses. The equilibrium between the formation and detoxification of ROS is a prerequisite for normal cell functioning. The precursors of porphyrins generate radicals and ROS [68]. Therefore, in the context of environmental stress, plants will tend to decrease the amount of these ROS-generating compounds. In our study, the total porphyrins were significantly decreased by both HT and LT stress, and no difference was observed between HT and LT. The same result was recorded for rice (*Oryza sativa* L.) as well [69].

### 2.4. Effects of High and Low Growing Temperatures on IAA and ABA Concentrations in Broccoli Microgreens

Auxins, a class of plant hormones, play a central role in regulating various aspects of plant growth and development, both in control and stress conditions [70]. Temperature can influence the rate of plant growth, and there is evidence to suggest that temperature modulation correlates with changes in the levels and activity of auxins [71]. Moreover, in *Arabidopsis* hypocotyl, the response to HT correlated with increased auxin production, which in turn altered the cellular homeostasis of auxin [72]. However, the relationship between temperature and auxin-mediated growth is complex and can vary depending on the plant species and specific environmental conditions. In our case, HT significantly increased the concentration of indole-3-acetic acid (IAA), causing a change of 78% (Table 4). On the other hand, LT showed a tendency to decrease it. However, at the level of *p* ≤ 0.05, this was not significant. For comparison, in *Arabidopsis* roots, the level of IAA was also increased after HT stress [70].

The level of abscisic acid (ABA) was not significantly affected by HT or LT, although it should be mentioned that we recorded a high standard deviation in the control group. Therefore, although HT led to an increase in ABA in the broccoli microgreens by 24%, due to the high standard deviation in the control group, this result was not significant. For comparison, in *Arabisopsis* seeds [73] and rice seeds [74], ABA was induced after HT stress. In *Zanthoxylum bungeanum* at the late stage of LT stress [75], and in two wheat genotypes [76], ABA was increased. Based on the level of change, we conclude that in broccoli microgreens, IAA is more affected by temperature stress than ABA. Moreover, both hormones were increased by HT—IAA was significantly affected, and ABA showed a tendency to increase.

### 2.5. Effects of High and Low Growing Temperatures on L-Ascorbic Acid, Individual Flavonoids, and Phenolic Acids in Broccoli Microgreens

During the process of photosynthesis, ROS can be generated, and vitamin C, also known as ascorbic acid, helps to neutralize these harmful molecules, preventing oxidative stress. In addition to this activity, it also acts as a cofactor for several enzymes involved in various metabolic processes in both plants and humans [77]. It is involved in various cellular processes, such as flowering time regulation, developmental senescence, programmed cell death (apoptosis), and responses to environmental challenges. This multifunctionality is likely mediated through complex signal transduction networks [78]. The significance of vitamin C for plant organisms is especially emphasized during stress. In such situations, vitamin C interacts with hormone signaling and contributes to the plant’s defense [79]. In this study, we analyzed the effects of LT and HT on the concentrations of free vitamin C and phenolics, i.e., before hydrolysis, and the concentrations of their derivatized forms, i.e., after hydrolysis. Our first observation was that *L*-ascorbic acid, the phenolic compounds sinapic and ferulic acid, and the flavonoids quercetin and kaempferol were present in the broccoli microgreens at significantly higher concentrations in conjugated than in free form (Table 5). This was expected since conjugation allows for the storage and inactivation of these highly bioactive molecules.

Both HT and LT significantly changed the free and conjugated *L*-ascorbic acid concentrations in the broccoli microgreens. HT significantly increased the concentration of free-form *L*-ascorbic acid, and LT decreased it (Table 5, Supplementary Figure S1). On the contrary, conjugated *L*-ascorbic acid was decreased in the HT group and increased in the LT group. This suggests a trade-off scheme among the free and conjugated form/s of *L*-ascorbic acid and their involvement in the acclimation of broccoli microgreens to HT/LT. In our previous work with broccoli seedlings treated by HT for a longer period of time (14 days) than in this study, we recorded no significant change in the conjugated *L*-ascorbic acid concentration [34]. This suggests a developmentally dependent vitamin C response of broccoli seedlings to HT. For comparison, vitamin C, measured as the sum of ascorbic and dehydroascorbic acid, was increased at the beginning of tomato fruit development (flowering and fruit set); then, no change was observed at a fruit diameter of around 40–50 mm; and, finally, it decreased in the fruit with a diameter of around 60 mm and in green, fully developed fruits [80]. In kiwifruit vines (*Actinidia deliciosa* var. *deliciosa* ‘Hayward’), vitamin C was also decreased after HT treatment [81]. Regarding LT treatment, in sweet corn (*Zea mays* L.) seedlings, LT decreased the concentration of ascorbic acid, while HT increased it [82]. However, these authors set up their experiment differently from ours, applying stress for a shorter period of time, i.e., for 0 h, 15 h, and 30 h.

Hydroxycinnamic acids ferulic and sinapic reacted oppositely to HT and LT. Ferulic acid, free and conjugated, was susceptible to both; HT significantly increased it, while LT decreased it (Table 5, Supplementary Figure S1). HT also showed a significantly stronger effect than LT, which altogether might indicate an important role of ferulic acid in the adjustment of broccoli to elevated environmental temperatures. This presumption is supported by results for blueberry (*Vaccinium corymbosum*) seedlings [83], where it was shown that pretreatment with exogenous ferulic acid alleviated the heat stress symptoms in the blueberries. HT significantly reduced the concentrations of both free and conjugated sinapic acid (Table 5, Supplementary Figure S1). On the other hand, under LT conditions, free sinapic acid was not affected significantly, but its conjugated form/s was/were increased. This suggests an inverse relation between ferulic and sinapic acid biosynthesis in broccoli microgreens under temperature stress. We assume that ferulic acid has a significant role in the protection of broccoli microgreens from HT, while sinapic acid serves for protection against LT. Depending on the type of temperature stress, broccoli microgreens will direct the biosynthetic pathway either toward ferulic or sinapic acid. This trade-off scheme was also recorded in young plants of broccoli treated with hot water [11]. This result also suggests that ferulic and sinapic acid might help to mitigate the effect of temperature stress in broccoli microgreens, if exogenously applied. Such an experiment has already been conducted on heat-stressed blueberry (*Vaccinium corymbosum*) seedlings, with very promising results [83].

The regulation of antioxidant systems during stress is a dynamic and intricate process. While the activity of antioxidant enzymes may decline during certain phases of stress progression, plants often have alternative defense mechanisms, including the involvement of specialized metabolites like flavonoids, to cope with oxidative stress and maintain cellular homeostasis [84,85]. The interplay between these different components contributes to the plant’s overall ability to adapt and survive under challenging environmental conditions. Flavonols constitute one of the most important sub-groups among flavonoids, and they are widely recognized for their diverse biological activity and health benefits [86]. Due to a catechol group in the B-ring, flavonols serve as the most effective antioxidants and crucial intermediates in a plant’s response to environmental challenges [87]. Even at low concentrations, they appear to play a crucial role in scavenging ROS and maintaining a balance, contributing to cellular health and the stress response [86]. Their unique chemical structure allows them to interact with and modulate the activity of the proteins involved in signaling pathways, which enables them to act as developmental regulators [88]. Flavonoids are known to be involved in temperature–plant interactions [89]. As discussed earlier, high temperatures generally decrease the concentrations of flavonoids, while low temperatures increase them, in the presence of light. In the broccoli microgreens, the flavonoid quercetin was more affected by LT than HT (Table 5, Supplementary Figure S1), and LT significantly increased its free form. One of the possible reasons might be the involvement of quercetin in the stabilization of membranes and/or proteins during cold stress [90,91]. Kaempferol was decreased by both temperature stresses (Table 5, Supplementary Figure S1), the most significant decrease being recorded in the group grown under HT; free kaempferol was decreased by as much as 77%. If we look at the quercetin:kaempferol ratio, under LT conditions, it increased, and the same has already been observed in eight kale (*B. oleracea*) cultivars growing in cool temperatures [92]. This result is consistent with the fact that low temperatures in general support the biosynthesis of flavonoids containing more hydroxyl groups [89]. Another conclusion, based on these results, is that, in broccoli microgreens, kaempferol is more susceptible to high than to low temperatures. The same result was recorded when we tested the effects of hot and cold water treatment on young broccoli plants [11].

### 2.6. Effects of High and Low Growing Temperatures on Antioxidant Capacity of Broccoli Microgreen Extracts

High and low temperatures can induce oxidative stress in plants, leading to the accumulation of ROS, which can damage cellular components like proteins, lipids, and DNA and further cause cellular dysfunction and ultimately cell death. Plants grown under temperature stress often exhibit increased antioxidant enzyme activity and the production of specialized metabolites with antioxidant properties to mitigate oxidative damage. The antioxidant capacity was measured by three methods (Table 6). According to the ABTS method, HT decreased the antioxidant potential of the extracts, while, according to the DPPH and FRAP methods, LT increased it.

### 2.7. Effects of High and Low Growing Temperatures on Potential of Broccoli Microgreen Extracts to Inhibit Enzymes α-Amylase and Lipase

The enzymes α-amylase and lipase play crucial roles in the breakdown and absorption of carbohydrates and fats, respectively, in the digestive system. By inhibiting them, the digestion and absorption of these nutrients are slowed down, leading to reduced postprandial glucose spikes and the decreased absorption of dietary fats. This is especially important for people suffering from diabetes type 2, obesity, and lipid peroxidation [93]. Research into plant foods with inhibitory effects on these enzymes is ongoing, with the aim of developing effective interventions for these chronic health conditions [94,95]. Although there are a plethora of data showing the potential of plants to inhibit the activity of these enzymes [96,97,98,99], we were not able to find any dealing with the effect of environmental factors on the ability of plants to inhibit the activity of digestive enzymes, except for our recent one [11]. This comes as a surprise since it is known that temperature stress induces metabolic rearrangements [100], which means that it might change the quality of a plant as food. Since HT and LT significantly changed some of the measured phytochemical parameters in the broccoli microgreens, we further tested whether they might also affect the potential of their extracts to inhibit the activity of the digestive enzymes α-amylase and lipase. As shown in Table 7, the extracts of HT- and LT-grown broccoli microgreens exhibited the inhibition of α-amylase at the level of 7.92 ± 2.40% and 7.68 ± 3.81%, respectively. Although this is a low percentage, and considering that RT-grown microgreens showed no inhibition at all, it shows that the HT and LT growth conditions did improve the potential of the broccoli microgreen extract to inhibit α-amylase. Compared to the activity against α-amylase, the activity of the broccoli microgreen extracts against lipase was higher. We had previously already found such a result when we analyzed the effects of hot and cold water stress on broccoli [11] and interspecific metabolite transfer for the biofortification of broccoli [101]. HT did not change the potential of the broccoli microgreens to inhibit lipase activity; however, LT significantly improved it.

### 2.8. Effects of Extracts of Broccoli Grown at Low and High Temperatures on Intracellular Levels of Reactive Oxygen Species

**Figure 1:**
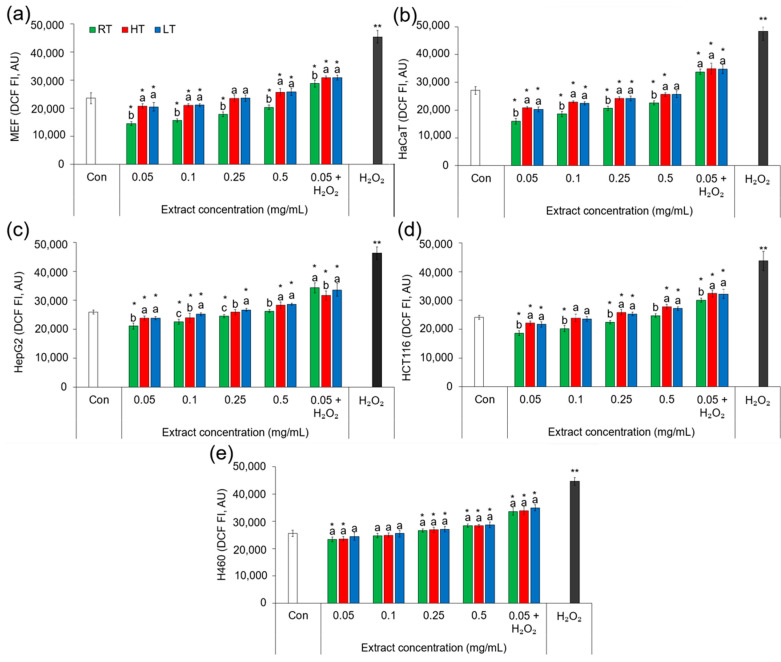
The impact of different concentrations of extracts from broccoli cultivated under room (RT), high (HT), and low (LT) temperature on the levels of ROS within (**a**) mouse embryonal fibroblasts (MEF), (**b**) normal human keratinocytes (HaCaT), (**c**) hepatocellular carcinoma (HepG2), (**d**) colorectal carcinoma (HCT116), and (**e**) lung carcinoma (H460) cell cultures. Values represent mean ± standard deviation of three biological replicates. Different letters indicate a significant difference between the RT, HT, and LT broccoli microgreens, separately for each extract concentration (ANOVA, Duncan test, *p* ≤ 0.05). An asterisk (*) indicates a significant difference between each group of cells treated with broccoli microgreen extracts and control cells (Student’s *t*-test, *p* ≤ 0.05). A double asterisk (**) indicates a significant difference between cells treated with H_2_O_2_ only and cells treated simultaneously with H_2_O_2_ and broccoli extract at a 0.05 mg/mL concentration (Student’s *t*-test, *p* ≤ 0.05). AU = arbitrary units; Con = control cells; DCF = dichlorodihydrofluorescein; FI = fluorescence intensity.

Extracts of broccoli contain a diverse array of phytochemicals, including antioxidants such as phenolics, flavonoids, and vitamins (Table 1 and Table 6), which can scavenge ROS and alleviate oxidative stress in cells. The composition and concentration of antioxidants in extracts can vary depending on the plant growth conditions. Studies have shown that plant extracts rich in antioxidants can effectively reduce the intracellular ROS levels and protect cells from oxidative damage induced by environmental stresses, including temperature extremes [102]. To investigate whether—and, if so, how—the cultivation of broccoli plants under the influence of high and low environmental temperatures affects the potential of their extracts to change the levels of intracellular ROS, we employed a fluorescence technique on five types of cell cultures (Figure 1) and tested four different concentrations of extracts (0.05–0.50 mg/mL). In each of the five cell types, the RT broccoli extract at the concentrations of 0.05 mg/mL and 0.25 mg/mL significantly reduced the concentration of ROS compared to the concentration in the control cells not treated with the extract. This shows that broccoli microgreens grown under room temperature have strong potential to protect cells from ROS. Interestingly, the most significant effect was achieved with the lowest concentration of the extract (0.05 mg/mL). The extracts of LT and HT broccoli were effective at the lowest concentration (0.05 mg/mL) only, and less successful than that of the RT broccoli. This suggests that under the influence of low and high growing temperatures, the potential of broccoli microgreens to reduce the intracellular levels of ROS significantly decreases. The most significant decrease (41%) in ROS under the influence of the RT broccoli extract was in human keratinocytes (HaCaT), which suggests that broccoli microgreen extracts might be useful in the protection of human skin and necessitates further detailed analyses.

Since the lowest concentration of the extracts showed the best results, we also tested the potential of extracts at a concentration of 0.05 mg/mL to protect cells from hydrogen peroxide (H_2_O_2_). The extracts of all three groups of broccoli microgreens significantly reduced the intracellular concentration of ROS caused by the H_2_O_2_. This indicated the strong potential of the broccoli microgreen extract at 0.05 mg/mL to protect cells from the oxidative stress caused by H_2_O_2_. Low and high growing temperatures significantly decreased the potential of the extracts to act against H_2_O_2_ damage in MEF, HaCaT, and HepG2 cells. In HCT116 cells, only HT decreased the potential of the extract to scavenge ROS, while the level of ROS in H460 cells treated with RT, HT, and LT extracts did not significantly differ. This suggests that, with regard to ROS, H460 cells are the least sensitive to the changes in broccoli provoked by HT and LT. Among the tested cell types treated by H_2_O_2_, the most significant reduction in ROS (36.61%) was recorded in MEF cells treated with RT broccoli extracts.

### 2.9. Chemometric Analysis

#### 2.9.1. Pearson’s Correlations

Pearson’s correlations allow an insight into the level of the linear relationship between two parameters. Since we recorded significant changes in some of the measured parameters of the broccoli extracts’ bioactivity, we performed a correlation analysis in order to verify the possible correlations between phytochemicals and bioactivity parameters. In interpreting the level of correlation, we were guided by the table offered by Meghanathan [103].

The total phenolics in broccoli extracts showed a very strong (FRAP) or strong (ABTS and DPPH) positive correlation with the antioxidant capacity (Supplementary Table S1). The ABTS results were also very strongly correlated with total flavonols and hydroxycinnamic acids, while the FRAP results were correlated with total proanthocyanidins, tannins, and conjugated sinapic acid.

Conjugated kaempferol had a very strong negative correlation with the level of ROS in HaCaT cells treated with the 0.05 mg/mL extract of broccoli (Supplementary Table S2). The same was recorded for MEF cells exposed to H_2_O_2_ and then a 0.05 mg/mL extract of broccoli. Free kaempferol showed a very strong negative correlation with the level of ROS in HCT 116 cells treated with the 0.05 mg/mL extract of broccoli. These results lead to the assumption that the flavonol kaempferol in broccoli extracts is one of the components responsible for its protective role against ROS in the mentioned cell types. The protective role of kaempferol in the cells treated with H_2_O_2_ has recently been observed in PIG1 normal human skin melanocytes as well [104]. Moreover, it reduced the ROS production in cardiomyocytes [105].

We recorded a strong positive relation between the content of total tannins and quercetin and the in vitro inhibition of lipase activity (Supplementary Table S3). Regarding tannins, the same result has already been obtained from a chemical point of view [106], and these authors offered an explanation in terms of preferential binding to the enzyme–substrate complex. According to them, the hydroxyl groups of tannins were attached to polar amino acid residues by hydrogen bonds. The ability of quercetin to inhibit lipase activity has also already been observed [107]. The binding of quercetin changes the conformation of lipase and consequently decreases the affinity between the substrate and enzyme.

Among the pigments, a very strong negative correlation between chlorophyll *b* and total porphyrins and the concentration of ROS in MEF, HaCaT, and HCT 116 cells treated with the 0.05 mg/mL broccoli extract was detected (Supplementary Table S4).

The total glucosinolates showed a strong positive correlation with the antioxidant capacity of the extracts measured by the FRAP method (Supplementary Table S5) and with the potential to inhibit lipase (Supplementary Table S6).

#### 2.9.2. Principal Component Analysis

**Figure 2:**
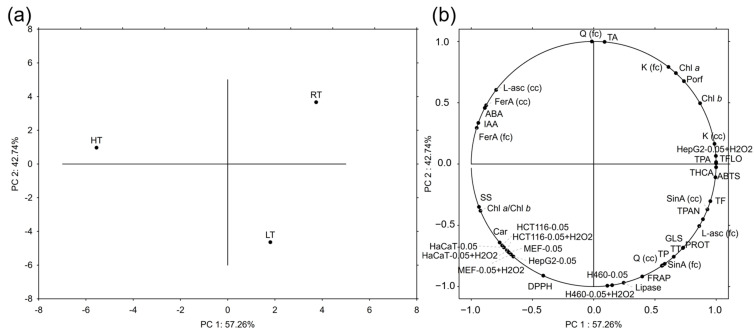
The principal component analysis showing (**a**) the relation between broccoli microgreens grown under room (RT), high (HT), and low (LT) temperatures based on the analyzed variables, whose grouping is shown in the (**b**) part of the figure. ABA = abscisic acid; Car = carotenoids; cc = conjugated compound; Chl = chlorophyll; fc = free compound; FerA= ferulic acid; GLS = total intact glucosinolates; H2O2 = hydrogen peroxide; H460 = lung carcinoma; HaCaT = normal human keratinocytes; HCT116 = colorectal carcinoma; HepG2 = hepatocellular carcinoma; IAA = indole-3-acetic acid; K = kaempferol; L-asc = *L*-ascorbic acid, MEF = mouse embryonal fibroblasts; Porf = porphyrins; PROT = total proteins; Q = quercetin; SinA = sinapic acid; SS = total soluble sugars; TA = total monomeric anthocyanins; TF = total flavonoids; TFlo = total flavonols; THCA = total hydroxycinnamic acids; TP = total phenolics; TPA = total phenolic acids; TPAN = total proanthocyanidins; TT = total tannins.

Principal component analysis (PCA) serves to reduce large data sets and recognize the patterns between them. In this analysis, we incorporated all the data, except for the different concentrations of broccoli extracts applied to the cells. To be more precise, since the best results were accomplished with the extracts with the concentration 0.05 mg/mL, these data were included in the PCA, in order to make the loading plots visually clearer. The ratios of pigments were omitted for the same reason. PC 1 explained 57.26% and PC 2 explained 42.74% of the variance between the measured parameters. A score plot showed the clear separation of each of the broccoli groups tested (Figure 2a). Conjugated quercetin, total anthocyanins, the ROS level in H460 cells treated with 0.05 mg/mL broccoli extract with or without H_2_O_2_ pretreatment, and the potential to inhibit lipase activity contributed most significantly to the separation based on PC 2 (Figure 2b). On the other hand, total hydroxycinnamic acids, phenolic acids, flavonols, the antioxidant capacity measured by the ABTS method, and the level of ROS in HepG2 cells treated with 0.05 mg/mL broccoli extract with H_2_O_2_ pretreatment contributed the most to the separation based on the PC 1. The parameters that contributed the most to the separation of the RT group were conjugated kaempferol, chlorophyll *a* and *b*, and total porphyrins. The separation of the HT group was mostly due to free *L*-ascorbic acid, free and conjugated ferulic acid, the hormones ABA and IAA, soluble sugars, and the chlorophyll *a*/*b* ratio. The LT group was singled out mostly due to the antioxidant capacity determined by the FRAP method, free quercetin, conjugated sinapic acid, total phenolics, tannins, proteins, and glucosinolates.

#### 2.9.3. Hierarchical Clustering

**Figure 3:**
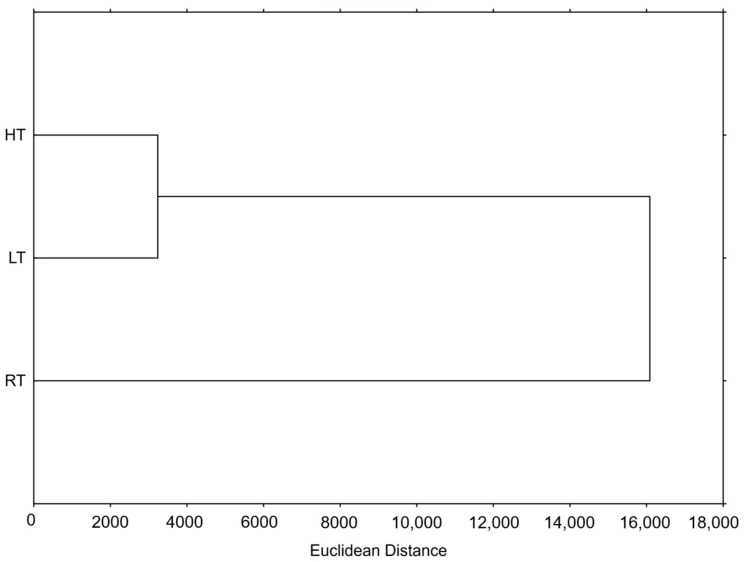
Hierarchical clustering of broccoli microgreens grown at room (RT), high (HT), and low (LT) temperature expressed as Euclidean distance, based on the measured total and individual bioactive compounds, their pigments, their hormones, the ability to inhibit lipase, the antioxidant capacity, and their effect on the intracellular levels of ROS in different cell types.

Hierarchical clustering is used to identify inherent structures or natural groupings within a set of data. Based on their properties, it groups similar data into clusters, making it useful for the discovery patterns or relationships between them. The result of this method is a dendrogram offering a clear and simple visual representation of the relationships within the data. In our study, we were interested in the relationship between the groups of broccoli microgreens grown at RT, HT, and LT (Figure 3). As shown on the dendrogram, the HT and LT groups were much more similar to each other than to the RT. Moreover, the RT was equally separated and far away from these two test groups, suggesting that both HT and LT clearly phytochemically changed the broccoli microgreens as well as the bioactivity of their extracts.

K-means clustering with V-fold cross-validation was used for the characterization of the clusters and inter-cluster differences, based on their phytochemical content and the bioactivity of the extracts (Table 8). Two clusters were identified, and the most discriminative parameters among the phytochemicals were chlorophyll *a* and the conjugated form/s of the flavonoid kaempferol (the highest F value), while, among the bioactivity variables, this was the intracellular level of ROS in MEF, HaCaT, and HCT116 cells treated with H_2_O_2_ and the broccoli extract at 0.05 mg/mL; in MEF cells treated with different concentrations of broccoli extract; in HaCaT cells treated with 0.25 and 0.5 mg/mL of extract; and in HepG2 treated with 0.05 mg/mL extract.

## 3. Materials and Methods

### 3.1. Plant Material

Seeds of the broccoli variety *Brassica oleracea* L. convar. *botrytis* (L.) Alef. var. *cymosa* Duch. (known as broccoli Calabrais), Art. No. 424430, were obtained from International Seeds Processing GmbH (Quedlinburg, Germany). In the autumn of 2022, seeds were planted in sterile soil substrate Stender B400 and placed in a Fito-Clima 600 PLH climate chamber (Aralab, Rio de Mouro—Portugal). The plants were grown under room temperature (RT, 23 °C day/18 °C night) for eleven days, after which the temperature treatment started. Three biological replicates were subjected to the low temperature (LT, 12 °C day/7 °C night), three biological replicates to the high temperature (HT, 38 °C day/33 °C night), and three biological replicates were maintained at RT as a control group. In all biological replicates, the light regime was 16 h (day) and 8 h dark (night), while watering was supervised. The humidity was 65% in all of the groups, except at LT for the last three days, which was 85% due to the temperature regime. Plant material was collected 5 days after the temperature treatment started (i.e., 16 days after planting the seeds) by cutting beneath the lower leaves; it was then rapidly frozen using liquid nitrogen and lyophilized.

### 3.2. Extraction of Phenolic Compounds

Phenolic compounds were extracted from 30 mg of lyophilized plant material using 1 mL of 70% (*v/v*) ethanol. To optimize the extraction, the mixture of plant material and solvent underwent 60-min incubation on a digital tube rotator (Thermo Scientific, Shanghai, China) at 30 rpm at RT. After incubation, the extract was centrifuged at 13,000 rpm for 5 min using a MIKRO 220 centrifuge (Hettich, Westphalia, Germany). The supernatants were separated and stored at −20 °C until further analysis.

### 3.3. Measurement of Different Groups of Polyphenolic Compounds

The total phenolics, flavonoids, phenolic acids, flavonols, hydroxycinnamic acids, proanthocyanidins, and tannins were measured by colorimetry-based methods, using a FLUOstar Optima microplate reader (BMG LABTECH, Ortenberg, Germany) [11]. For the blank, 70% ethanol was used instead of the extract. The content of each of the polyphenolic groups in the samples was calculated indirectly based on the calibration curves of external standard solutions of known concentrations. Total anthocyanins were determined by the pH-differential method. For this purpose, extracts in 70% ethanol were used at pH 4.5 and pH 1.0. After incubation in the dark for 20 min, at room temperature, the absorbance of samples at both pH was measured at 520 and 700 nm. The anthocyanin concentration was calculated according to the following equation:

$$Anthocyanin\;concentration=\frac{A\;\times \;MW\;\times \;DF\;\times \;10^{3}}{\varepsilon \;\times \;l}$$

where A = (A_520_ − A_700_) pH 1.0 − (A_520_ − A_700_) pH 4.5, MW (molecular weight of cyanidin-3-glucoside) = 449.2 g/mol, DF (dilution factor) = 1, 10^3^ = factor for conversion from g to mg, ε (molar extinction coefficient) = 26,900, l = path length in cm. The final results were expressed as mg of cyanidin-3-glucoside equivalent/kg DW.

### 3.4. LC-MS/MS Analysis of Individual Phenolic Compounds and L-Ascorbic Acid

The analysis was conducted on an Agilent 1260 Infinity II liquid chromatograph coupled with an Agilent 6495 LC/TQ triple quadrupole mass spectrometer (Agilent, Santa Clara, CA, USA). Chromatographic separation was achieved on an InfinityLab Poroshell 120 EC-C18 150 cm × 3.0 mm, 2.7 µm column (Agilent, Santa Clara, CA, USA). Data acquisition and processing were carried out using the Agilent MassHunter Quantitative Analysis software v. 10.0. The mobile phase consisted of 0.2% acetic acid in water (A) and 0.2% acetic acid in water/methanol (2:8) (B). The mobile phase gradient was as follows: at 0.50 min 100% A and 0% B, at 12.00 min 20% A and 80% B, at 13.00 min 0% A and 100% B, at 15.00 min 0% A and 100% B, at 15.10 min 100% A and 0% B. The total run time was 17 min. The flow rate was 0.3 mL/min. The injection volume was set at 10 µL. The ESI source was operated in both positive and negative ionization mode with the following instrumental parameters (equal for both mode): gas temperature 150 °C, gas flow 15 L/min, nebulizer 35 psi, sheath gas temperature 370 °C, sheath gas flow 12 L/min, capillary 3500 V, nozzle voltage/charging 1500 V, dwell 15 ms, cell accelerator voltage 4 V. The retention times of compounds, ESI polarity, precursor ions, product ions, and collision energy are given in Supplementary Table S7. The chromatograms of RT, HT, and LT broccoli extracts before and after hydrolysis are given in Supplementary Figures S2–S4.

To hydrolyze the glycosylated phenolic compounds and extract aglycones, acidic hydrolysis was performed as in Šola et al. [11]. For quantitative analyses, calibration curves generated by injecting known concentrations (within the range 1–250 µg/mL) of the mixed standard solution were used. All the standards were of HPLC grade and obtained from Extrasynthese (Genay, France). The results were expressed as μg/g DW.

### 3.5. Measurement of Soluble Proteins and Sugars

The concentration of soluble proteins was determined following the method of Bradford [108]. Extracts were prepared at a concentration of 30 mg/mL in a 50 mM phosphate buffer at pH 7.0 and the color intensity was assessed by measuring the absorbance at 595 nm using a FLUOstar Optima (BMG Labtech, Offenburg, Germany) microplate reader. A blank was prepared using 50 mM phosphate buffer at pH 7.0 instead of the extract. The concentration of proteins was calculated based on the calibration curve generated from standard bovine serum albumin solutions of known concentrations (0.3–1.7 mg/mL). The results were expressed as mg/g DW.

For the measurement of total soluble sugars, extracts in 70% ethanol were used and the method by Dubois et al. [109] was applied. The concentration of total soluble sugars was calculated indirectly based on the calibration curve generated from standard saharose solutions of known concentrations (0.05–1.0 mg/mL). The results were expressed as mg/g DW.

### 3.6. Measurement of Total Intact Glucosinolates

The concentration of total intact glucosinolates was determined following the method described by Aghajanzadeh et al. [110]. Extracts at a concentration of 30 mg/mL were prepared in 70% methanol (90 °C), shaken on a vortex mixer for 10 s, and then incubated for 3 min in a Thermomixer at 90 °C. Afterwards, the extracts underwent centrifugation for 5 min at 13,000 rpm, and the supernatants were taken. The absorbance was measured at 405 nm using a FLUOstar Optima microplate reader. A blank was prepared using hot 70% methanol instead of the extract. The concentration of total intact glucosinolates was calculated indirectly based on the calibration curve generated from standard sinigrin aqueous solutions of known concentrations (0.1–1.0 mg/mL). The results were expressed as mg/g DW.

### 3.7. Measurement of Photosynthetic Pigments

The extracts were prepared in 80% acetone and the concentration of photosynthetic pigments was determined following the method described by Sumanta et al. [111]. The absorbance of the extract was monitored at 470, 575, 590, 628, 647, and 663 nm on a Nanodrop 2000c (Thermo Fisher Scientific Inc., Waltham, MA, USA) spectrophotometer. The results were expressed as mg/g DW.

### 3.8. GC-MS Analysis of IAA and ABA

Indole-3-acetic acid (IAA) and abscisic acid (ABA) extraction followed the protocol described by Ludwig-Müller et al. [112]. The analysis, using gas chromatography–mass spectrometry (GC-MS), was performed on a Varian Saturn 2100 ion-trap mass spectrometer (SpectraLab Scientific Inc., Markham, ON, Canada) with electron impact ionization at 70 eV, coupled to a Varian CP-3900 gas chromatograph. For the analysis, 1 µL of the methylated sample, dissolved in ethyl acetate, was injected in splitless mode (splitter opening 1:100 after 1 min) onto a Phenomenex ZB-5 column (30 m × 0.25 mm × 0.25 µm), using helium as the carrier gas, at a flow rate of 1 mL/min. The identification of IAA and ABA relied on a retention time comparison with an authentic methylated standard and the recording of the corresponding mass spectrum. Subsequently, the peak area of the mass was recorded, and the concentration of the target compound was determined by assessing the ratio between the masses of the endogenous and standard forms. The distinctive ions used for IAA were 130/136 (endogenous and heavy-labeled); for ABA, they were 190/194. The chromatograms of standard compounds and a selected sample are given in Supplementary Figure S5.

### 3.9. Determination of Antioxidant Capacity

The antioxidant capacity was measured using the ABTS [113], DPPH [114], and FRAP [115] assays. For the ABTS and DPPH assays, extracts with a concentration of 30 mg/mL in 70% ethanol were employed, while, for the FRAP assay, extracts with a concentration of 15 mg/mL in 70% ethanol were used. For the blank, 70% ethanol was used instead of the extract. The results are presented as a percentage of the value obtained for a Trolox solution with the same concentration as the extract.

### 3.10. Measurement of Intracellular Levels of Reactive Oxygen Species

Intracellular ROS levels were determined on mouse embryonal fibroblasts (MEF, established in the laboratory of Dr. Pinteric from mice RRID:IMSR_JAX:002448, Jackson Lab, Bar Harbor, ME, USA), normal human keratinocytes (HaCaT, RRID: CVCL-0038, a gift from Dr. Marijeta Kralj), hepatocellular carcinoma (HepG2, ATCC, HB-8065, Manassas, VA USA), colorectal carcinoma (HCT116, ATCC, CCL-247, Manassas, VA, USA), and lung carcinoma (H460, ATCC, HTB-177, Manassas, VA, USA) cell lines using 2′,7′-dichlorodihydrofluorescein diacetate (H2DCFDA, cat. no. D6883, Sigma Aldrich, Saint Louis, MO, USA). Cells were maintained in high-glucose Dulbecco’s modified Eagle medium (DMEM, Sigma-Aldrich, St. Louis, MO, USA) supplemented with 10% fetal bovine serum (FBS, Sigma-Aldrich, St. Louis, MO, USA), 1% nonessential amino acids (Sigma-Aldrich, St. Louis, MO, USA), and 1% antibiotic/antimycotic solution (Capricorn Scientific GmbH, Ebsdorfergrund, Germany) in a humidified atmosphere with 5% CO_2_ at 37 °C. To determine the intracellular levels of ROS, cells were seeded in 96-well plates at a density of 5 × 10^4^ cells per well 24 h before measurement. After 24 h, the growth medium was removed and replaced with 10 µM H2DCFDA dissolved in serum-free DMEM medium and incubated for 45 min. Subsequently, the cells were treated with different concentrations of the test extracts diluted in PBS. Working dilutions were freshly prepared on the day of testing (0.05–0.5 mg/mL). Since the 0.05 mg/mL extract concentration had the strongest antioxidant effect, we tested its effect on ROS production in combination with 1 mM hydrogen peroxide (H_2_O_2_). The cells treated with 1 mM H_2_O_2_ were used as a positive control, and cells untreated and incubated only in PBS were used as a negative control. DCF fluorescence was measured after 60 min of treatment using a Tecan Spark microplate reader (Tecan Life Siences, Zurich, Switzerland) at a maximum excitation level of 485 nm and emission level of 535 nm.

### 3.11. Measurement of Broccoli Extracts’ Potential to Inhibit Enzymes α-Amylase and Lipase

The potential to inhibit α-amylase activity was measured as in Šola et al. [11] and to inhibit pancreatic lipase according to Spínola et al. [116]. As a positive control, acarbose and orlistat were applied, respectively. The negative control was the solvent in which the extract was prepared, 70% ethanol. For both analyses, the percentage of enzyme inhibition was calculated from the equation

$$\%\;inhibition=\;\left(1-\frac{At-Atb}{Ac-Acb}\right)\times 100$$

where At was the absorbance of the test substance (with enzyme), Atb was the absorbance of the test blank (without enzyme), Ac was the absorbance of the control (with enzyme), and Acb was the absorbance of the control blank (without enzyme). The absorbance measurements were performed with a FluostarOptima microplate reader (BMG Labtech GmbH, Offenburg, Germany).

### 3.12. Statistical Analysis

The statistical analysis of the data was conducted using the Statistica 14.0 program (TIBCO Software Inc., Palo Alto, CA, USA). The average values of multiple samples were compared through one-way variance analysis (ANOVA) and Duncan’s new multiple range test (DNMRT), while, in the case of two samples’ comparison, Student’s *t*-test was applied. Statistically significant differences were considered at the *p* ≤ 0.05 level. To assess the similarity or dissimilarity of samples based on their phytochemical and antioxidant properties, multivariate principal component analysis (PCA), hierarchical clustering utilizing the Euclidean distance between samples, and single linkage clustering were performed. To characterize the clusters and inter-cluster differences, we applied the k-means clustering algorithm with the V-fold cross-validation method. Pearson’s linear correlation coefficients were calculated to determine the relationships between the phytochemical compounds and bioactivity values.

## 4. Conclusions

Climate change is leading to shifts in temperature patterns, and plants need to adjust to these changes to survive and thrive. Studies into how temperature stress influences the acclimation of plants to climate change are crucial in developing strategies to support plant resilience and productivity in the face of ongoing environmental change. The results of our work revealed that broccoli microgreens, when grown under LT and HT, significantly change their phytochemical profiles and, as a consequence, the biological effects of their extracts. For example, growth under LT conditions significantly increased the total phenolics, tannins, and glucosinolates. This suggests that phenolics, specifically tannins, and glucosinolates play a crucial role in defense of broccoli against LT. On the other hand, HT decreased the total glucosinolates. Soluble sugars and osmoprotectants were increased by both types of stress, considerably more so by HT than LT, suggesting that HT causes a more severe osmotic imbalance. Both temperatures were detrimental for chlorophyll, and it was also more sensitive to HT than LT. Chlorophyll *b* was more sensitive to temperature stress than chlorophyll *a*, and notably more susceptible to HT than LT. HT significantly increased the concentration of the hormone indole-3-acetic acid, implying an important role in broccoli’s defense against HT. Ferulic and sinapic acids showed a trade-off scheme. Ferulic acid was significantly increased by HT and decreased by LT, while sinapic acid was decreased by HT and increased by LT. The antioxidant capacity of the broccoli extracts was significantly increased by LT (measured by DPPH and FRAP); however, it was decreased by HT (measured by ABTS method). Extracts of all three groups of broccoli microgreens significantly reduced the intracellular concentrations of ROS caused by H_2_O_2_. However, HT and LT significantly decreased this potential of broccoli extracts in mouse embryonal fibroblasts (MEF), human keratinocytes (HaCaT), and human liver cancer (HepG2) cells. Human lung cancer (H460) cells were the least sensitive to the changes in broccoli provoked by HT and LT. In general, among the tested cell types treated with H_2_O_2_, the most significant reduction in ROS (36.61%) was recorded in MEF cells treated with RT broccoli extracts. Both temperature stresses increased the potential of the broccoli extracts to inhibit α-amylase; however, the inhibition of pancreatic lipase was increased by LT only. Based on all the collected data, we conclude that both HT and LT significantly changed the broccoli microgreens at the level of the analyzed parameters, by 82% and 81%, respectively. Among the changed parameters, HT increased 44% of them, while LT increased 55%. The parameters that were significantly changed by HT, but not LT, were the total proanthocyanidins, kaempferol, sinapic acid, the hormone IAA, the antioxidant capacity measured by the ABTS method, and the concentration of ROS in HepG2 cells treated with H_2_O_2_ and the broccoli extract at 0.05 mg/mL. On the other hand, the parameters significantly affected by LT, but not HT, were the total proteins and tannins, the antioxidant capacity measured by the DPPH and FRAP methods, and the potential to inhibit the enzyme lipase. We assume that, among these parameters that were responsive to one but not the other temperature stress type, we could search for mediators that are crucial for plants’ adjustment to HT/LT stress. Based on this, further, more detailed studies of the mentioned intermediates under the influence of temperature stress are necessary. From the perspective of nutritional value, and based on the obtained results, we conclude that LT conditions result in more nutritious broccoli microgreens than HT.

## Figures and Tables

**Table 1:** Effects of high and low growing temperatures on polyphenolics in broccoli microgreens.

	RT	HT	LT	ΔHT (%)	ΔLT (%)
TP (mg GAE/g DW)	15.41 ± 1.16 b	14.41 ± 1.02 c	17.76 ± 0.92 a	−6	15
TF (mg QE/g DW)	22.66 ± 0.74 a	19.84 ± 1.42 b	23.05 ± 1.14 a	−12	2
TPA (mg CAE/g DW)	18.30 ± 0.62 a	16.55 ± 1.60 b	17.92 ± 1.14 a	−10	−2
THCA (mg CAE/g DW)	15.62 ± 1.01 a	12.66 ± 0.25 b	15.08 ± 0.92 a	−19	−3
TFlo (mg QE/g DW)	16.78 ± 1.27 a	13.48 ± 0.27 c	16.04 ± 0.94 b	−20	−4
TA (mg C3gE/kg DW)	25.07 ± 3.82 a	16.91 ± 5.49 b	4.88 ± 1.08 c	−33	−81
TT (mg GAE/g DW)	5.86 ± 1.24 b	4.99 ± 0.95 b	7.27 ± 1.43 a	−15	24
TPAN (mg CcE/g DW)	1.73 ± 0.14 a	1.56 ± 0.05 b	1.79 ± 0.12 a	−9	4

Values represent average ± standard deviation of three biological and three technical replicates. Different letters indicate a significant difference among the values in a row (ANOVA, Duncan test, *p* ≤ 0.05). DW = dry weight; RT = room temperature; HT = high temperature; LT = low temperature; C3gluE = cyanidin-3-glucoside equivalent; CAE = caffeic acid equivalent; CcE = cyanidin chloride equivalent; GAE = gallic acid equivalent; QE = quercetin equivalent; TA = total monomeric anthocyanins; TF = total flavonoids; TFlo = total flavonols; THCA = total hydroxycinnamic acids; TP = total phenolics; TPA = total phenolic acids; TPAN = total proanthocyanidins; TT = total tannins.

**Table 2:** Effects of high and low growing temperatures on total glucosinolates, proteins, and sugars in broccoli microgreens.

	RT	HT	LT	ΔHT (%)	ΔLT (%)
GLS (mg SinE/g DW)	42.62 ± 4.22 b	34.97 ± 3.37 c	51.06 ± 3.20 a	−18	20
PROT (mg BSAE/g DW)	51.35 ± 2.54 ab	49.10 ± 3.39 b	53.87 ± 3.02 a	−4	5
SS (mg SucE/g DW)	30.44 ± 1.72 c	72.17 ± 3.74 a	52.53 ± 7.19 b	137	73

Values represent average ± standard deviation of three biological and three technical replicates. Different letters indicate a significant difference among the values in a row (ANOVA, Duncan test, *p* ≤ 0.05). DW = dry weight; RT = room temperature; HT = high temperature; LT = low temperature; BSAE = bovine serum albumin equivalent; GLS = total intact glucosinolates; PROT = total proteins; SucE = sucrose equivalent; SinE = sinigrin equivalent; SS = total soluble sugars.

**Table 3:** Effects of high and low growing temperatures on photosynthetic pigments in broccoli microgreens.

mg/g DW	RT	HT	LT	ΔHT (%)	ΔLT (%)
Chl *a*	4.95 ± 0.17 a	4.79 ± 0.14 b	4.79 ± 0.18 b	−3	−3
Chl *b*	3.67 ± 0.25 a	2.84 ± 0.13 c	3.12 ± 0.16 b	−23	−15
Chl *a* + Chl *b*	8.63 ± 0.26 a	7.63 ± 0.15 c	7.91 ± 0.22 b	−12	−8
Chl *+* Car	9.39 ± 0.22 a	8.53 ± 0.16 c	8.78 ± 0.22 b	−9	−7
Chl *a*/*b*	1.36 ± 0.12 c	1.69 ± 0.11 a	1.54 ± 0.11 b	24	14
Chl/(Chl + Car)	0.92 ± 0.01 a	0.90 ± 0.00 c	0.90 ± 0.01 b	−3	−2
Car/(Chl + Car)	0.08 ± 0.01 c	0.10 ± 0.00 a	0.10 ± 0.01 b	28	22
Chl/Car	11.46 ± 1.64 a	8.56 ± 0.43 b	9.11 ± 0.72 b	−25	−21
Car	0.77 ± 0.09 b	0.89 ± 0.04 a	0.87 ± 0.06 a	17	14
Por	10.26 ± 0.50 a	8.83 ± 0.23 b	8.99 ± 0.32 b	−14	−12

Values represent average ± standard deviation of three biological and three technical replicates. Different letters indicate a significant difference among the values in a row (ANOVA, Duncan test, *p* ≤ 0.05). DW = dry weight; RT = room temperature; HT = high temperature; LT = low temperature; Chl = chlorophyll; Car = carotenoids; Por = porphyrins.

**Table 4:** Effects of high and low growing temperatures on IAA and ABA concentrations in broccoli microgreens.

µg/g DW	RT	HT	LT	ΔHT (%)	ΔLT (%)
IAA	94.38 ± 7.12 b	168.03 ± 11.36 a	81.06 ± 14.47 b	78	−14
ABA	14.12 ± 7.36 a	17.48 ± 6.37 a	12.80 ± 4.97 a	24	−9

Values represent average ± standard deviation of three biological and three technical replicates. Different letters indicate a significant difference among the values in a row (ANOVA, Duncan test, *p* ≤ 0.05). DW = dry weight; RT = room temperature; HT = high temperature; LT = low temperature; ABA = abscisic acid; IAA = indole-3-acetic acid.

**Table 5:** Effects of high and low growing temperature on free and derivatized *L*-ascorbic acid, individual flavonoids, and phenolic acids in broccoli microgreens.

µg/g DW	RT	HT	LT	ΔHT (%)	ΔLT (%)
*before hydrolysis*					
*L*-Ascorbic acid	191.57 ± 51.86 b	252.97 ± 16.07 a	144.13 ± 20.73 c	32	−25
Ferulic acid	0.25 ± 0.03 b	0.40 ± 0.02 a	0.19 ± 0.00 c	58	−25
Sinapic acid	0.42 ± 0.03 a	0.36 ± 0.01 b	0.43 ± 0.01 a	−14	3
Quercetin	0.04 ± 0.00 b	0.03 ± 0.00 c	0.05 ± 0.00 a	−11	29
Kaempferol	0.19 ± 0.07 a	0.04 ± 0.01 b	0.14 ± 0.03 a	−77	−28
*after hydrolysis*					
*L*-Ascorbic acid	2978.66 ± 319.05 b	2145.22 ± 425.17 c	3392.51 ± 319.05 a	−28	14
Ferulic acid	33.31 ± 5.05 b	66.10 ± 2.17 a	29.28 ± 0.74 c	98	−12
Sinapic acid	315.90 ± 5.92 b	281.95 ± 28.85 c	406.98 ± 8.78 a	−11	29
Quercetin	120.95 ± 19.00 a	117.37 ± 4.16 a	109.46 ± 11.11 a	−3	−10
Kaempferol	246.16 ± 19.41 a	218.46 ± 10.29 b	216.25 ± 12.73 b	−11	−12

Values represent mean ± standard deviation of three biological and three technical replicates. Different letters indicate a significant difference among the values in a row (ANOVA, Duncan test, *p* ≤ 0.05). DW = dry weight; RT = room temperature; HT = high temperature; LT = low temperature.

**Table 6:** Effects of high and low growing temperatures on antioxidant capacity of broccoli microgreen extracts.

	RT	HT	LT	ΔHT (%)	ΔLT (%)
ABTS (inhibition %)	90.14 ± 4.03 a	80.85 ± 4.62 b	90.12 ± 4.37 a	−11	−1
DPPH (inhibition %)	90.47 ± 1.95 b	91.47 ± 1.74 ab	91.91 ± 1.23 a	1	2
FRAP (reduction %)	94.98 ± 0.36 b	94.89 ± 0.37 b	95.86 ± 0.27 a	0	1

Values represent average ± standard deviation of three biological and three technical replicates. Different letters indicate a significant difference among the values in a row (ANOVA, Duncan test, *p* ≤ 0.05). RT = room temperature; HT = high temperature; LT = low temperature.

**Table 7:** Effects of high and low growing temperatures on the potential of broccoli microgreen extracts to inhibit the activity of the enzymes α-amylase and lipase.

% Inhibition	RT	HT	LT	ΔHT (%)	ΔLT (%)
α-amylase	0.00 ± 0.00 b	7.92 ± 2.40 a	7.68 ± 3.81 a		
lipase	45.36 ± 2.33 b	46.10 ± 2.96 b	54.09 ± 3.12 a	2	19

Values represent mean ± standard deviation of three biological and three technical replicates. Different letters indicate a significant difference among the values in a row (ANOVA, Duncan test, *p* ≤ 0.05). RT = room temperature; HT = high temperature; LT = low temperature.

**Table 8:** Results of k-means clustering, with the number of clusters determined using the V-fold cross-validation method.

	Cluster Mean		Analysis of Variance	
Case	Cluster 1	Cluster 2	F	*p*
MEF-0.25 *	17,796.83	23,549.84	32,406.80	0.00
HaCaT-0.25 *	20,639.70	24,245.36	29,235.43	0.00
HepG2-0.05 *	21,103.03	23,826.83	18,804.47	0.00
Chl *a* *	4.95	4.79	5865.32	0.01
MEF-0.1 *	15,641.86	21,061.29	5015.17	0.01
MEF-0.05 + H₂O₂ *	28,783.08	30,902.20	2673.92	0.01
HaCaT-0.5 *	22,561.20	25,671.24	1629.18	0.02
MEF-0.5 *	20,327.11	25,728.13	1192.34	0.02
MEF-0.05 *	14,525.33	20,661.07	594.52	0.03
HaCaT-0.05 + H₂O₂ *	33,772.45	34,864.18	286.97	0.04
HCT116-0.05 + H₂O₂ *	30,068.17	32,322.82	233.08	0.04
K (fc) *	246.16	217.35	227.49	0.04
HCT116-0.1	20,169.42	23,708.55	119.97	0.06
HaCaT-0.05	16,048.56	20,544.28	99.96	0.06
HaCaT-0.1	18,610.00	22,684.88	89.16	0.07
Porph	10.26	8.91	89.08	0.07
HCT116-0.05	18,639.55	21,864.16	67.91	0.08
HepG2-0.5	26,291.50	28,517.43	53.21	0.09
HCT116-0.25	22,405.28	25,564.42	47.62	0.09
Car	0.77	0.88	42.21	0.10
Chl/Car	11.46	8.83	30.88	0.11
HCT116-0.5	24,697.39	27,504.79	23.32	0.13
Chl/(Chl + Car)	0.92	0.90	19.35	0.14
Car/(Chl + Car)	0.08	0.10	19.35	0.14
Chl	8.63	7.77	12.91	0.17
Chl + Car	9.39	8.65	11.25	0.18
DPPH	90.47	91.69	10.54	0.19
Chl *b*	3.67	2.98	8.58	0.21
HepG2-0.25	24,527.00	26,277.50	6.91	0.23
Chl*a*/Chl*b*	1.36	1.61	4.15	0.29
SS	30.44	62.35	3.52	0.31
H460-0.25	26,628.28	26,988.47	3.09	0.33
HepG2-0.1	22,605.25	24,556.04	3.03	0.33
ANT	25.07	10.90	1.85	0.40
K (cc)	0.19	0.09	1.51	0.43
Q (fc)	120.95	113.41	1.21	0.47
HepG2-0.05 + H_2_O_2_	34,349.72	32,643.21	1.01	0.50
TFLO	16.78	14.76	0.83	0.53
H460-0.05 + H₂O₂	33,644.20	34,471.96	0.82	0.53
TPA	18.30	17.24	0.79	0.54
THCA	15.62	13.87	0.70	0.56
H460-0.05	23,383.86	24,004.99	0.70	0.56
H460-0.1	24,733.67	25,194.76	0.65	0.57
ABTS	91.14	85.48	0.50	0.61
Lipase	45.36	50.10	0.47	0.62
FRAP	94.98	95.37	0.22	0.72
FerA (fc)	33.31	47.69	0.20	0.73
TF	22.66	21.44	0.19	0.74
H460-0.5	28,406.00	28,523.38	0.17	0.75
IAA	94.38	124.55	0.16	0.76
SinA (cc)	0.42	0.40	0.13	0.78
SinA (fc)	315.90	344.46	0.07	0.84
TPAN	1.73	1.68	0.07	0.84
Q (cc)	0.04	0.04	0.07	0.84
ABA	14.12	15.14	0.06	0.84
TP	15.41	16.09	0.05	0.85
FerA (cc)	0.25	0.29	0.05	0.86
*L*-asc (fc)	2.98	2.77	0.04	0.88
TT	5.86	6.13	0.02	0.91
L-asc (cc)	0.19	0.20	0.01	0.95
PROT	51.35	51.49	0.00	0.98
GLS	42.62	43.01	0.00	0.98

* Asterix (*) indicates the significant cases (*p* < 0.05) that discriminate samples into two groups. ABA = abscisic acid; Car = carotenoids; cc = conjugated compound; Chl = chlorophyll; fc = free compound; FerA= ferulic acid; GLS = total intact glucosinolates; H_2_O_2_ = hydrogen peroxide; H460 = lung carcinoma; HaCaT = normal human keratinocytes; HCT116 = colorectal carcinoma; HepG2 = hepatocellular carcinoma; IAA = indole-3-acetic acid; K = kaempferol; L-asc = *L*-ascorbic acid, MEF = mouse embryonal fibroblasts; Porph = porphyrins; PROT = total proteins; Q = quercetin; SinA = sinapic acid; SS = total soluble sugars; TA = total monomeric anthocyanins; TF = total flavonoids; TFlo = total flavonols; THCA = total hydroxycinnamic acids; TP = total phenolics; TPA = total phenolic acids; TPAN = total proanthocyanidins; TT = total tannins.

## Data Availability

The data that support the findings of this study are available from the corresponding author, I.Š., upon request.

## Supplementary Materials

The following supporting information can be downloaded at: https://www.mdpi.com/article/10.3390/ijms25073677/s1.
