# Supplementary material for: Adjustments of the Phytochemical Profile of Broccoli to Low and High Growing Temperatures: Implications for the Bioactivity of Its Extracts
Source: Int J Mol Sci. 2024 Mar 26;25(7):3677. doi: 10.3390/ijms25073677 (PMC11011926; doi:10.3390/ijms25073677)
Supplement: Supplementary file 1 [file ijms-25-03677-s001.zip › Figure S1.pdf]

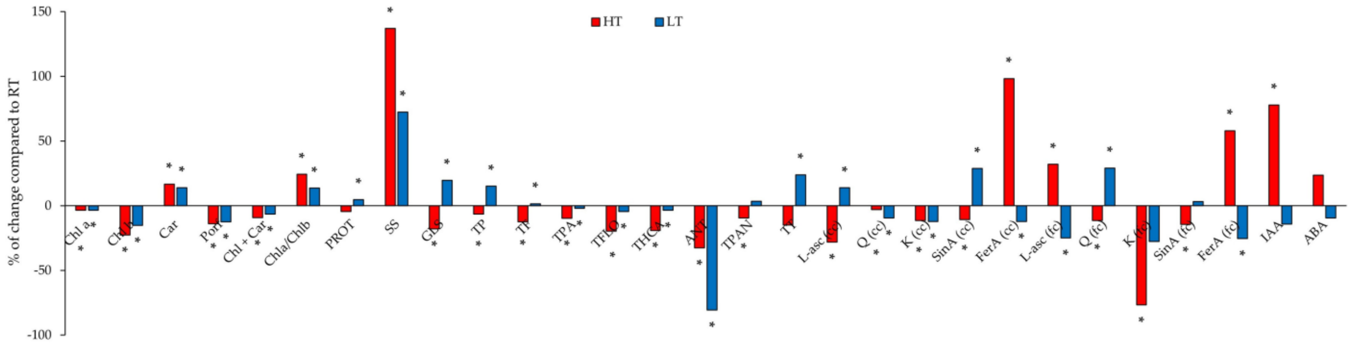

Figure S1. Intensity, expressed in % of change compared to control, and direction of change of phytochemical parameters in broccoli microgreens under the influence of high (HT) and low (LT) growing temperature.
