# Supplementary material for: Adjustments of the Phytochemical Profile of Broccoli to Low and High Growing Temperatures: Implications for the Bioactivity of Its Extracts
Source: Int J Mol Sci. 2024 Mar 26;25(7):3677. doi: 10.3390/ijms25073677 (PMC11011926; doi:10.3390/ijms25073677)
Supplement: Supplementary file 1 [file ijms-25-03677-s001.zip › Figure S2.pdf]

(a)

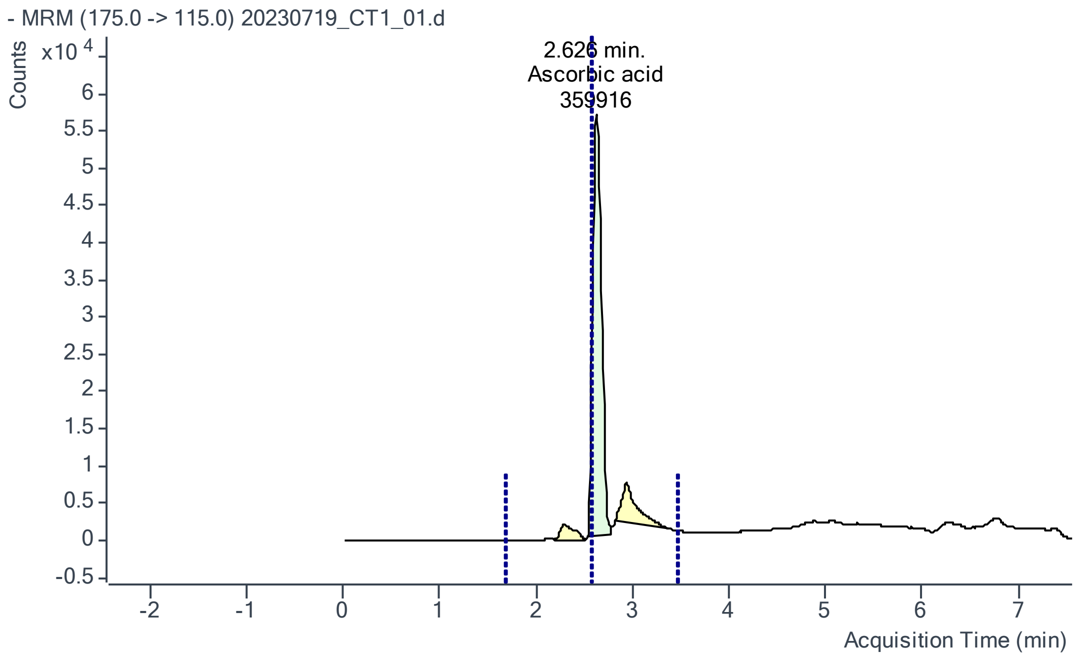
- MRM (175.0 -> 115.0) 20230719_CT1_01.d
Counts
x10 4
2.626 min.
Ascorbic acid
359916
6
5.5
5
4.5
4
3.5
3
2.5
2
1.5
1
0.5
0
-0.5
-2
-1
0
1
2
3
4
5
6
7
Acquisition Time (min)

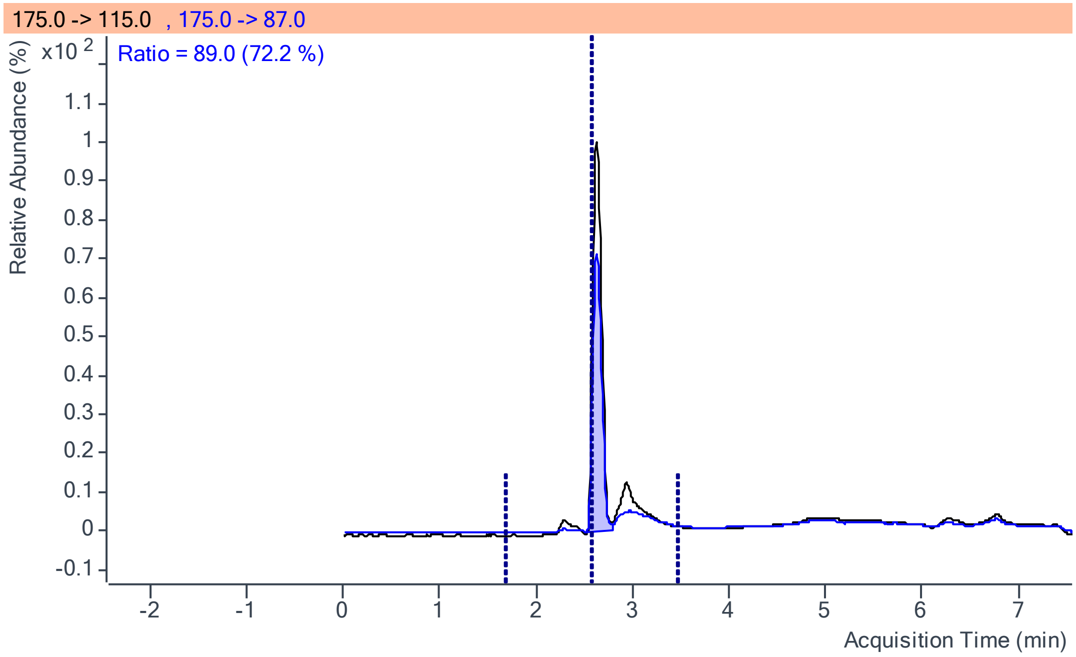
175.0 -> 115.0
, 175.0 -> 87.0
x10 2
Ratio = 89.0 (72.2 %)
Relative Abundance (%)
1.1
1
0.9
0.8
0.7
0.6
0.5
0.4
0.3
0.2
0.1
0
-0.1
-2
-1
0
1
2
3
4
5
6
7
Acquisition Time (min)

(b)

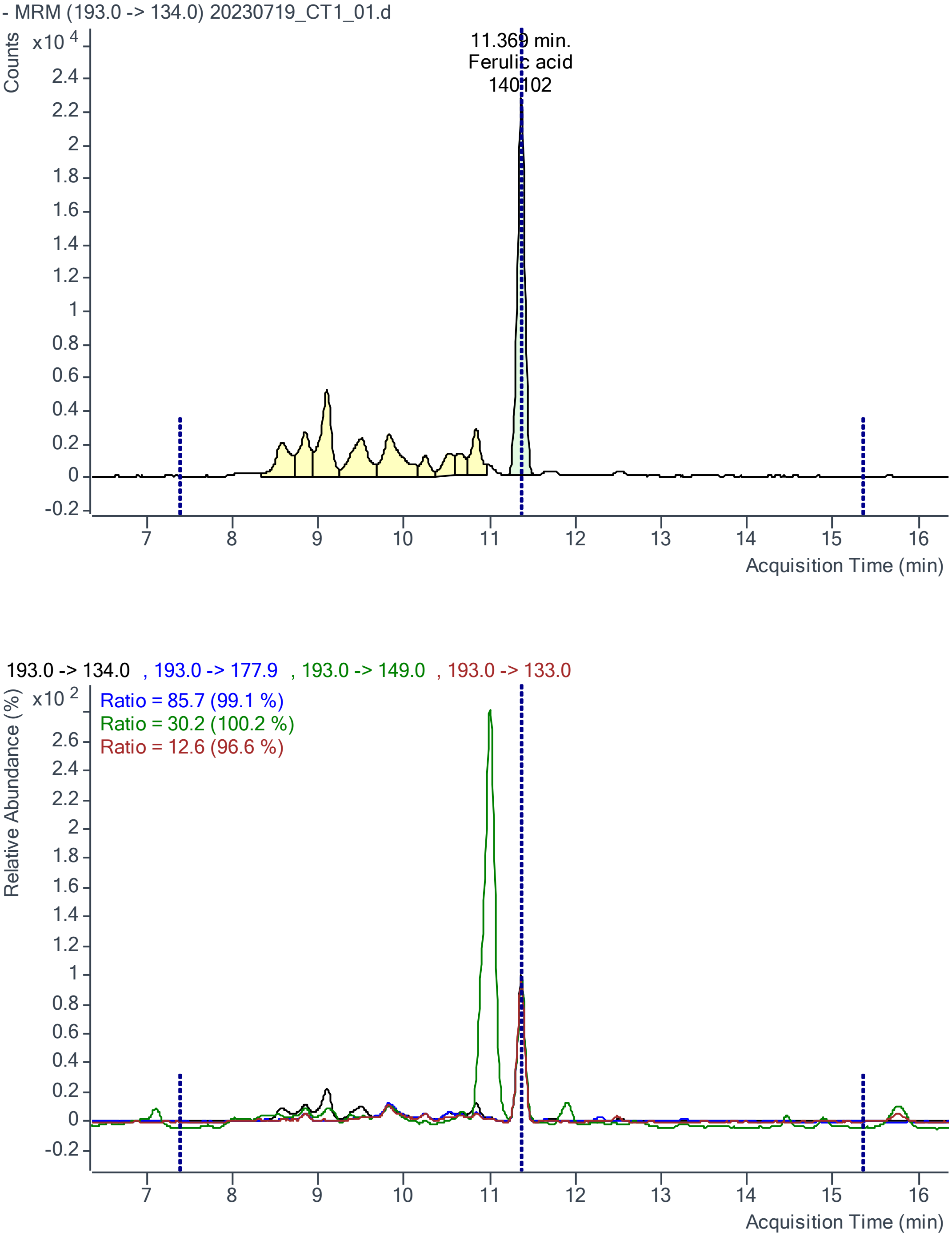
- MRM (193.0 -> 134.0) 20230719_CT1_01.d
Counts
x10 4
11.369 min.
Ferulic acid
140102
Acquisition Time (min)
193.0 -> 134.0 , 193.0 -> 177.9 , 193.0 -> 149.0 , 193.0 -> 133.0
Relative Abundance (%)
x10 2
Ratio = 85.7 (99.1 %)
Ratio = 30.2 (100.2 %)
Ratio = 12.6 (96.6 %)
Acquisition Time (min)

(c)

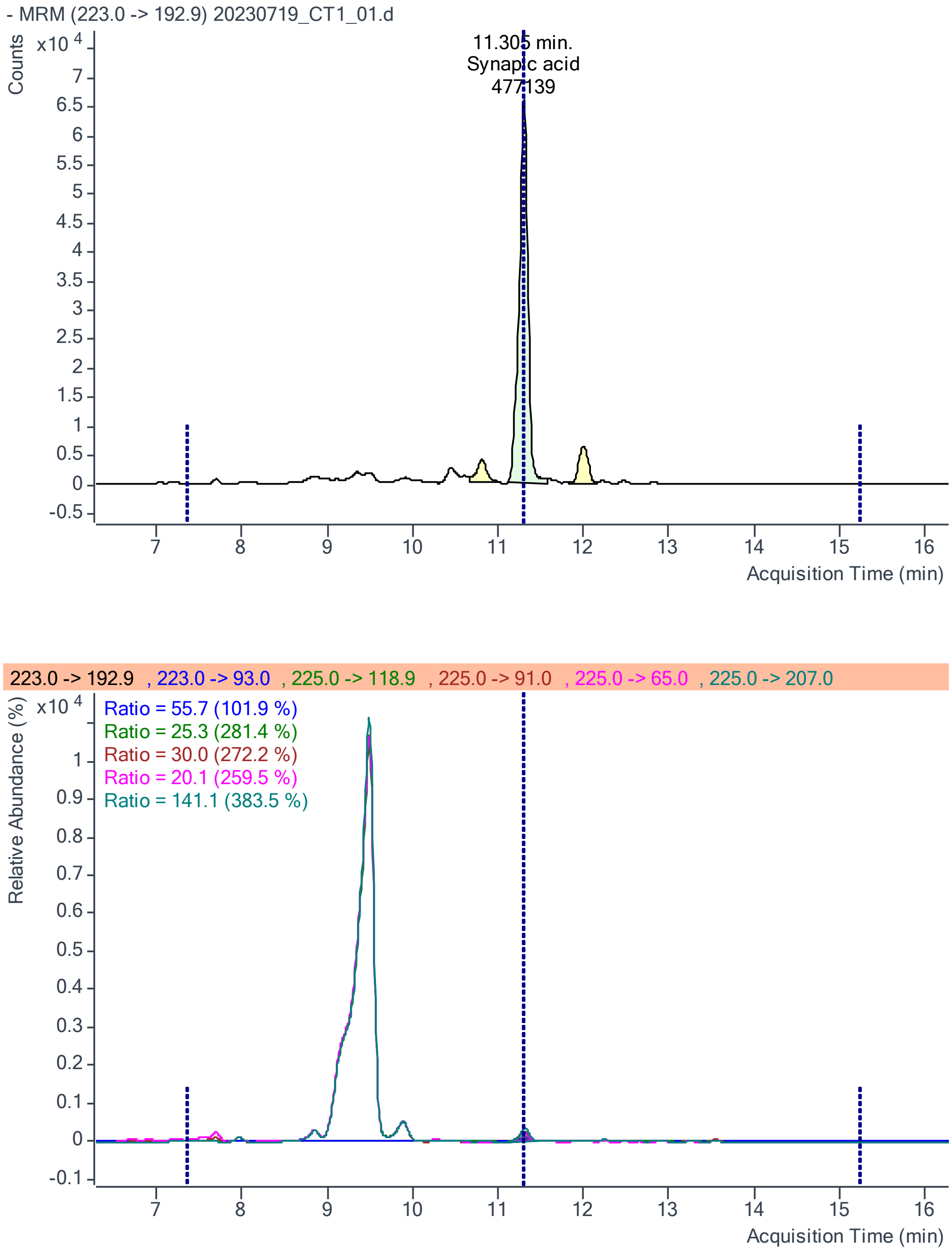
- MRM (223.0 -> 192.9) 20230719_CT1_01.d
Counts
x10 4
11.305 min.
Synaptic acid
477139
Acquisition Time (min)
223.0 -> 192.9 , 223.0 -> 93.0 , 225.0 -> 118.9 , 225.0 -> 91.0 , 225.0 -> 65.0 , 225.0 -> 207.0
Relative Abundance (%)
x10 4
Ratio = 55.7 (101.9 %)
Ratio = 25.3 (281.4 %)
Ratio = 30.0 (272.2 %)
Ratio = 20.1 (259.5 %)
Ratio = 141.1 (383.5 %)
Acquisition Time (min)

(d)

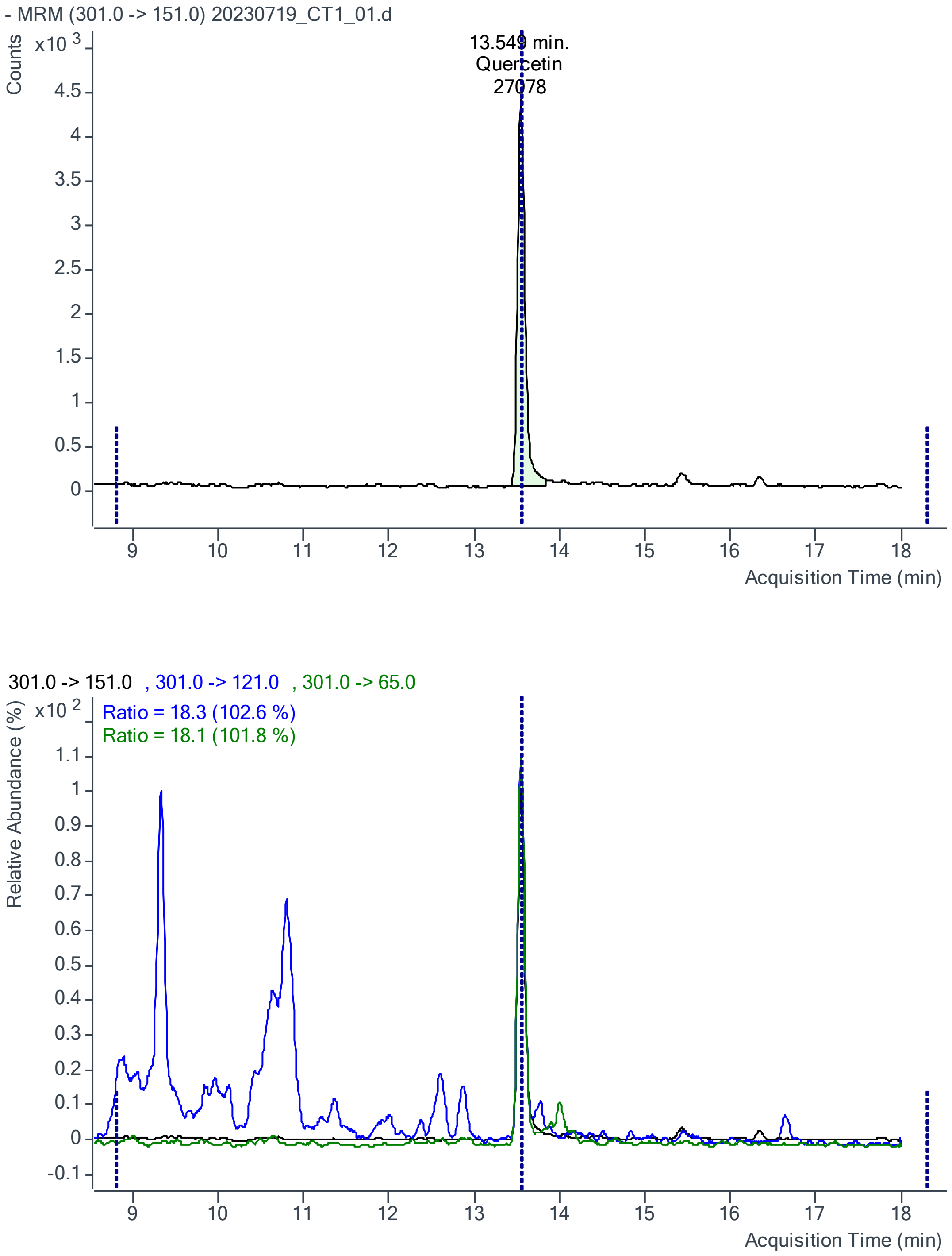
- MRM (301.0 -> 151.0) 20230719_CT1_01.d
Counts
x10 3
13.549 min.
Quercetin
27078
Acquisition Time (min)
301.0 -> 151.0 , 301.0 -> 121.0 , 301.0 -> 65.0
x10 2
Ratio = 18.3 (102.6 %)
Ratio = 18.1 (101.8 %)
Relative Abundance (%)
Acquisition Time (min)

(e)

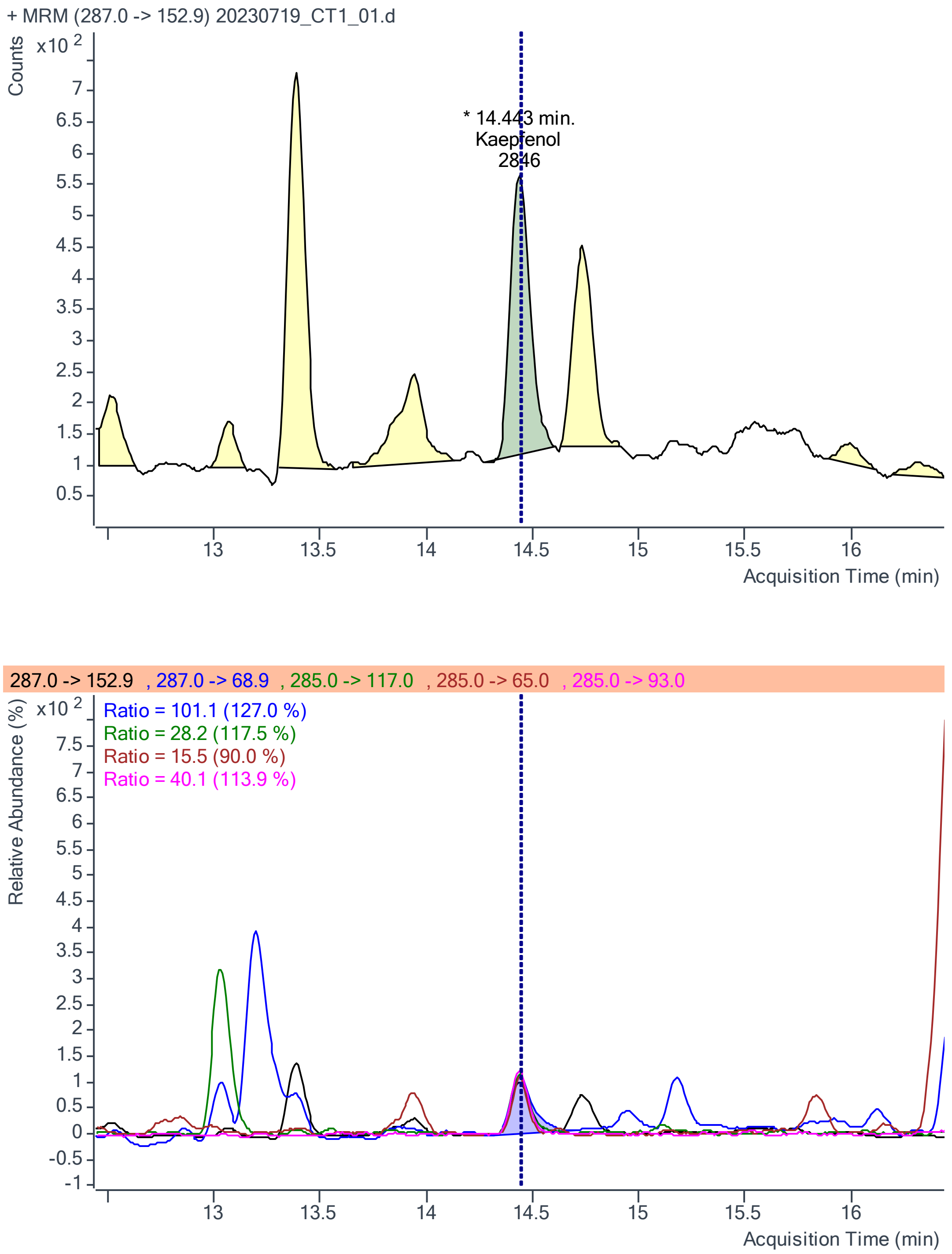
+ MRM (287.0 -> 152.9) 20230719_CT1_01.d
Counts
x10 2
* 14.443 min.
Kaepfenol
2846
Acquisition Time (min)
287.0 -> 152.9 , 287.0 -> 68.9 , 285.0 -> 117.0 , 285.0 -> 65.0 , 285.0 -> 93.0
Relative Abundance (%)
Ratio = 101.1 (127.0 %)
Ratio = 28.2 (117.5 %)
Ratio = 15.5 (90.0 %)
Ratio = 40.1 (113.9 %)
Acquisition Time (min)

(f)

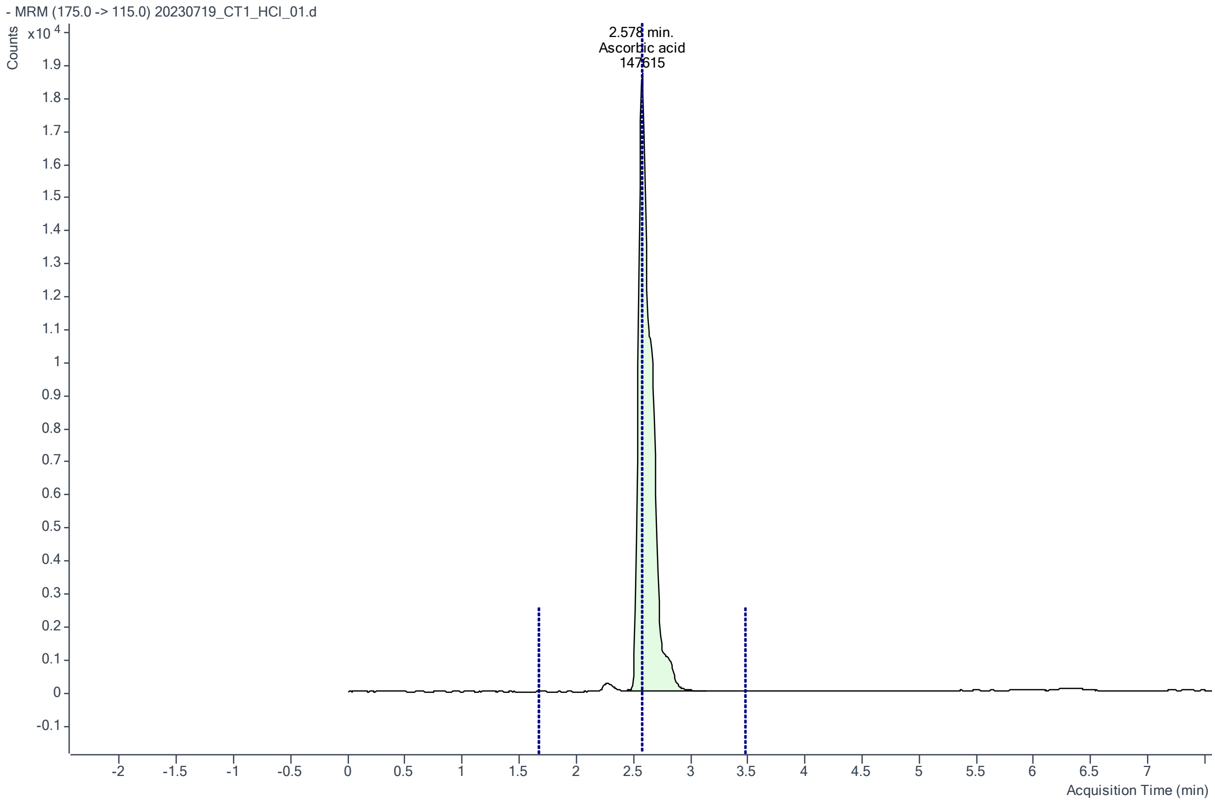
- MRM (175.0 -> 115.0) 20230719_CT1_HCl_01.d
Counts
x10 4
2.578 min.
Ascorbic acid
147615
Acquisition Time (min)

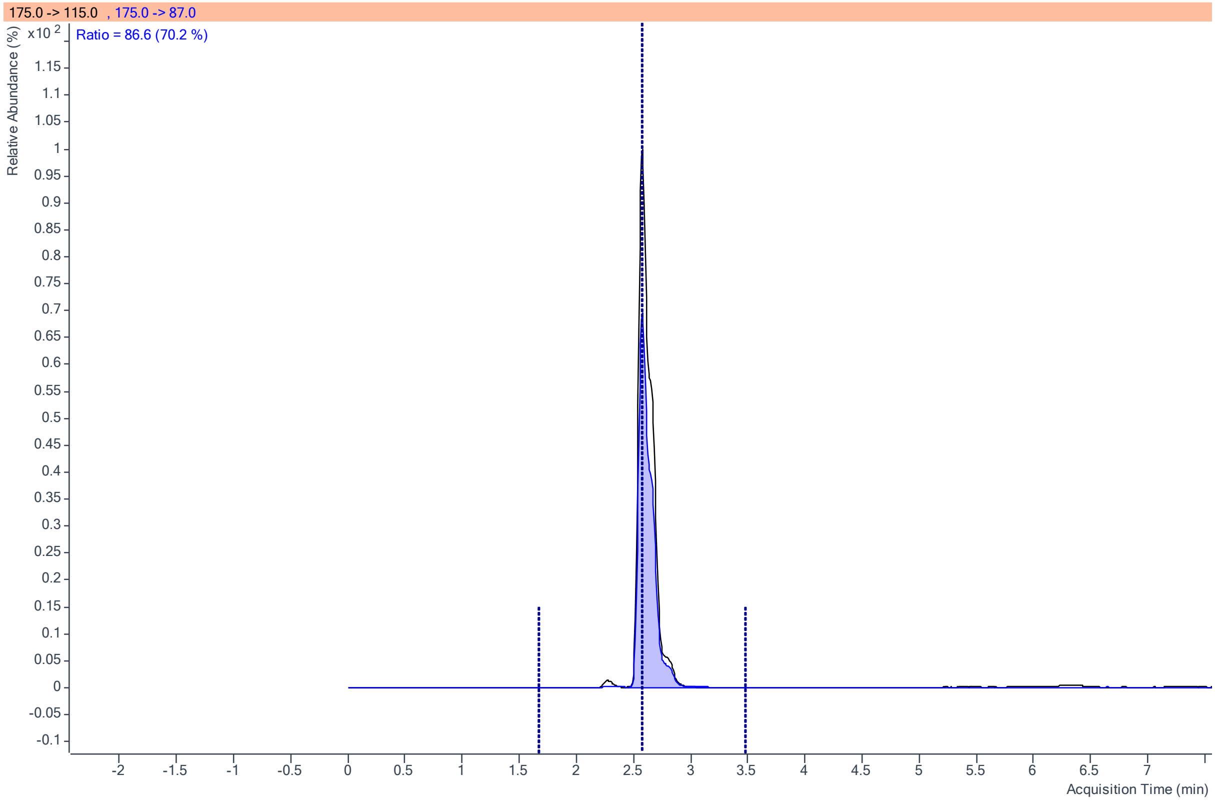
175.0 -> 115.0 , 175.0 -> 87.0
Relative Abundance (%)
x10 2
Ratio = 86.6 (70.2 %)
Acquisition Time (min)

(g)

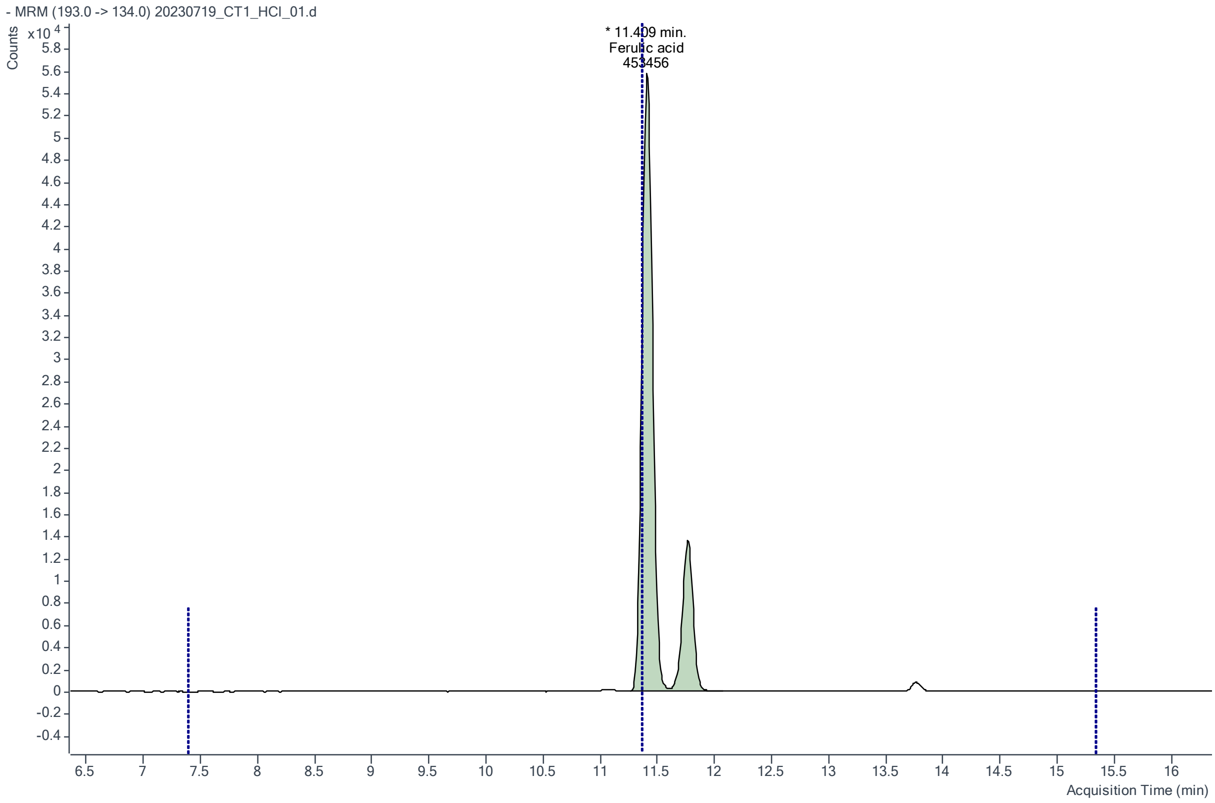
- MRM (193.0 -> 134.0) 20230719_CT1_HCl_01.d
Counts
x10 4
* 11.409 min.
Ferulic acid
453456
Acquisition Time (min)

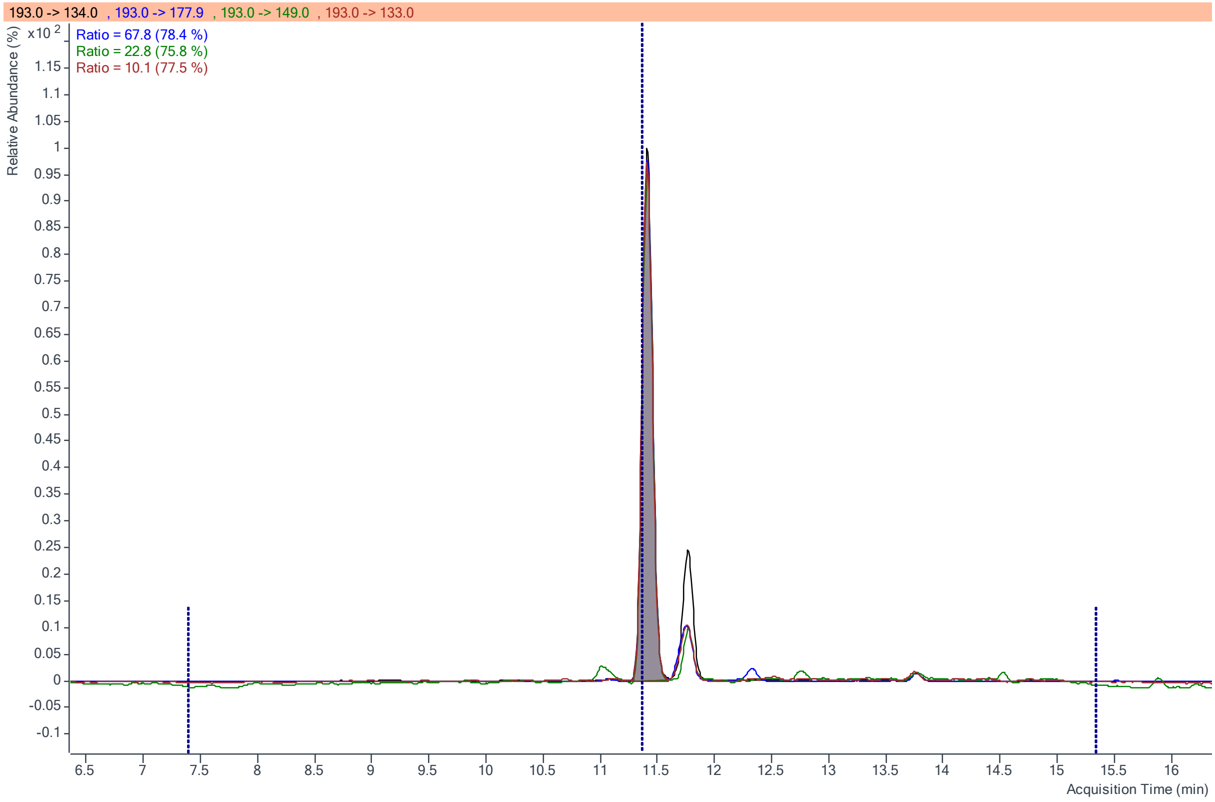
193.0 -> 134.0 , 193.0 -> 177.9 , 193.0 -> 149.0 , 193.0 -> 133.0
Relative Abundance (%)
x10 2
Ratio = 67.8 (78.4 %)
Ratio = 22.8 (75.8 %)
Ratio = 10.1 (77.5 %)
Acquisition Time (min)

(h)

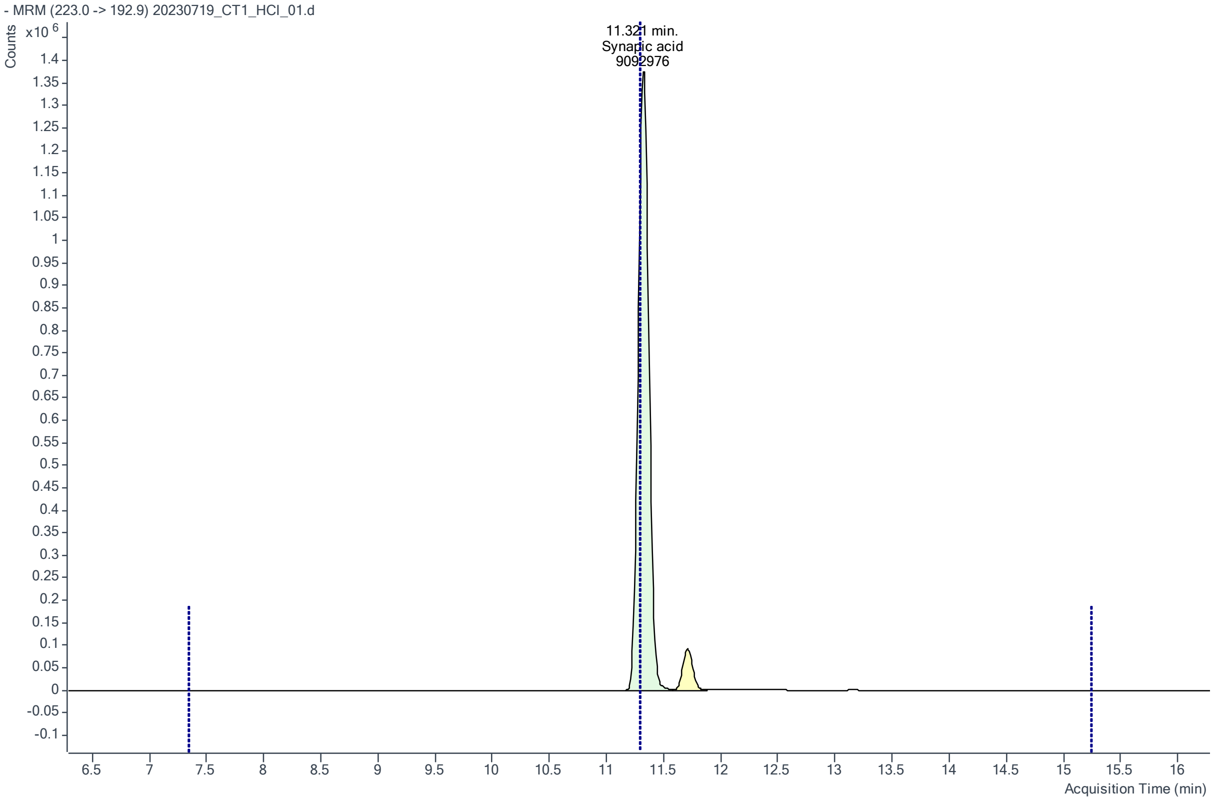
- MRM (223.0 -> 192.9) 20230719_CT1_HCl_01.d
Counts x10 6
11.321 min.
Synapic acid
9092976
Acquisition Time (min)

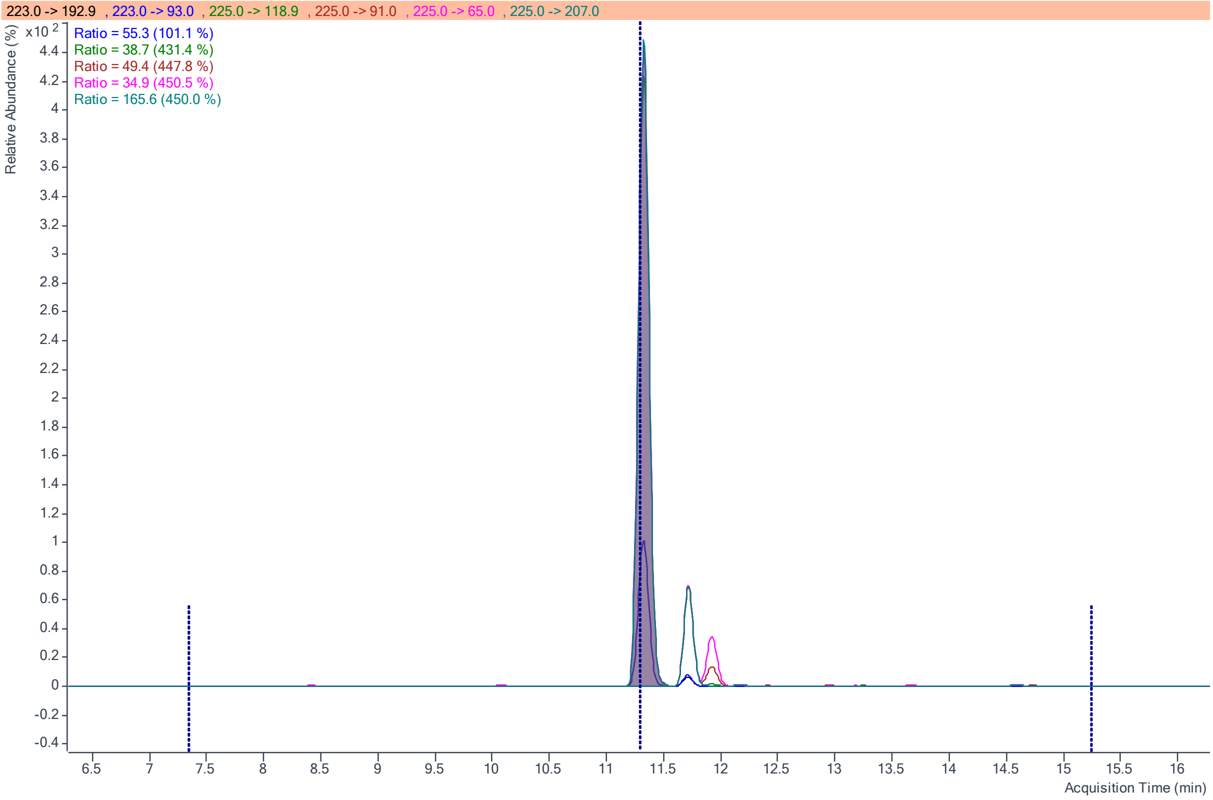
223.0 -> 192.9 , 223.0 -> 93.0 , 225.0 -> 118.9 , 225.0 -> 91.0 , 225.0 -> 65.0 , 225.0 -> 207.0
Relative Abundance (%) x10 2
Ratio = 55.3 (101.1 %)
Ratio = 38.7 (431.4 %)
Ratio = 49.4 (447.8 %)
Ratio = 34.9 (450.5 %)
Ratio = 165.6 (450.0 %)
Acquisition Time (min)

(i)

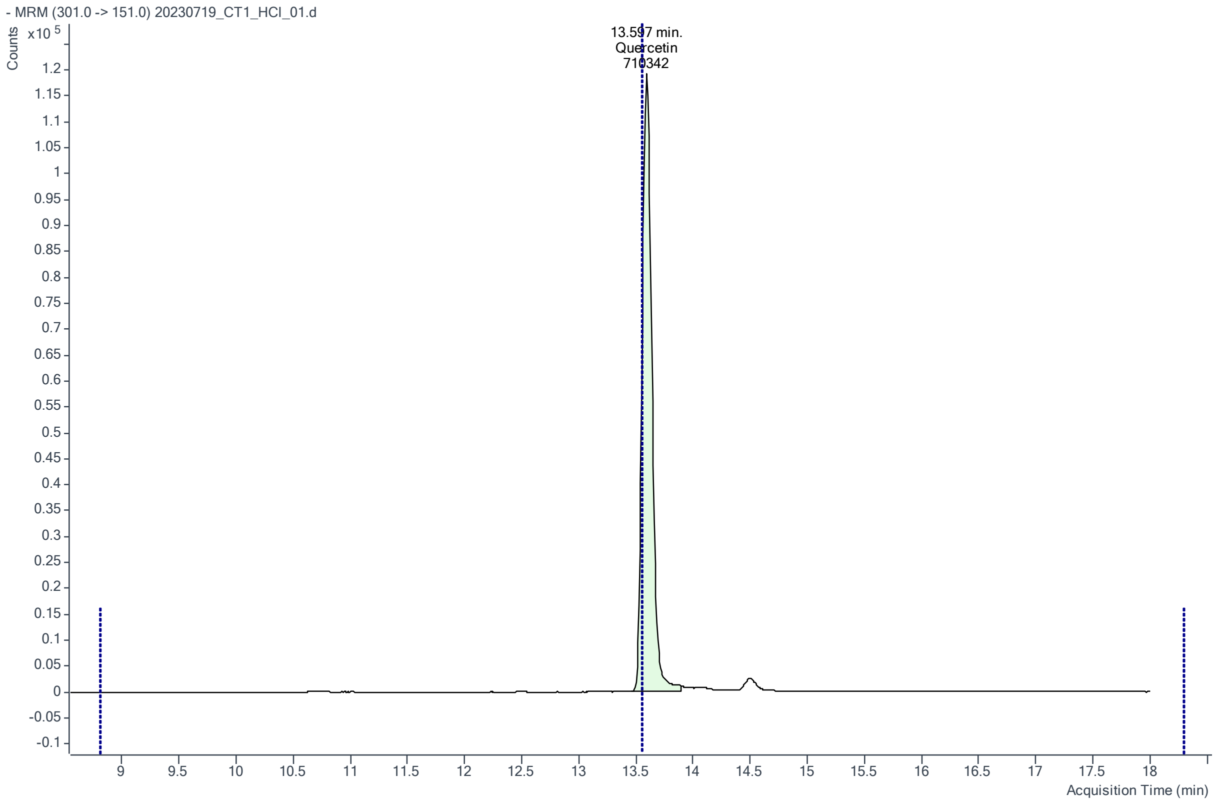
- MRM (301.0 -> 151.0) 20230719_CT1_HCl_01.d
Counts
x10 5
13.597 min.
Quercetin
710342
Acquisition Time (min)

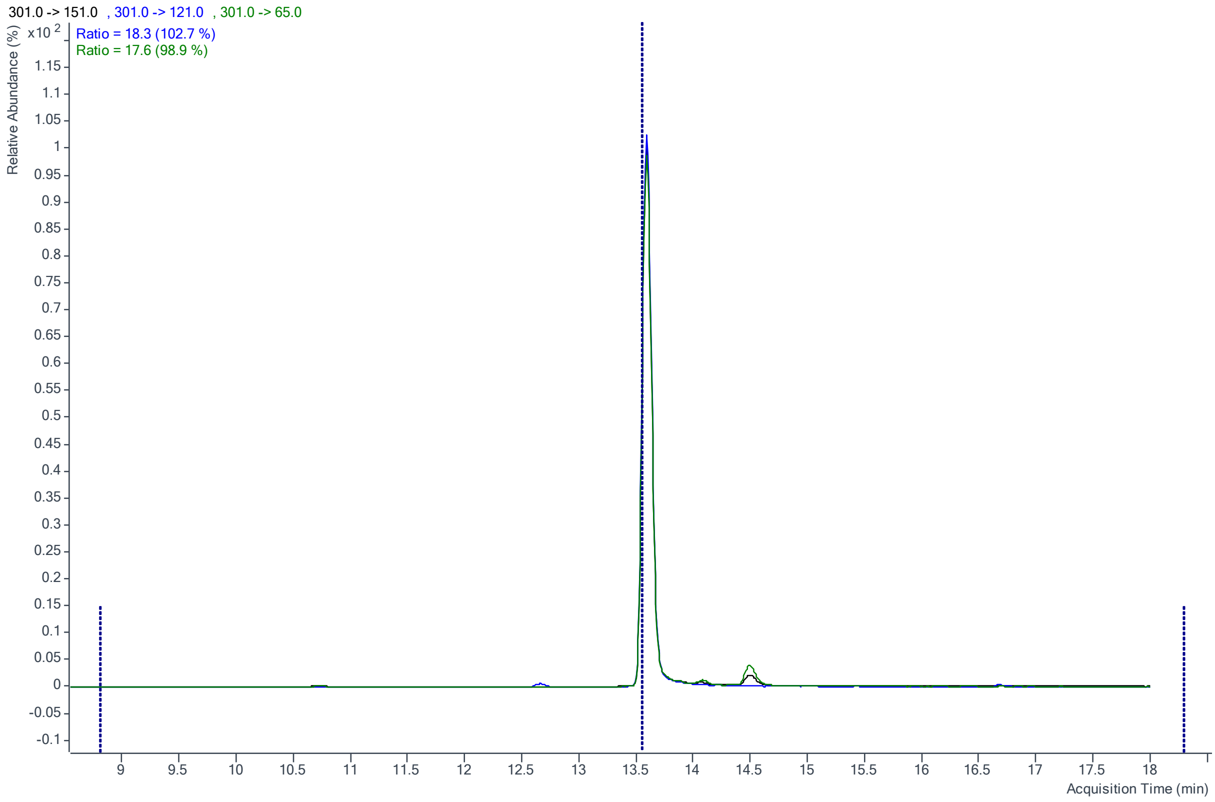
301.0 -> 151.0 , 301.0 -> 121.0 , 301.0 -> 65.0
Ratio = 18.3 (102.7 %)
Ratio = 17.6 (98.9 %)
Relative Abundance (%)
x10 2
Acquisition Time (min)

(j)

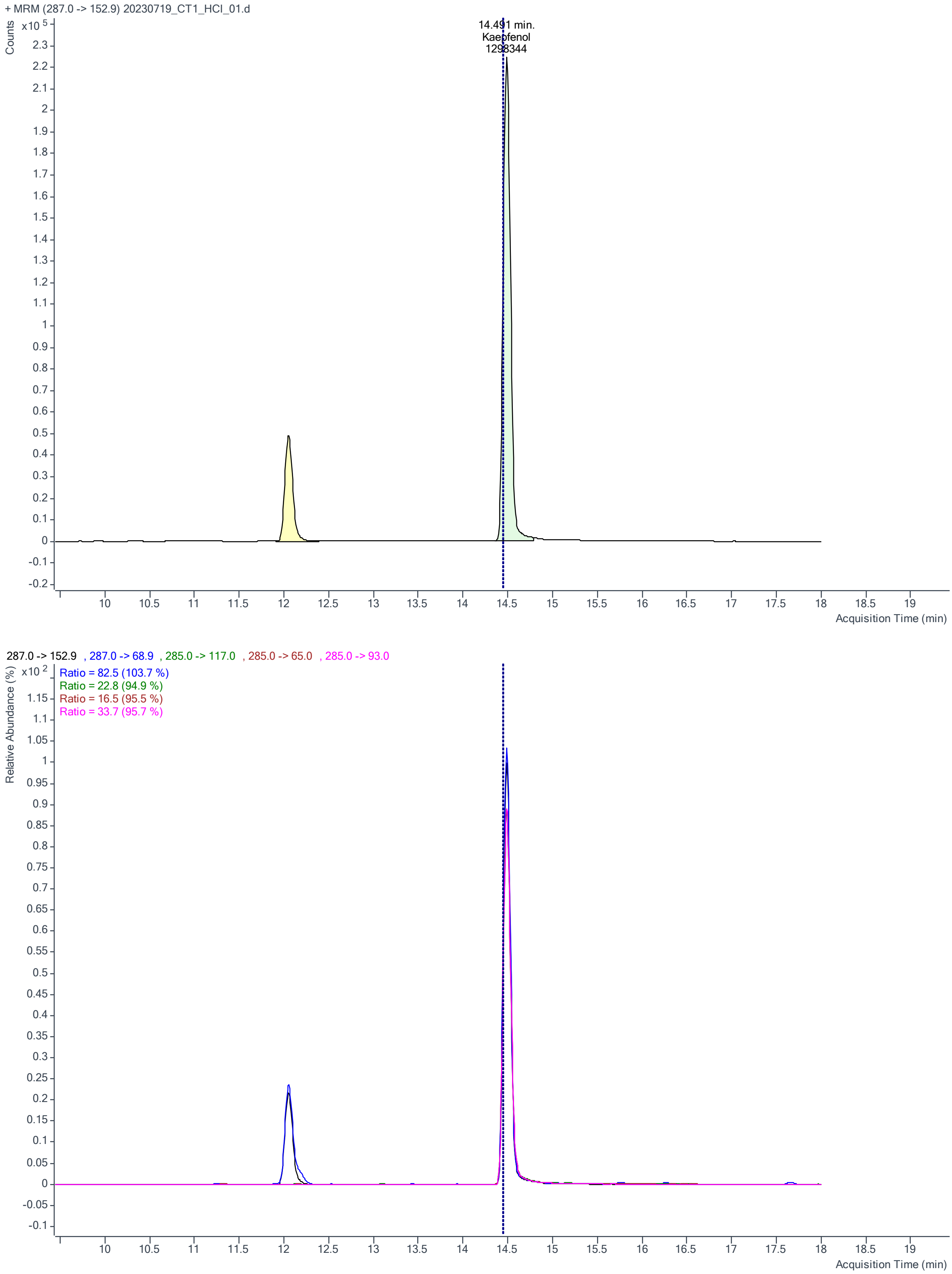

Figure S2. LC-MS/MS analysis of broccoli grown under low temperature. Nonhydrolyzed sample containing (a) *L*-ascorbic acid, (b) ferulic acid, (c) sinapic acid, (d) quercetin and (e) kaempferol. Hydrolyzed sample containing (f) *L*-ascorbic acid, (g) ferulic acid, (h) sinapic acid, (i) quercetin and (j) kaempferol.
