# Supplementary material for: Adjustments of the Phytochemical Profile of Broccoli to Low and High Growing Temperatures: Implications for the Bioactivity of Its Extracts
Source: Int J Mol Sci. 2024 Mar 26;25(7):3677. doi: 10.3390/ijms25073677 (PMC11011926; doi:10.3390/ijms25073677)
Supplement: Supplementary file 1 [file ijms-25-03677-s001.zip › Figure S3.pdf]

(a)

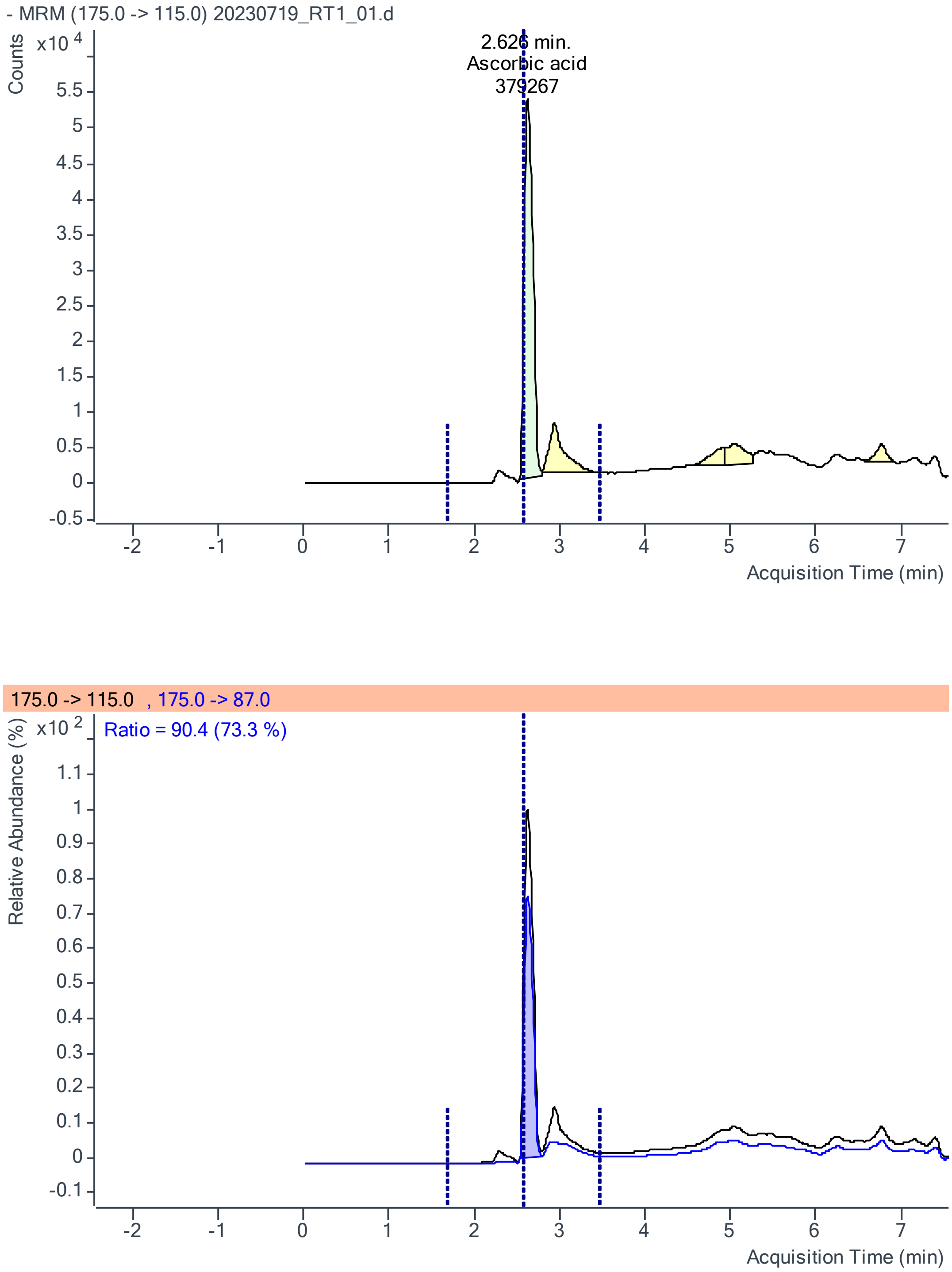
- MRM (175.0 -> 115.0) 20230719_RT1_01.d
Counts
x10 4
2.626 min.
Ascorbic acid
379267
5.5
5
4.5
4
3.5
3
2.5
2
1.5
1
0.5
0
-0.5
-2
-1
0
1
2
3
4
5
6
7
Acquisition Time (min)
175.0 -> 115.0
, 175.0 -> 87.0
x10 2
Ratio = 90.4 (73.3 %)
Relative Abundance (%)
1.1
1
0.9
0.8
0.7
0.6
0.5
0.4
0.3
0.2
0.1
0
-0.1
Acquisition Time (min)

(b)

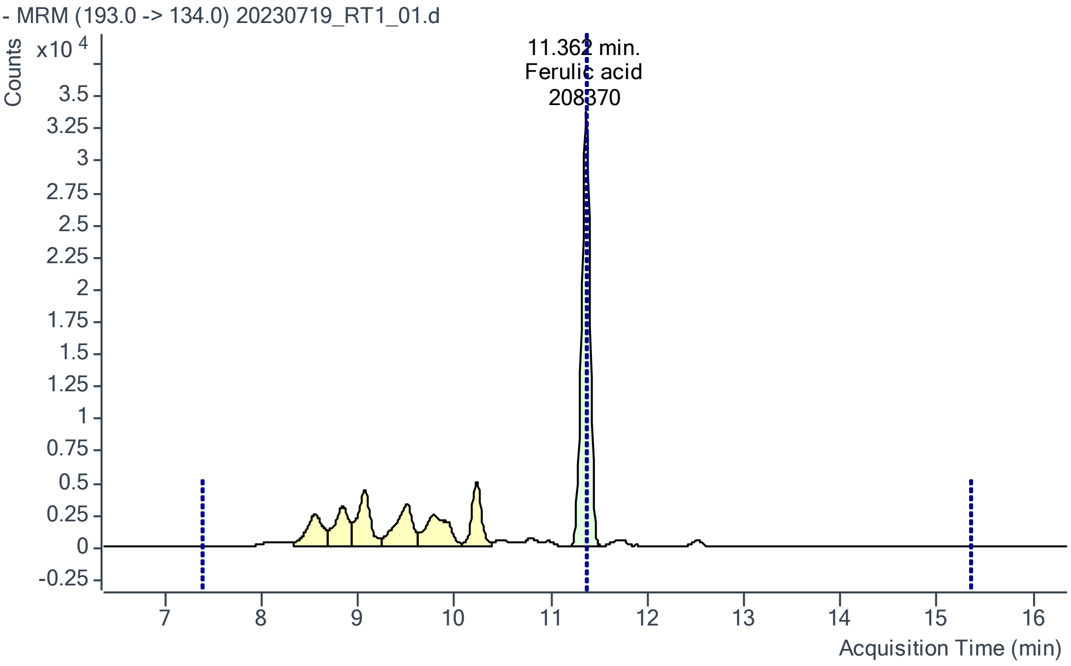

- MRM (193.0 -> 134.0) 20230719_RT1_01.d
Counts
x10 4
11.362 min.
Ferulic acid
208370
3.5
3.25
3
2.75
2.5
2.25
2
1.75
1.5
1.25
1
0.75
0.5
0.25
0
-0.25
7 8 9 10 11 12 13 14 15 16
Acquisition Time (min)

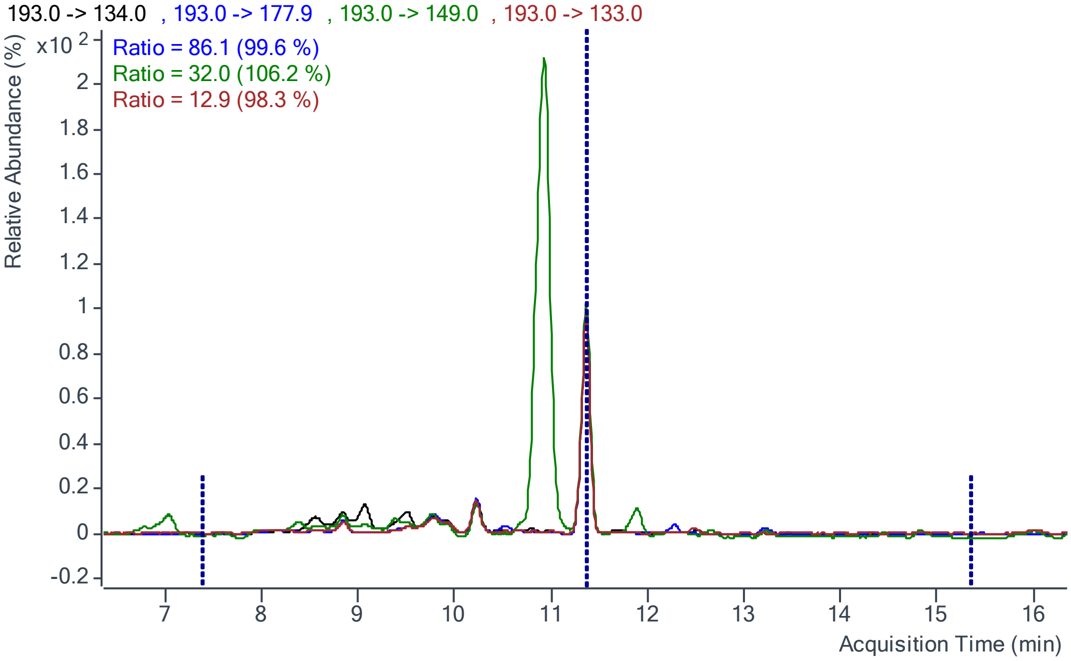

193.0 -> 134.0 , 193.0 -> 177.9 , 193.0 -> 149.0 , 193.0 -> 133.0
Relative Abundance (%)
x10 2
Ratio = 86.1 (99.6 %)
Ratio = 32.0 (106.2 %)
Ratio = 12.9 (98.3 %)
2
1.8
1.6
1.4
1.2
1
0.8
0.6
0.4
0.2
0
-0.2
7 8 9 10 11 12 13 14 15 16
Acquisition Time (min)

(c)

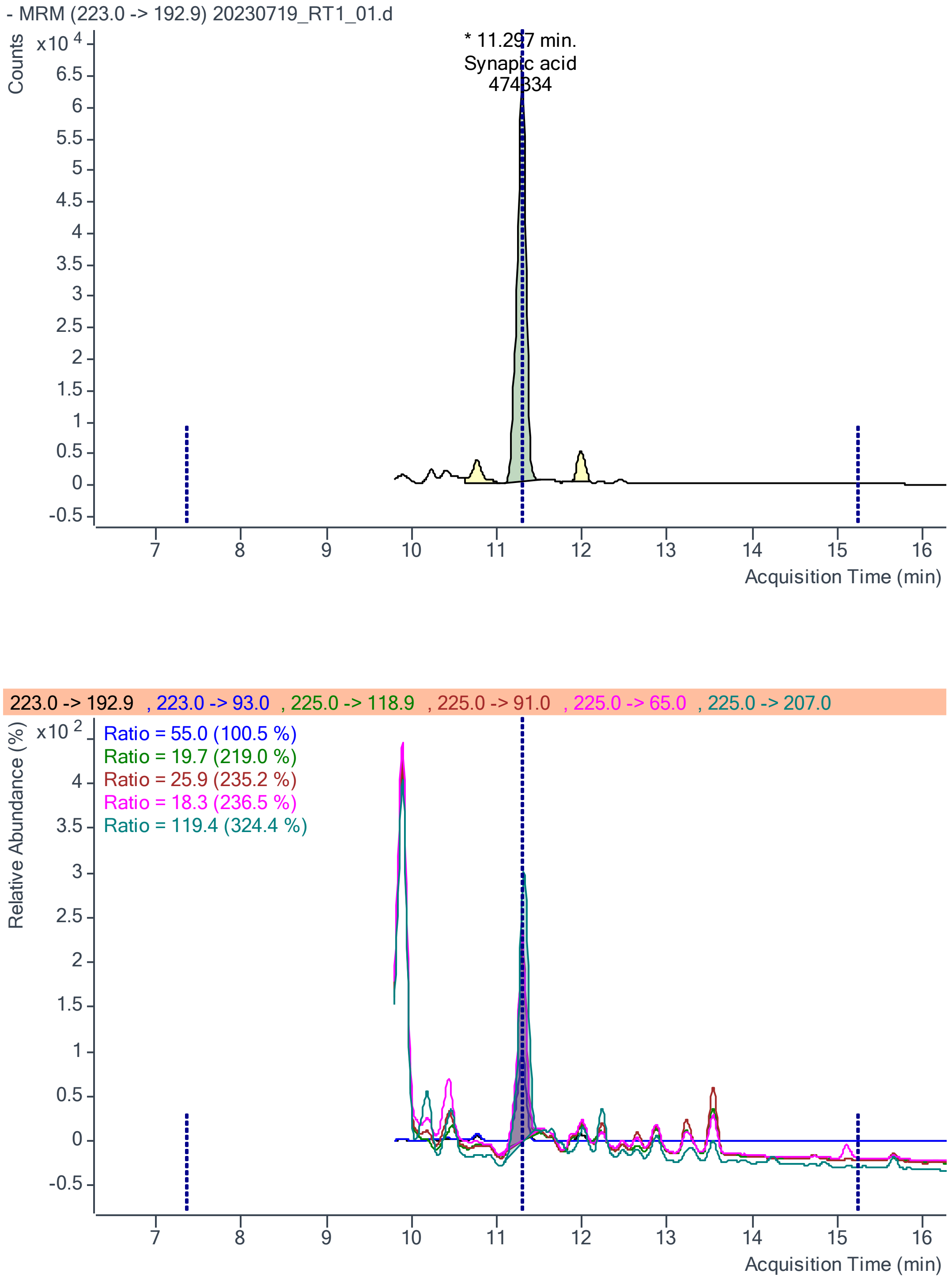

- MRM (223.0 -> 192.9) 20230719_RT1_01.d
* 11.297 min.
Synapic acid
474334
Counts
x10 4
Acquisition Time (min)
223.0 -> 192.9 , 223.0 -> 93.0 , 225.0 -> 118.9 , 225.0 -> 91.0 , 225.0 -> 65.0 , 225.0 -> 207.0
x10 2
Ratio = 55.0 (100.5 %)
Ratio = 19.7 (219.0 %)
Ratio = 25.9 (235.2 %)
Ratio = 18.3 (236.5 %)
Ratio = 119.4 (324.4 %)
Relative Abundance (%)
Acquisition Time (min)

(d)

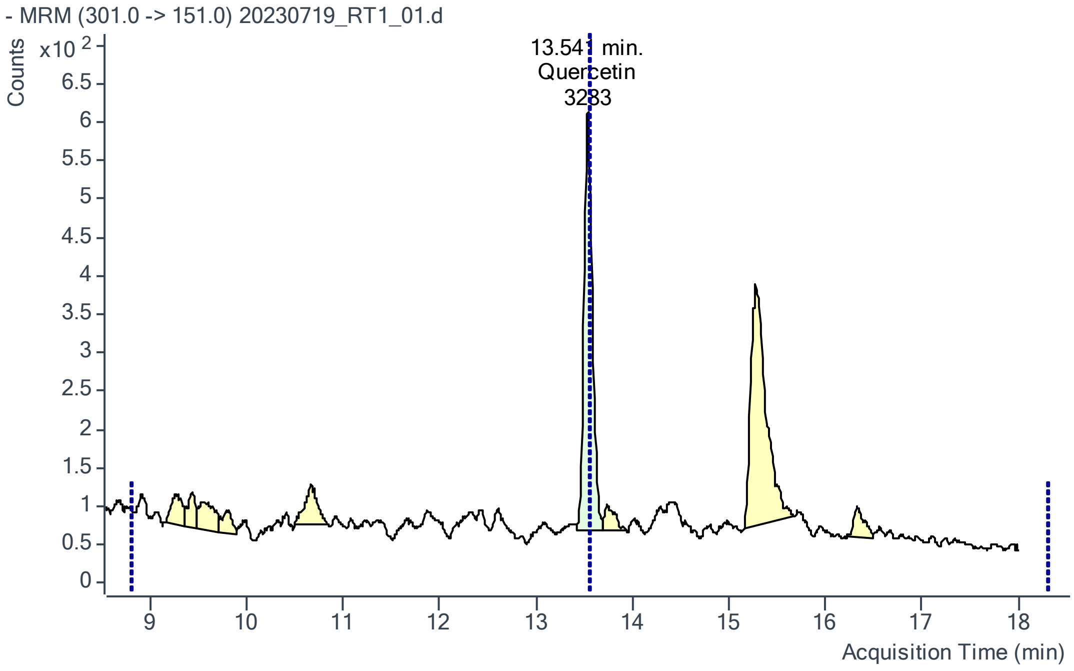
- MRM (301.0 -> 151.0) 20230719_RT1_01.d
Counts
x10 2
13.541 min.
Quercetin
3283
Acquisition Time (min)

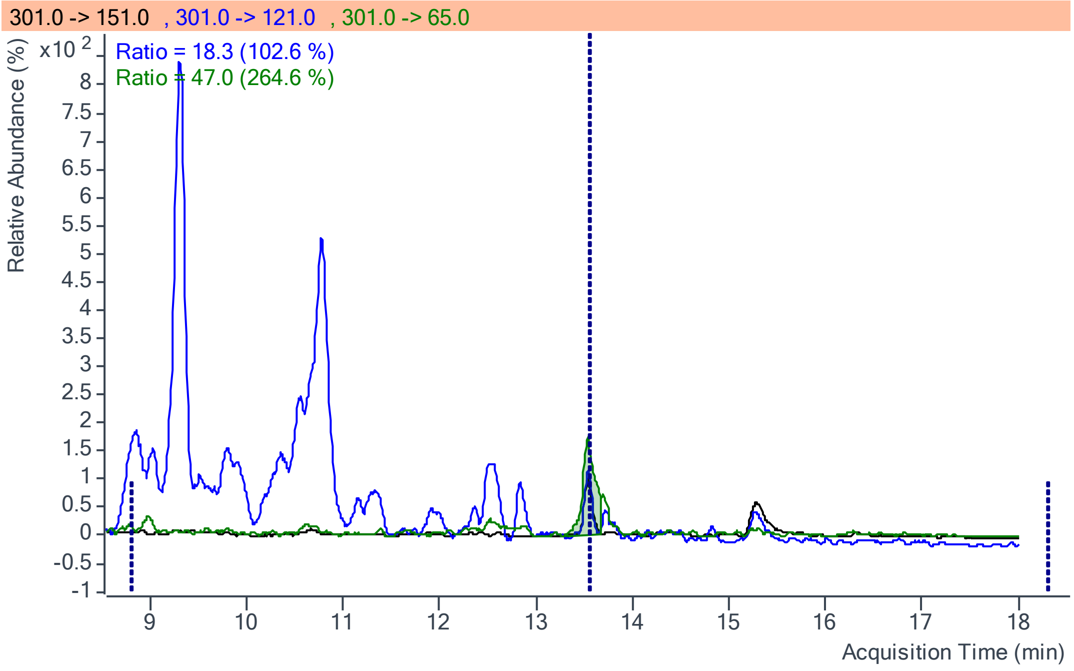
301.0 -> 151.0 , 301.0 -> 121.0 , 301.0 -> 65.0
Relative Abundance (%)
x10 2
Ratio = 18.3 (102.6 %)
Ratio = 47.0 (264.6 %)
Acquisition Time (min)

(e)

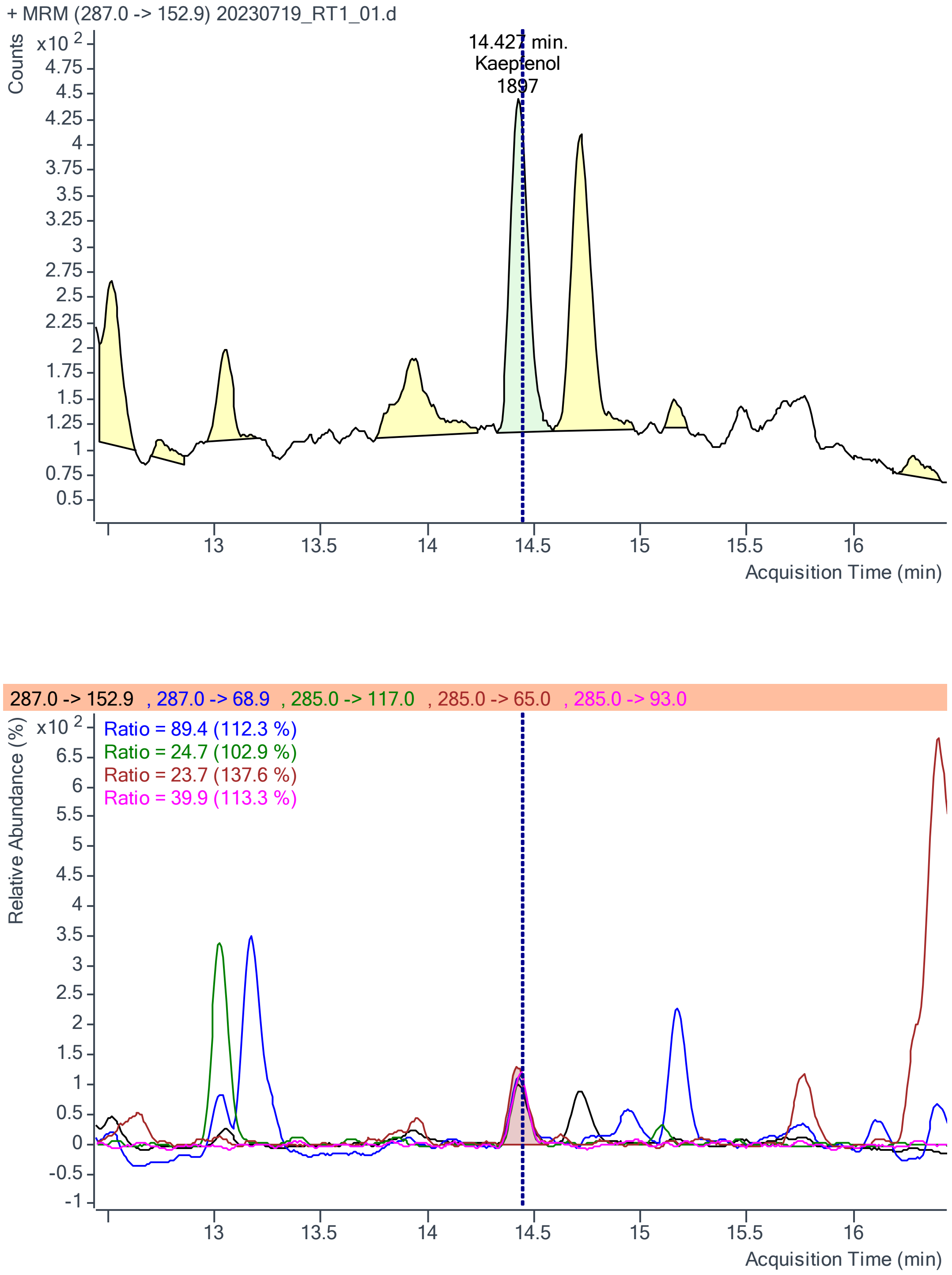
+ MRM (287.0 -> 152.9) 20230719_RT1_01.d
Counts
x10 2
14.427 min.
Kaeptenol
1897
Acquisition Time (min)
287.0 -> 152.9 , 287.0 -> 68.9 , 285.0 -> 117.0 , 285.0 -> 65.0 , 285.0 -> 93.0
Relative Abundance (%)
x10 2
Ratio = 89.4 (112.3 %)
Ratio = 24.7 (102.9 %)
Ratio = 23.7 (137.6 %)
Ratio = 39.9 (113.3 %)
Acquisition Time (min)

(f)

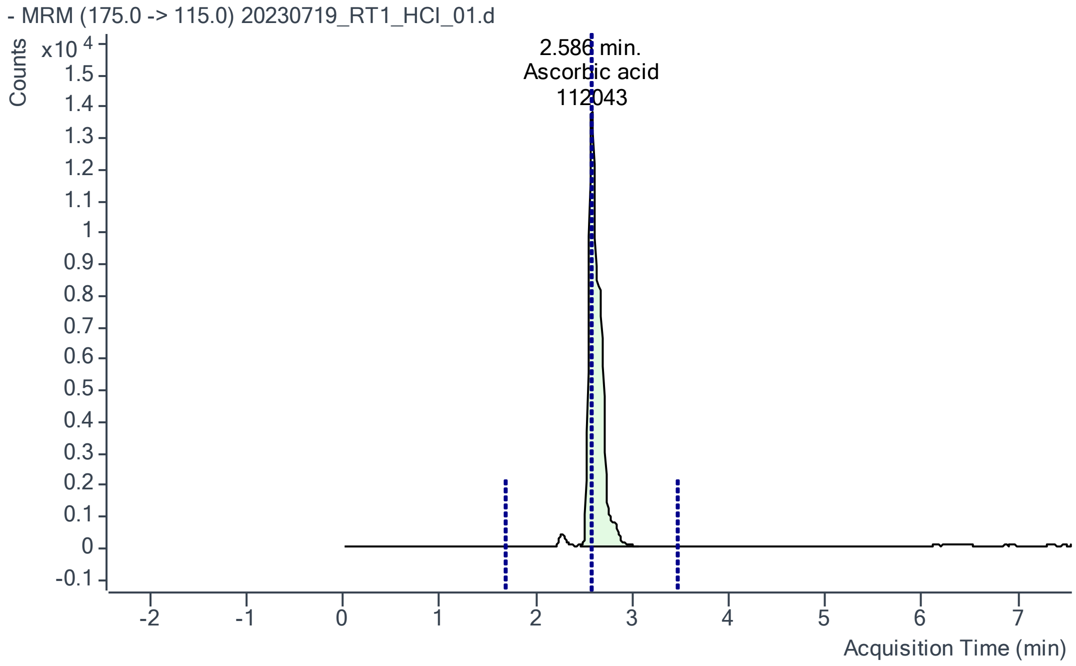
- MRM (175.0 -> 115.0) 20230719_RT1_HCl_01.d
Counts
x10 4
2.586 min.
Ascorbic acid
112043
Acquisition Time (min)

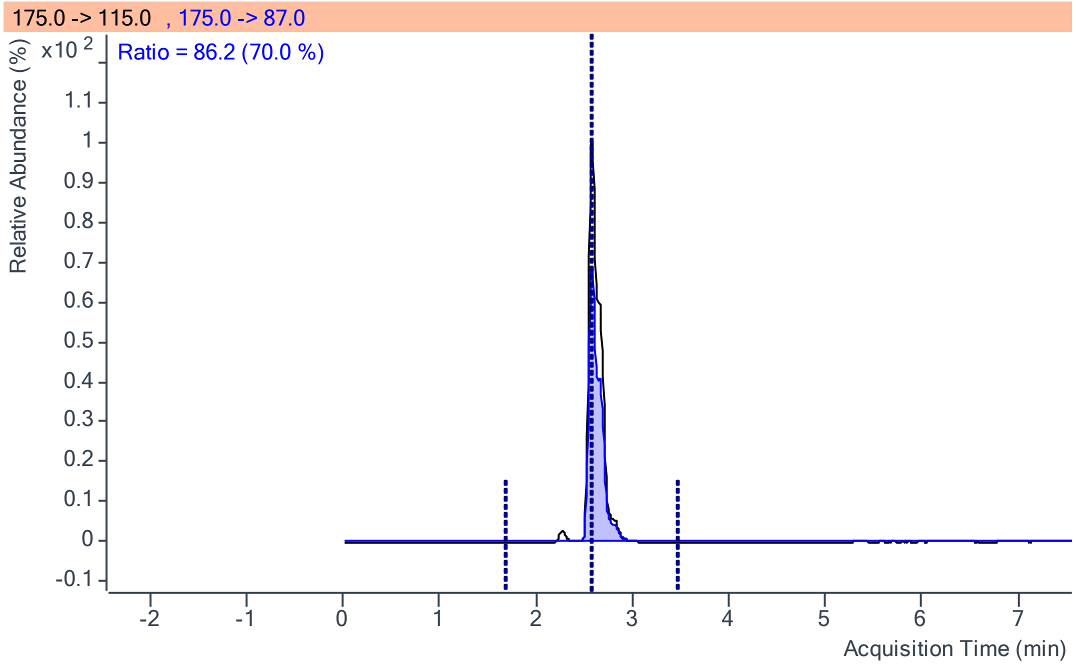
175.0 -> 115.0 , 175.0 -> 87.0
x10 2
Ratio = 86.2 (70.0 %)
Relative Abundance (%)
Acquisition Time (min)

(g)

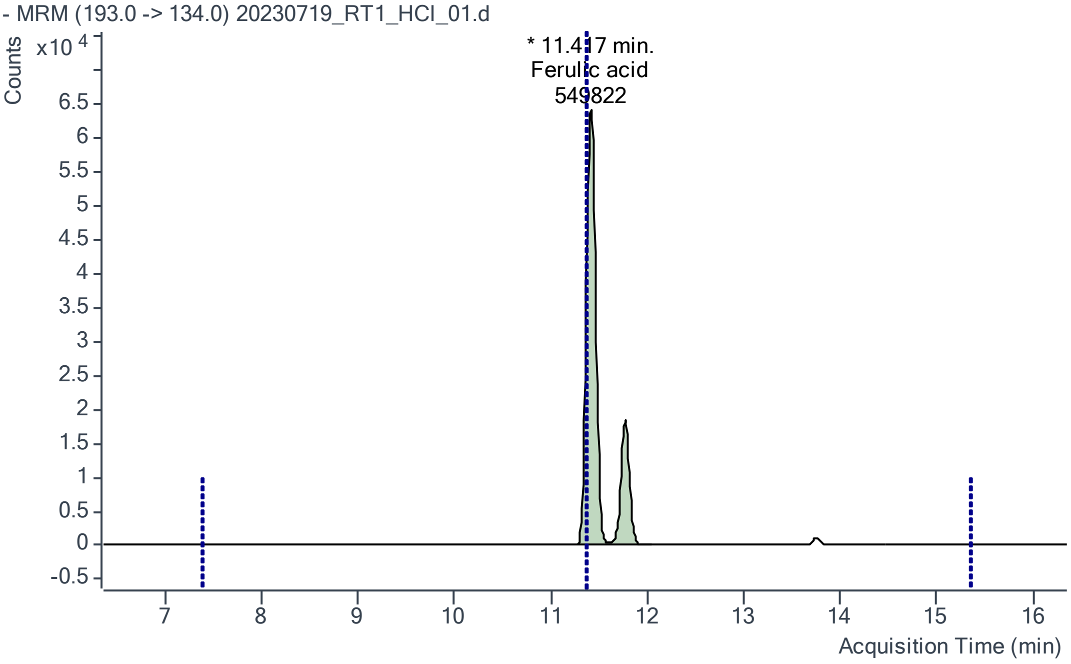
- MRM (193.0 -> 134.0) 20230719_RT1_HCl_01.d
Counts
x10 4
Ferulic acid
Acquisition Time (min)

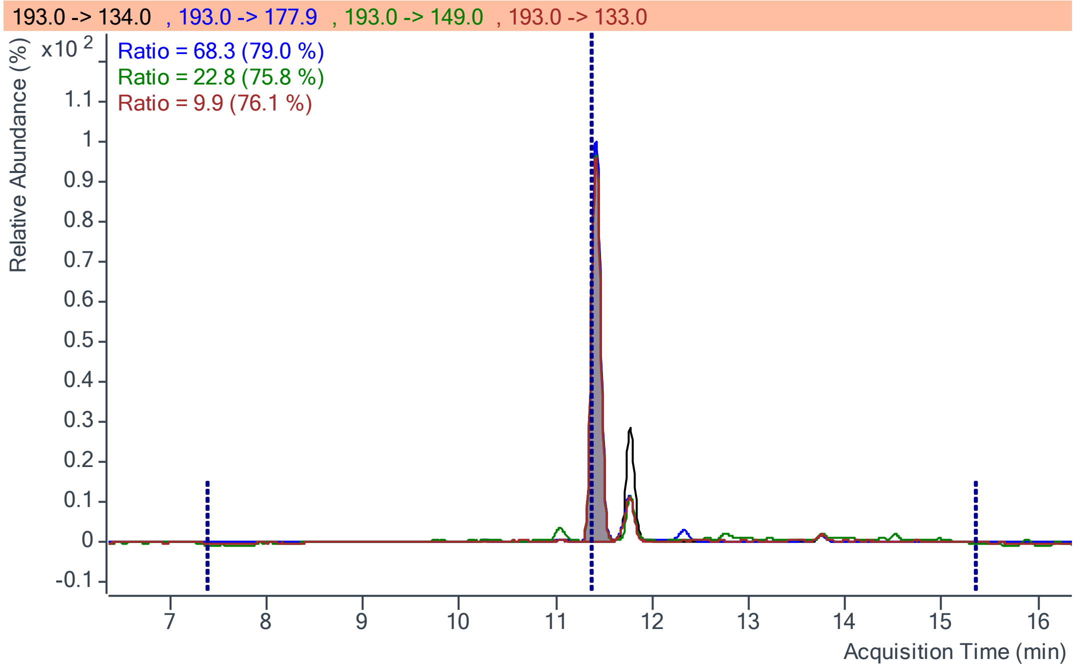
193.0 -> 134.0 , 193.0 -> 177.9 , 193.0 -> 149.0 , 193.0 -> 133.0
Relative Abundance (%)
x10 2
Ratio = 68.3 (79.0 %)
Ratio = 22.8 (75.8 %)
Ratio = 9.9 (76.1 %)
Acquisition Time (min)

(h)

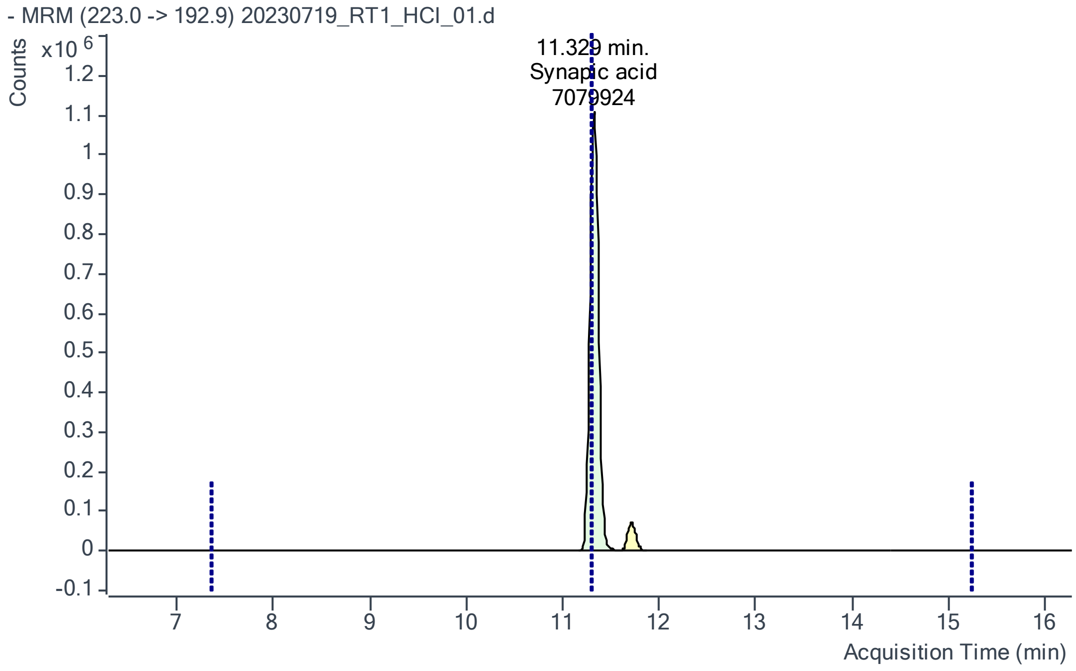
- MRM (223.0 -> 192.9) 20230719_RT1_HCl_01.d
Counts
x10 6
11.329 min.
Synapic acid
7079924
Acquisition Time (min)

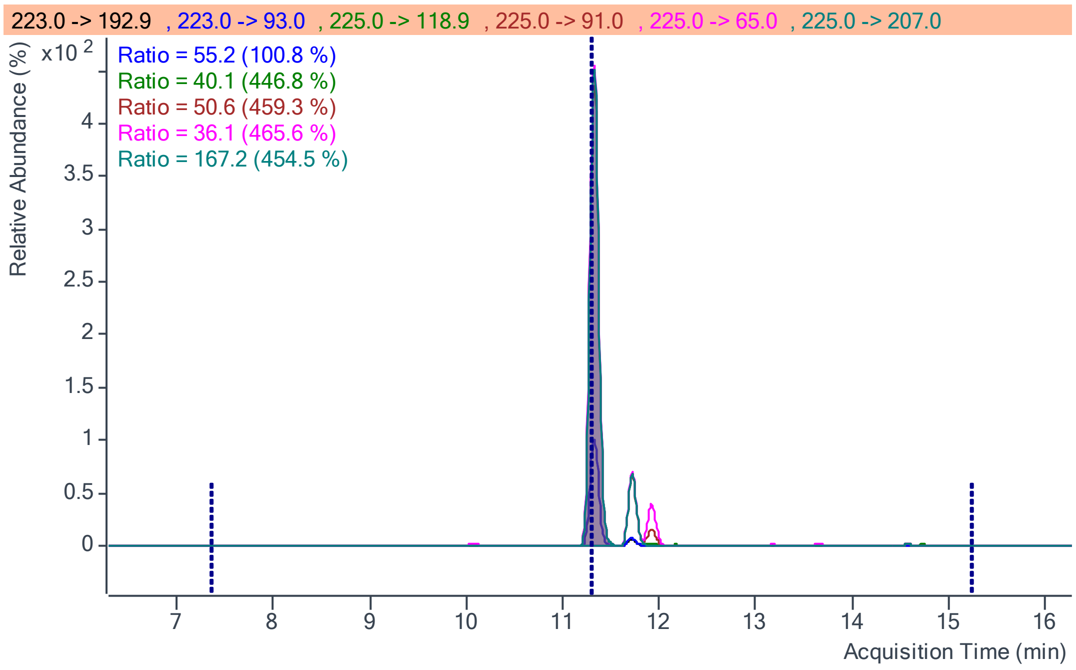
223.0 -> 192.9 , 223.0 -> 93.0 , 225.0 -> 118.9 , 225.0 -> 91.0 , 225.0 -> 65.0 , 225.0 -> 207.0
Relative Abundance (%)
x10 2
Ratio = 55.2 (100.8 %)
Ratio = 40.1 (446.8 %)
Ratio = 50.6 (459.3 %)
Ratio = 36.1 (465.6 %)
Ratio = 167.2 (454.5 %)
Acquisition Time (min)

(i)

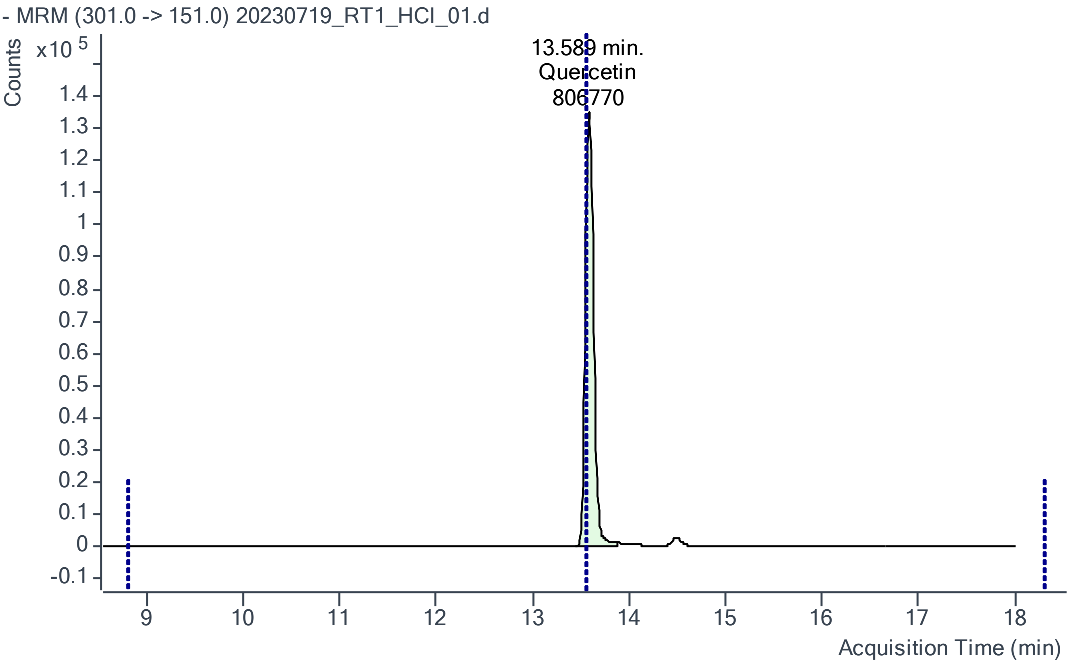
- MRM (301.0 -> 151.0) 20230719_RT1_HCl_01.d
Counts
x10 5
13.589 min.
Quercetin
806770
1.4
1.3
1.2
1.1
1
0.9
0.8
0.7
0.6
0.5
0.4
0.3
0.2
0.1
0
-0.1
9
10
11
12
13
14
15
16
17
18
Acquisition Time (min)

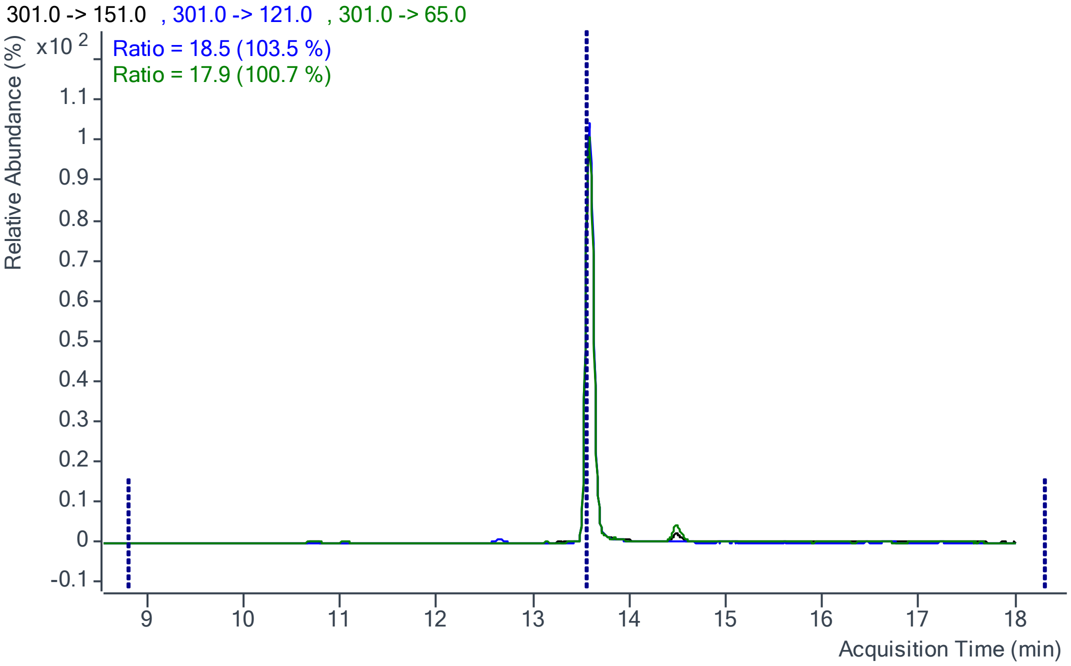
301.0 -> 151.0 , 301.0 -> 121.0 , 301.0 -> 65.0
x10 2
Ratio = 18.5 (103.5 %)
Ratio = 17.9 (100.7 %)
Relative Abundance (%)
1.1
1
0.9
0.8
0.7
0.6
0.5
0.4
0.3
0.2
0.1
0
-0.1
9
10
11
12
13
14
15
16
17
18
Acquisition Time (min)

(j)

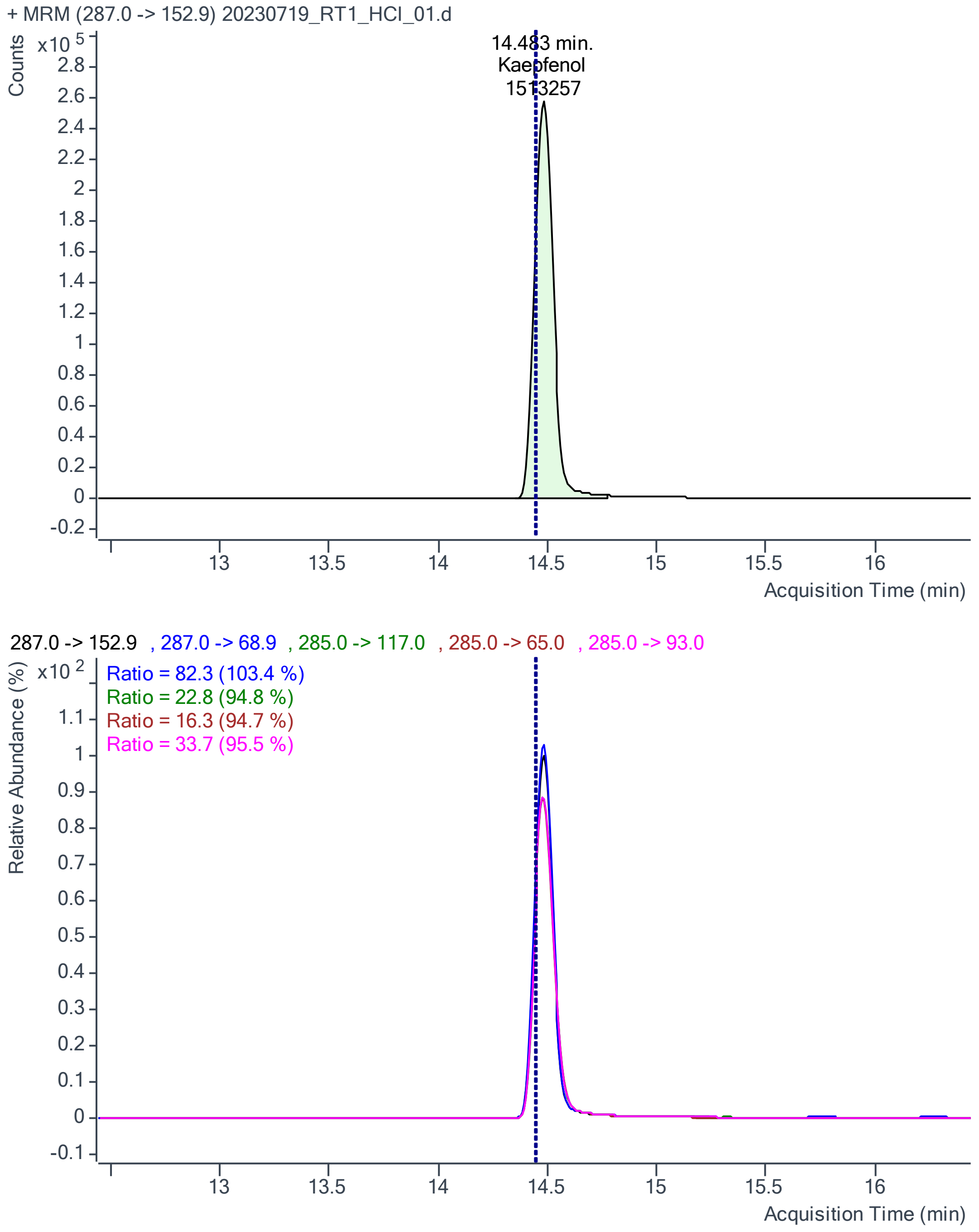

Figure S3. LC-MS/MS analysis of broccoli grown under room temperature. Nonhydrolyzed sample containing (a) *L*-ascorbic acid, (b) ferulic acid, (c) sinapic acid, (d) quercetin and (e) kaempferol. Hydrolyzed sample containing (f) *L*-ascorbic acid, (g) ferulic acid, (h) sinapic acid, (i) quercetin and (j) kaempferol.
