# Supplementary material for: Adjustments of the Phytochemical Profile of Broccoli to Low and High Growing Temperatures: Implications for the Bioactivity of Its Extracts
Source: Int J Mol Sci. 2024 Mar 26;25(7):3677. doi: 10.3390/ijms25073677 (PMC11011926; doi:10.3390/ijms25073677)
Supplement: Supplementary file 1 [file ijms-25-03677-s001.zip › Figure S4.pdf]

(a)

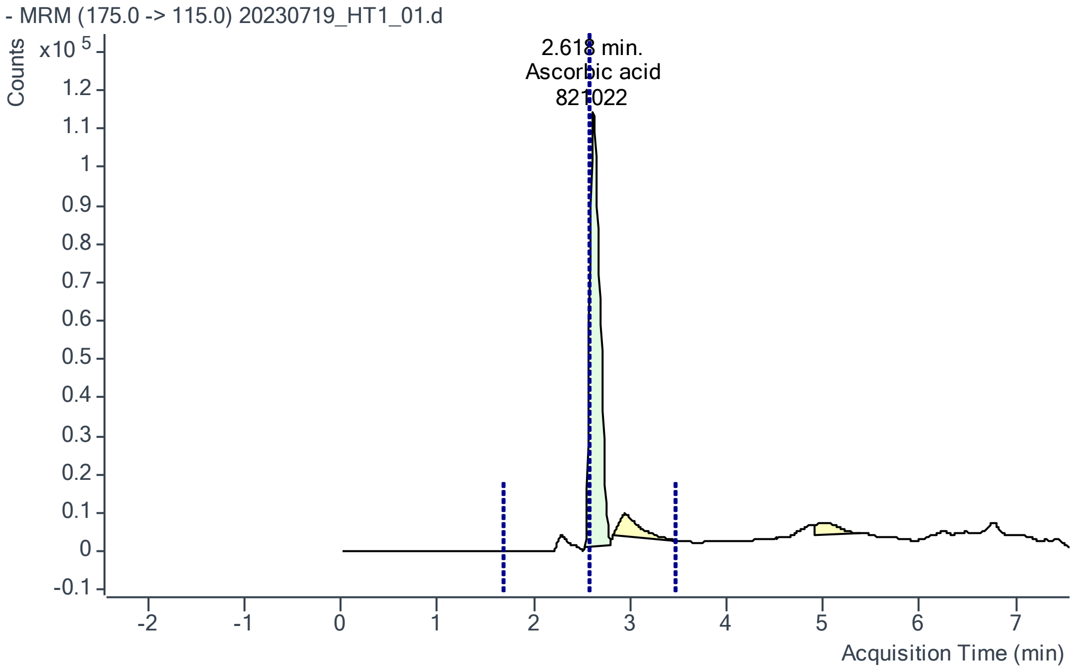
- MRM (175.0 -> 115.0) 20230719_HT1_01.d
Counts
x10 5
2.618 min.
Ascorbic acid
821022
1.2
1.1
1
0.9
0.8
0.7
0.6
0.5
0.4
0.3
0.2
0.1
0
-0.1
-2
-1
0
1
2
3
4
5
6
7
Acquisition Time (min)

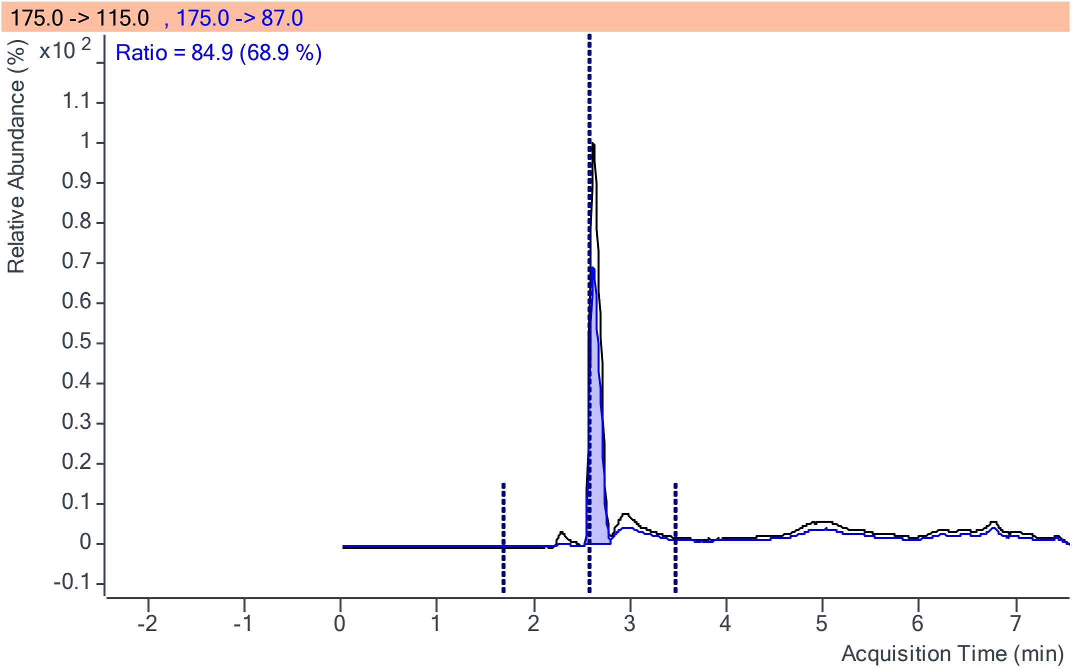
175.0 -> 115.0
, 175.0 -> 87.0
Relative Abundance (%)
x10 2
Ratio = 84.9 (68.9 %)
1.1
1
0.9
0.8
0.7
0.6
0.5
0.4
0.3
0.2
0.1
0
-0.1
-2
-1
0
1
2
3
4
5
6
7
Acquisition Time (min)

(b)

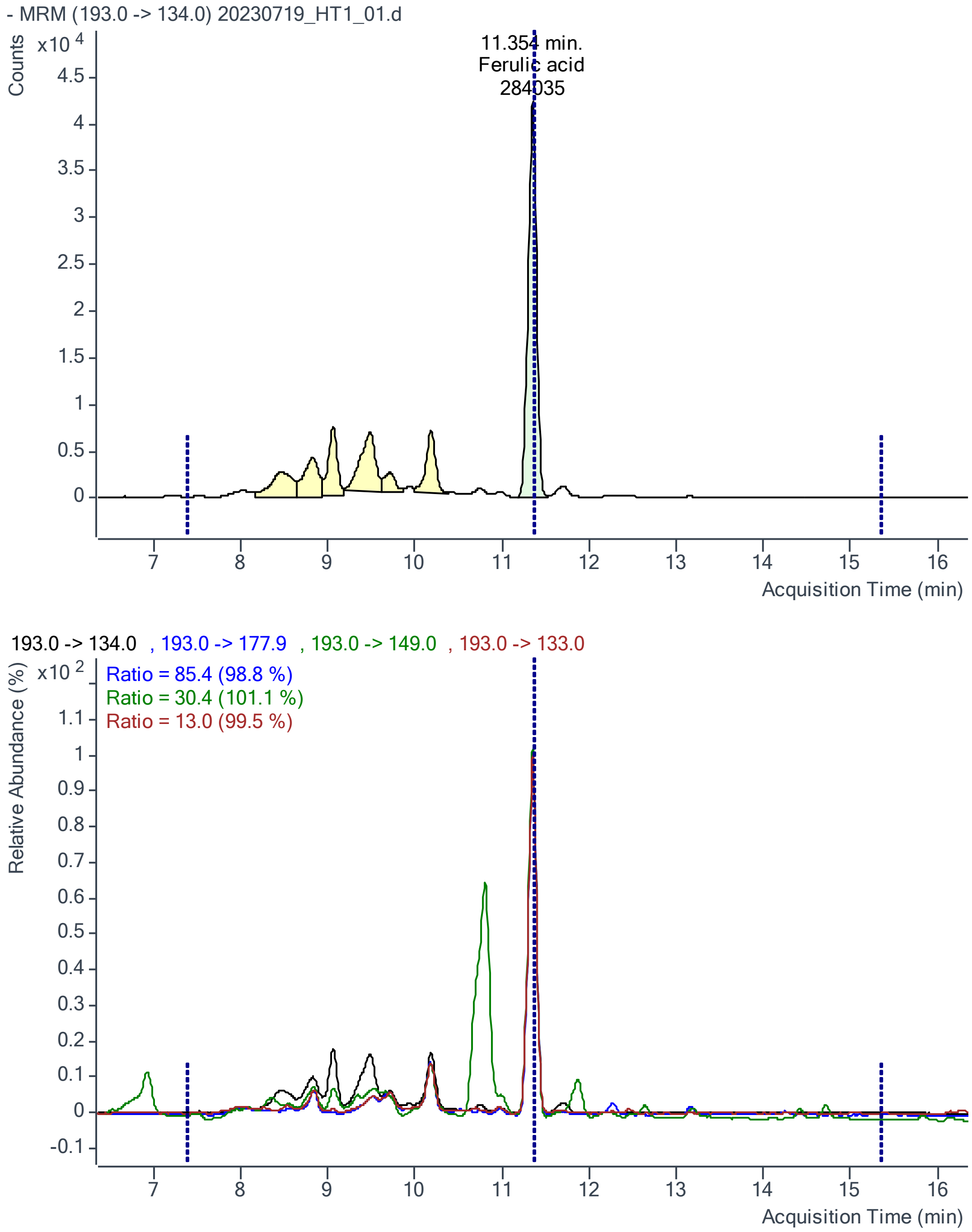
- MRM (193.0 -> 134.0) 20230719_HT1_01.d
Counts
x10 4
11.354 min.
Ferulic acid
284035
Acquisition Time (min)
193.0 -> 134.0 , 193.0 -> 177.9 , 193.0 -> 149.0 , 193.0 -> 133.0
Relative Abundance (%)
x10 2
Ratio = 85.4 (98.8 %)
Ratio = 30.4 (101.1 %)
Ratio = 13.0 (99.5 %)
Acquisition Time (min)

(c)

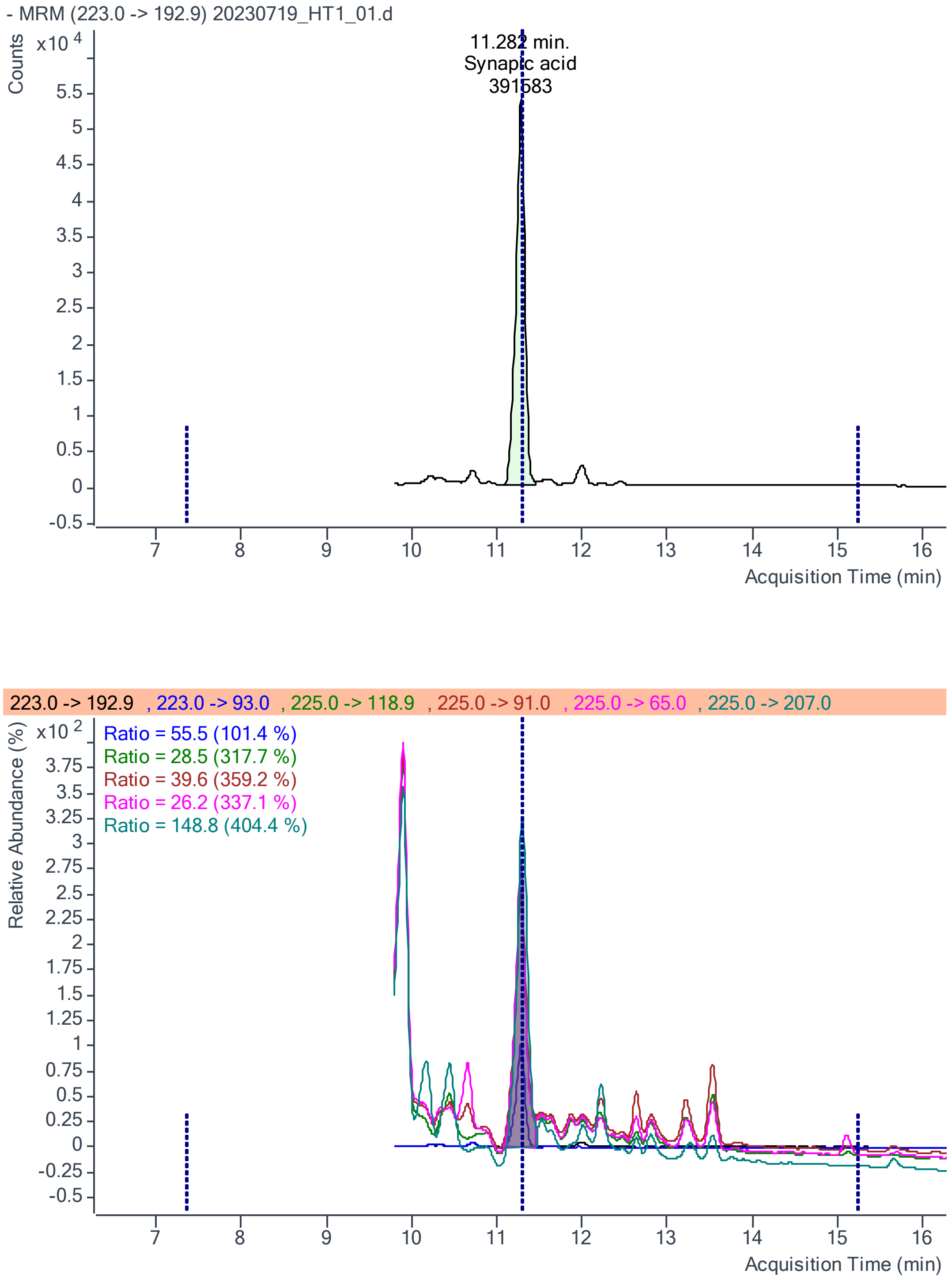
- MRM (223.0 -> 192.9) 20230719_HT1_01.d
Counts
x10 4
11.282 min.
Synaptic acid
391583
Acquisition Time (min)
223.0 -> 192.9 , 223.0 -> 93.0 , 225.0 -> 118.9 , 225.0 -> 91.0 , 225.0 -> 65.0 , 225.0 -> 207.0
x10 2
Relative Abundance (%)
Ratio = 55.5 (101.4 %)
Ratio = 28.5 (317.7 %)
Ratio = 39.6 (359.2 %)
Ratio = 26.2 (337.1 %)
Ratio = 148.8 (404.4 %)
Acquisition Time (min)

(d)

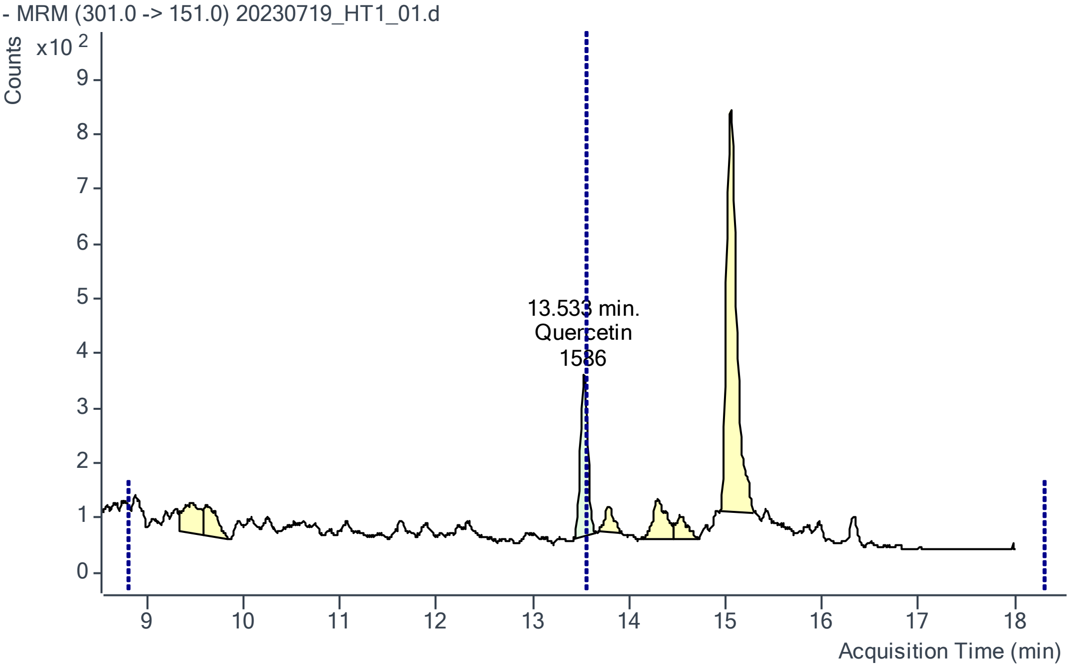
- MRM (301.0 -> 151.0) 20230719_HT1_01.d
x10 2
Counts
13.533 min.
Quercetin
1586
Acquisition Time (min)

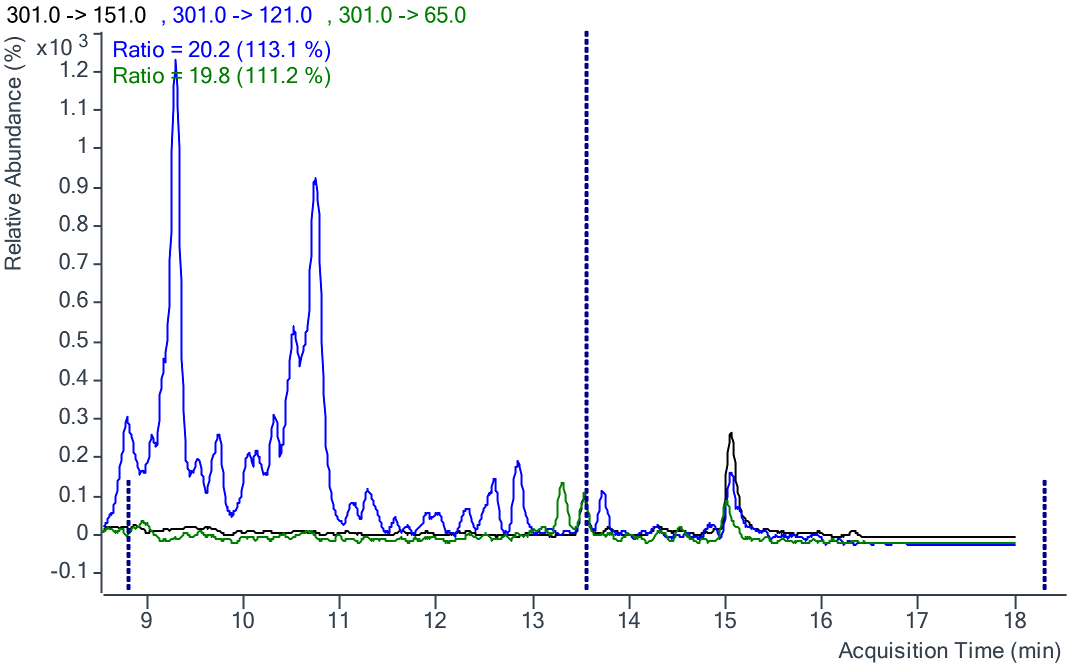
301.0 -> 151.0 , 301.0 -> 121.0 , 301.0 -> 65.0
x10 3
Ratio = 20.2 (113.1 %)
Ratio = 19.8 (111.2 %)
Relative Abundance (%)
Acquisition Time (min)

(e)

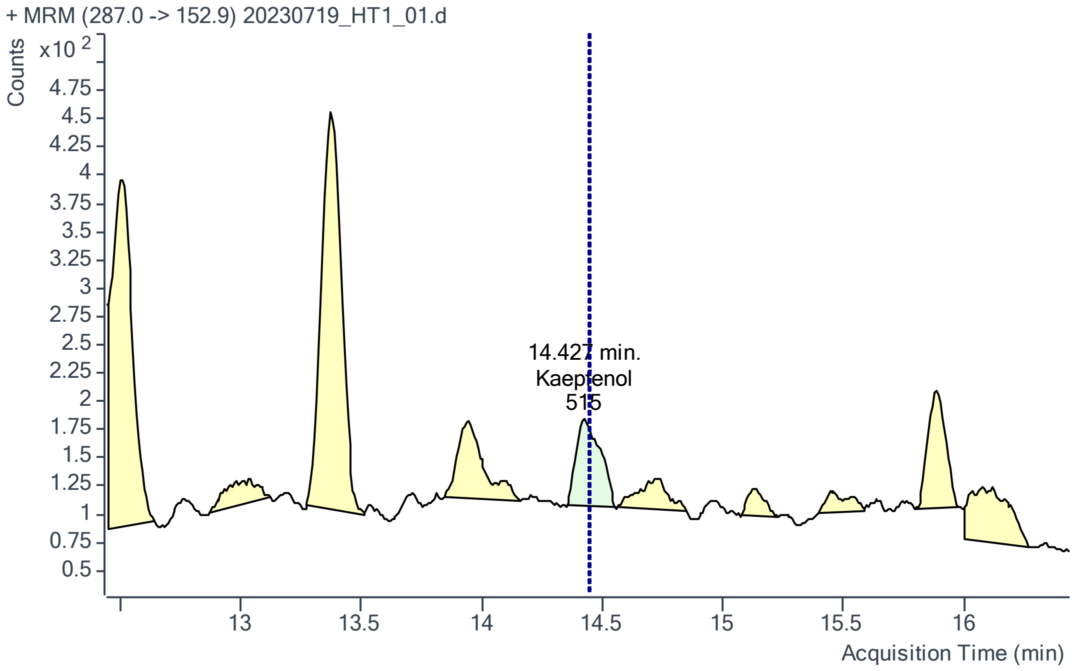
+ MRM (287.0 -> 152.9) 20230719_HT1_01.d
Counts
x10 2
4.75
4.5
4.25
4
3.75
3.5
3.25
3
2.75
2.5
2.25
2
1.75
1.5
1.25
1
0.75
0.5
14.427 min.
Kaepfenol
515
13
13.5
14
14.5
15
15.5
16
Acquisition Time (min)

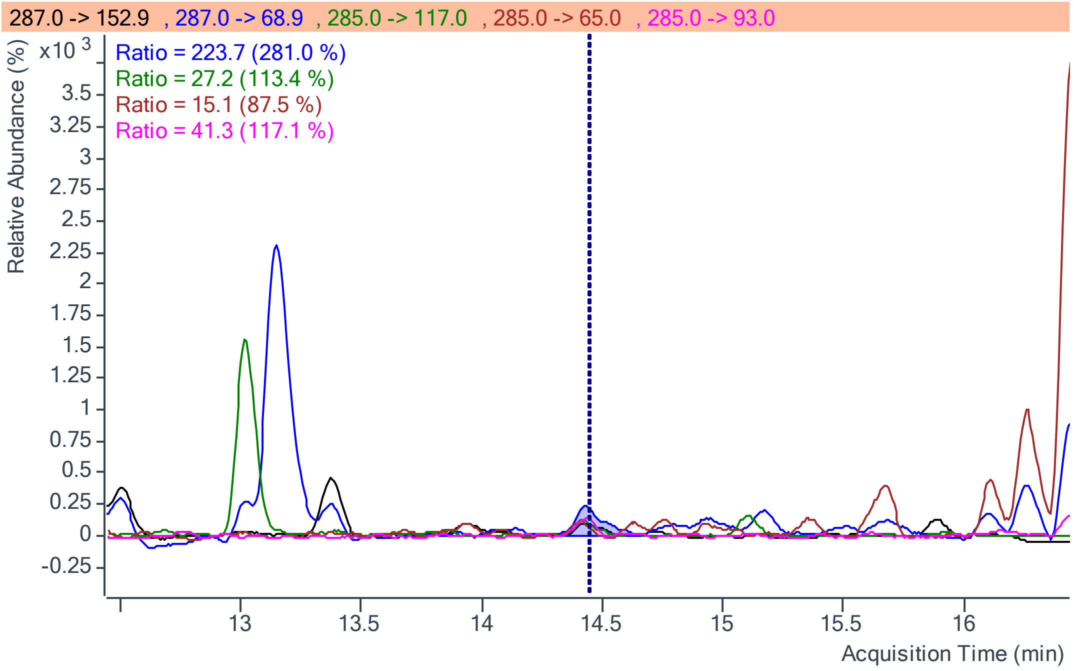
287.0 -> 152.9 , 287.0 -> 68.9 , 285.0 -> 117.0 , 285.0 -> 65.0 , 285.0 -> 93.0
Relative Abundance (%)
x10 3
Ratio = 223.7 (281.0 %)
Ratio = 27.2 (113.4 %)
Ratio = 15.1 (87.5 %)
Ratio = 41.3 (117.1 %)
3.5
3.25
3
2.75
2.5
2.25
2
1.75
1.5
1.25
1
0.75
0.5
0.25
0
-0.25
13
13.5
14
14.5
15
15.5
16
Acquisition Time (min)

(f)

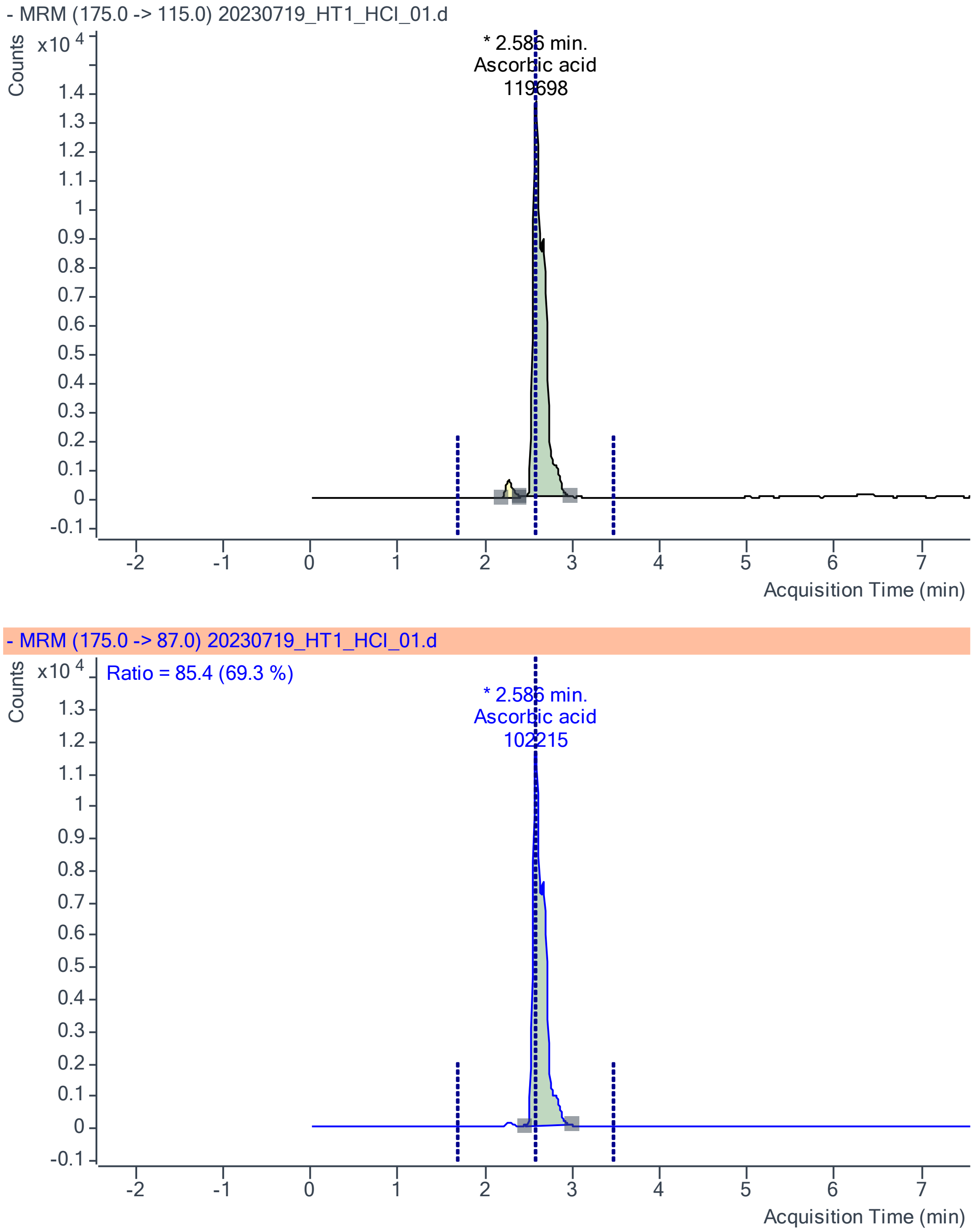
- MRM (175.0 -> 115.0) 20230719_HT1_HCl_01.d
Counts
x10 4
* 2.586 min.
Ascorbic acid
119698
1.4
1.3
1.2
1.1
1
0.9
0.8
0.7
0.6
0.5
0.4
0.3
0.2
0.1
0
-0.1
-2
-1
0
1
2
3
4
5
6
7
Acquisition Time (min)
- MRM (175.0 -> 87.0) 20230719_HT1_HCl_01.d
Counts
x10 4
Ratio = 85.4 (69.3 %)
* 2.586 min.
Ascorbic acid
102215
1.3
1.2
1.1
1
0.9
0.8
0.7
0.6
0.5
0.4
0.3
0.2
0.1
0
-0.1
-2
-1
0
1
2
3
4
5
6
7
Acquisition Time (min)

(g)

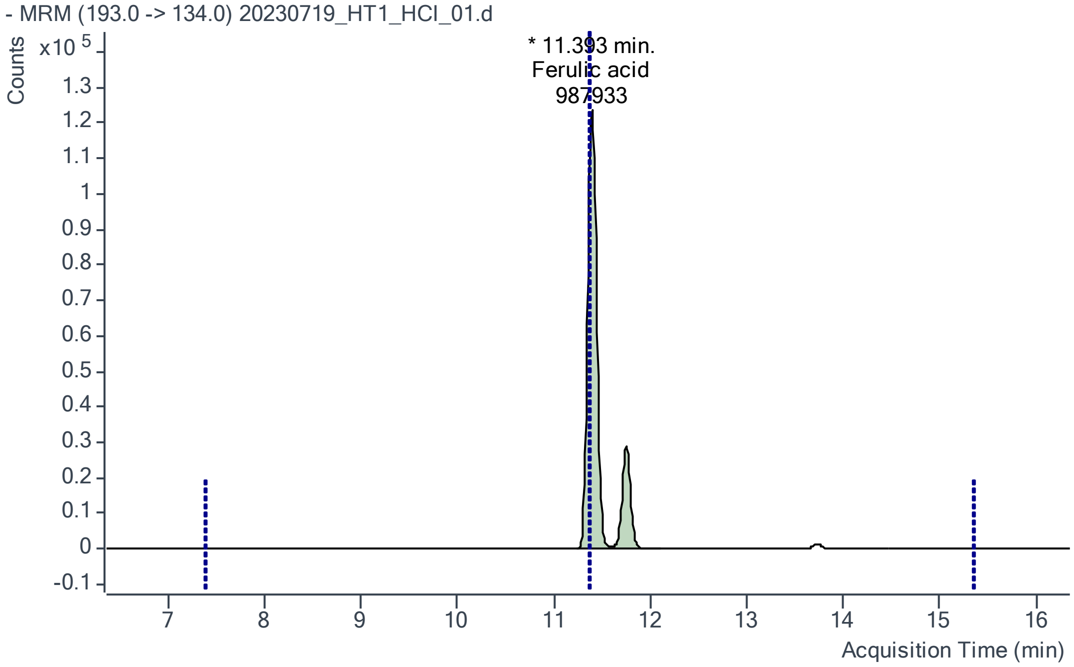
- MRM (193.0 -> 134.0) 20230719_HT1_HCl_01.d
Counts
x10 5
* 11.393 min.
Ferulic acid
987933
Acquisition Time (min)

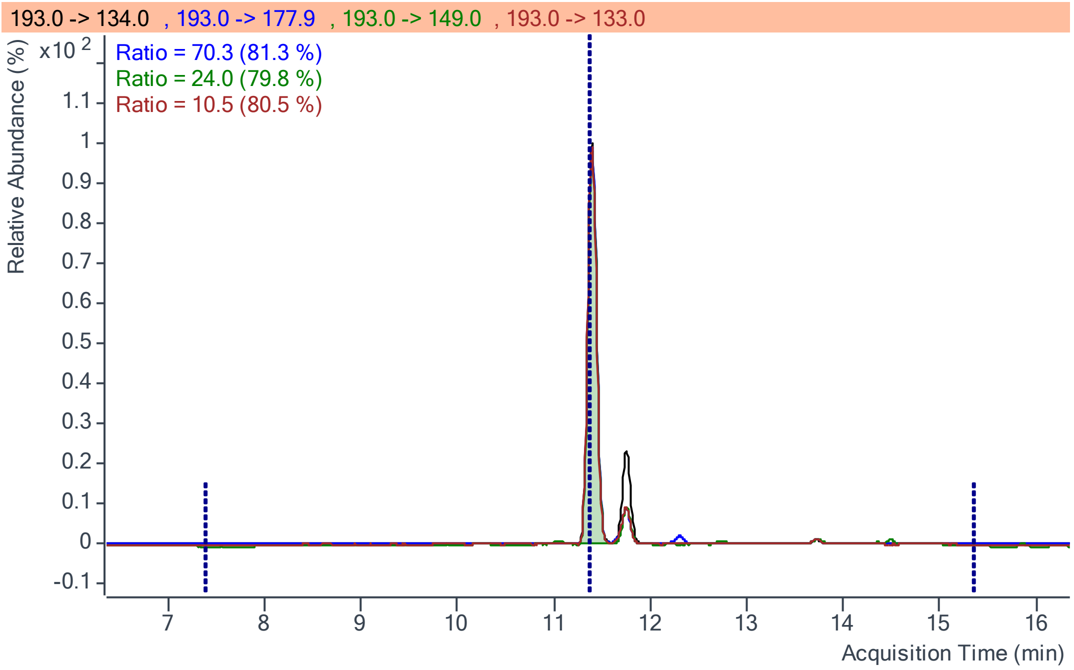
193.0 -> 134.0 , 193.0 -> 177.9 , 193.0 -> 149.0 , 193.0 -> 133.0
x10 2
Ratio = 70.3 (81.3 %)
Ratio = 24.0 (79.8 %)
Ratio = 10.5 (80.5 %)
Relative Abundance (%)
Acquisition Time (min)

(h)

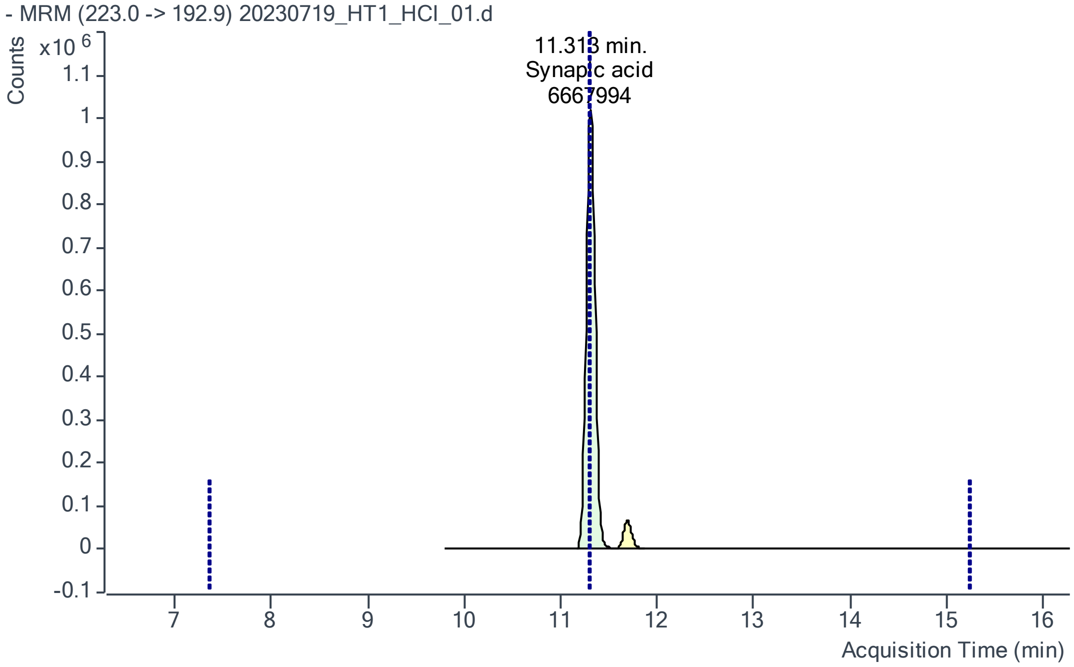
- MRM (223.0 -> 192.9) 20230719_HT1_HCl_01.d
Counts
x10 6
11.313 min.
Synapic acid
6667994
Acquisition Time (min)

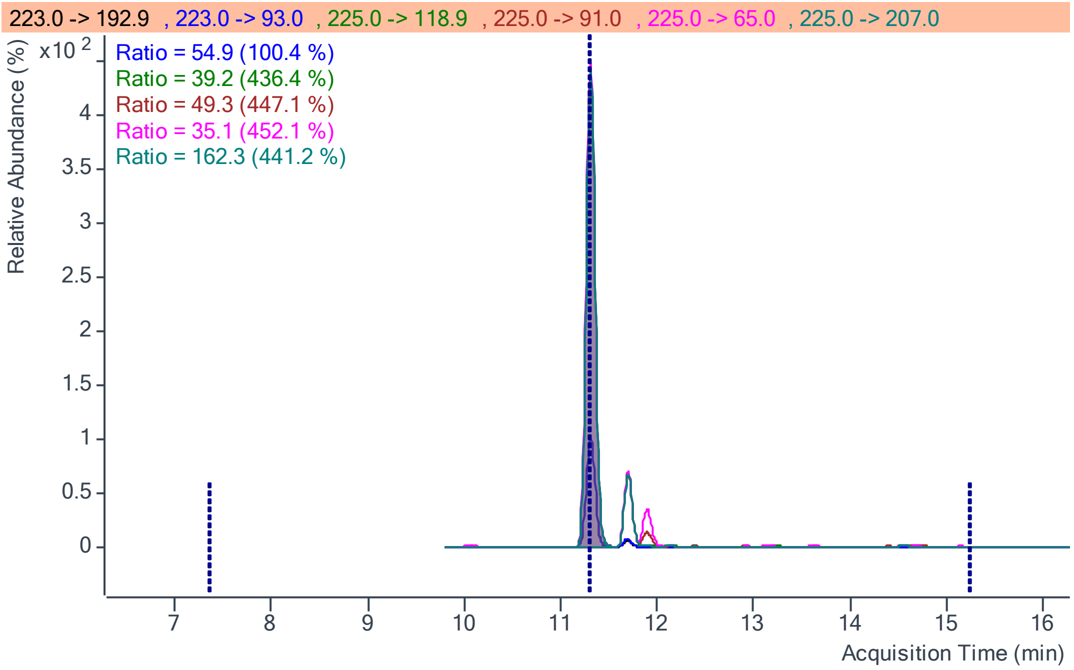
223.0 -> 192.9 , 223.0 -> 93.0 , 225.0 -> 118.9 , 225.0 -> 91.0 , 225.0 -> 65.0 , 225.0 -> 207.0
x10 2
Ratio = 54.9 (100.4 %)
Ratio = 39.2 (436.4 %)
Ratio = 49.3 (447.1 %)
Ratio = 35.1 (452.1 %)
Ratio = 162.3 (441.2 %)
Relative Abundance (%)
Acquisition Time (min)

(i)

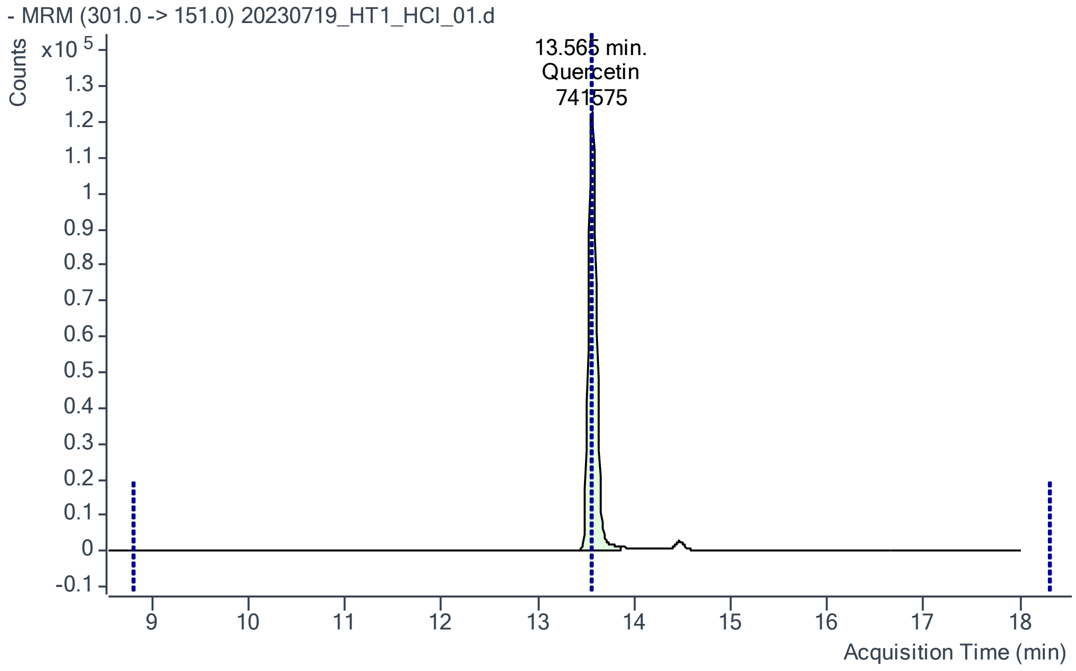
- MRM (301.0 -> 151.0) 20230719_HT1_HCl_01.d
Counts
x10 5
13.565 min.
Quercetin
741575
Acquisition Time (min)

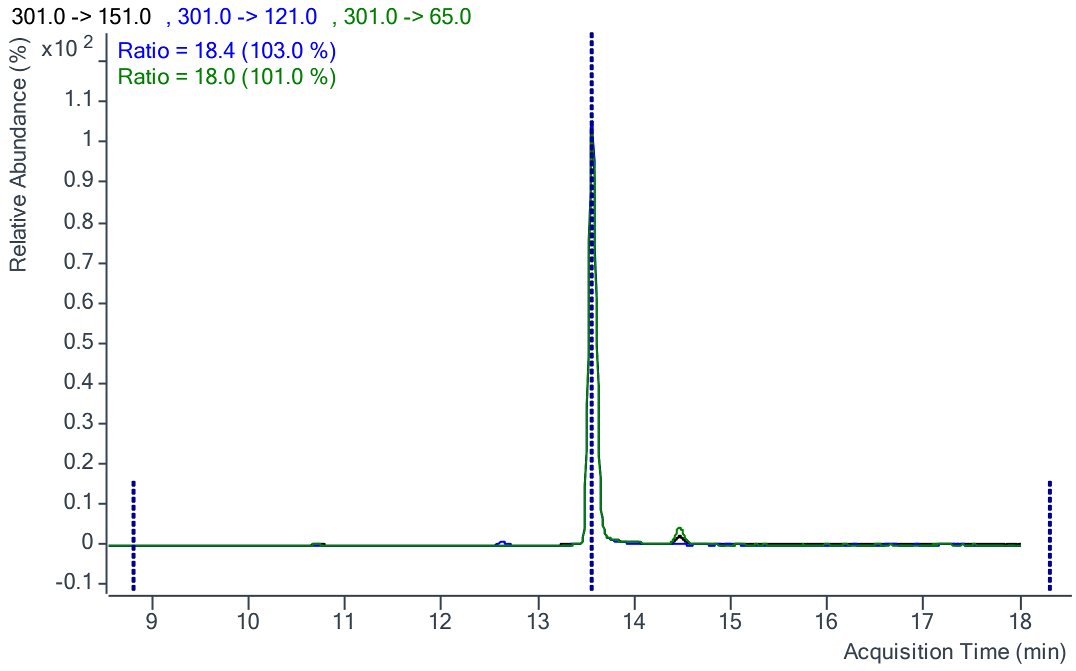
301.0 -> 151.0 , 301.0 -> 121.0 , 301.0 -> 65.0
Relative Abundance (%)
x10 2
Ratio = 18.4 (103.0 %)
Ratio = 18.0 (101.0 %)
Acquisition Time (min)

(j)

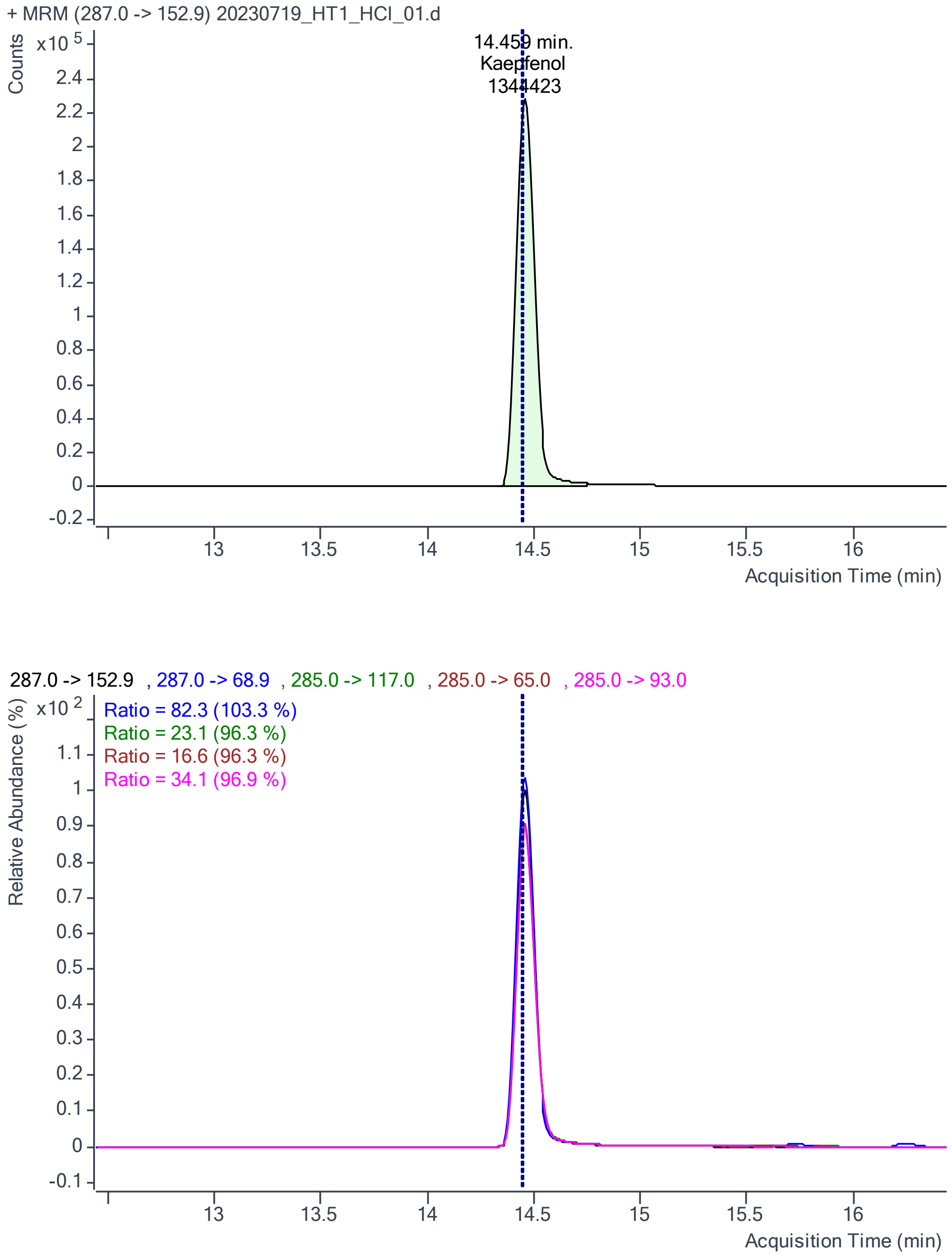

Figure S4. LC-MS/MS analysis of broccoli grown under high temperature. Nonhydrolyzed sample containing (a) *L*-ascorbic acid, (b) ferulic acid, (c) sinapic acid, (d) quercetin and (e) kaempferol. Hydrolyzed sample containing (f) *L*-ascorbic acid, (g) ferulic acid, (h) sinapic acid, (i) quercetin and (j) kaempferol.
