# Supplementary material for: Adjustments of the Phytochemical Profile of Broccoli to Low and High Growing Temperatures: Implications for the Bioactivity of Its Extracts
Source: Int J Mol Sci. 2024 Mar 26;25(7):3677. doi: 10.3390/ijms25073677 (PMC11011926; doi:10.3390/ijms25073677)
Supplement: Supplementary file 1 [file ijms-25-03677-s001.zip › Figure S5.pdf]

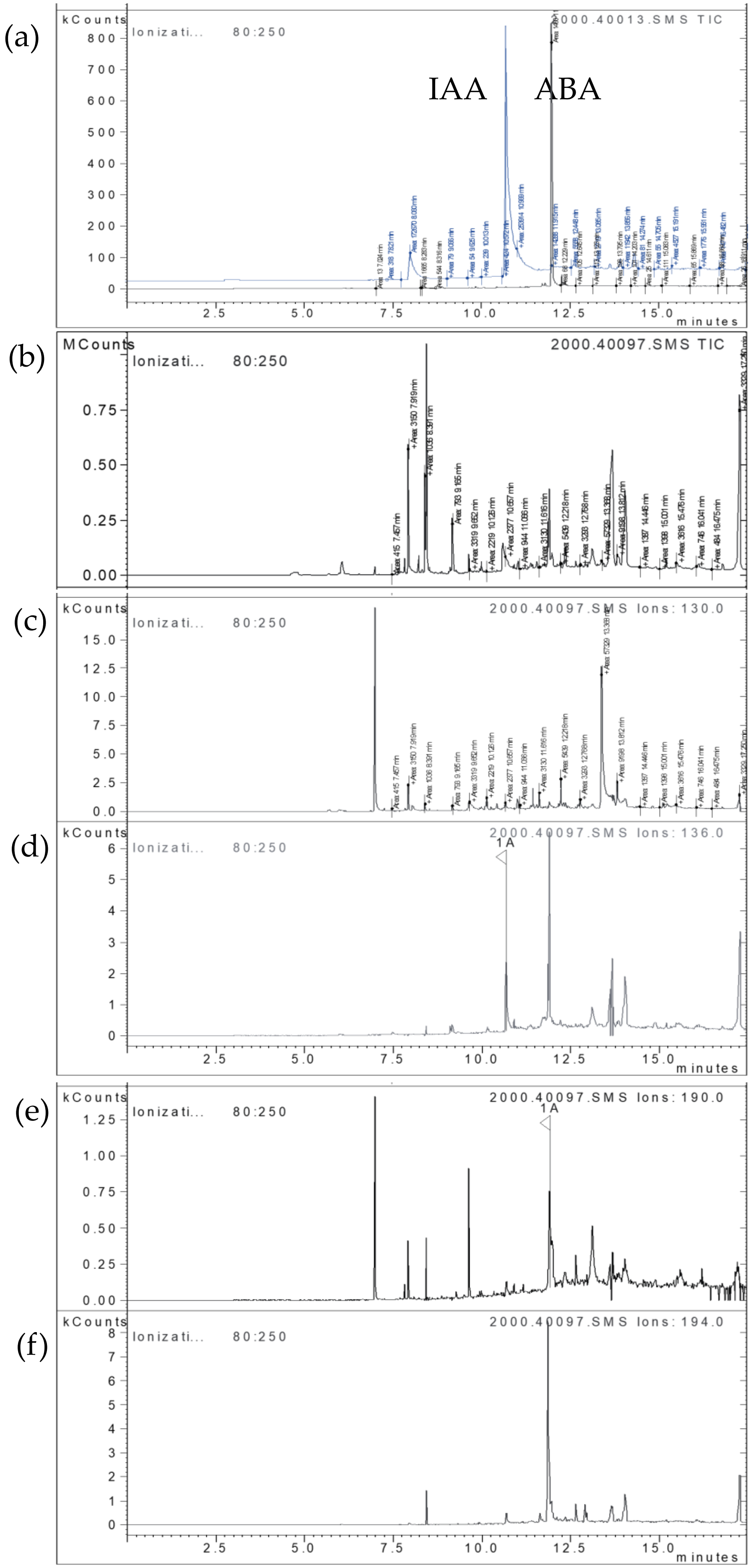
(a)
kCounts
Ionizati... 80:250
2000.40013.SMS TIC
IAA
ABA
(b)
MCounts
Ionizati... 80:250
2000.40097.SMS TIC
(c)
kCounts
Ionizati... 80:250
2000.40097.SMS Ions: 130.0
(d)
kCounts
Ionizati... 80:250
2000.40097.SMS Ions: 136.0
1A
(e)
kCounts
Ionizati... 80:250
2000.40097.SMS Ions: 190.0
1A
(f)
kCounts
Ionizati... 80:250
2000.40097.SMS Ions: 194.0
minutes

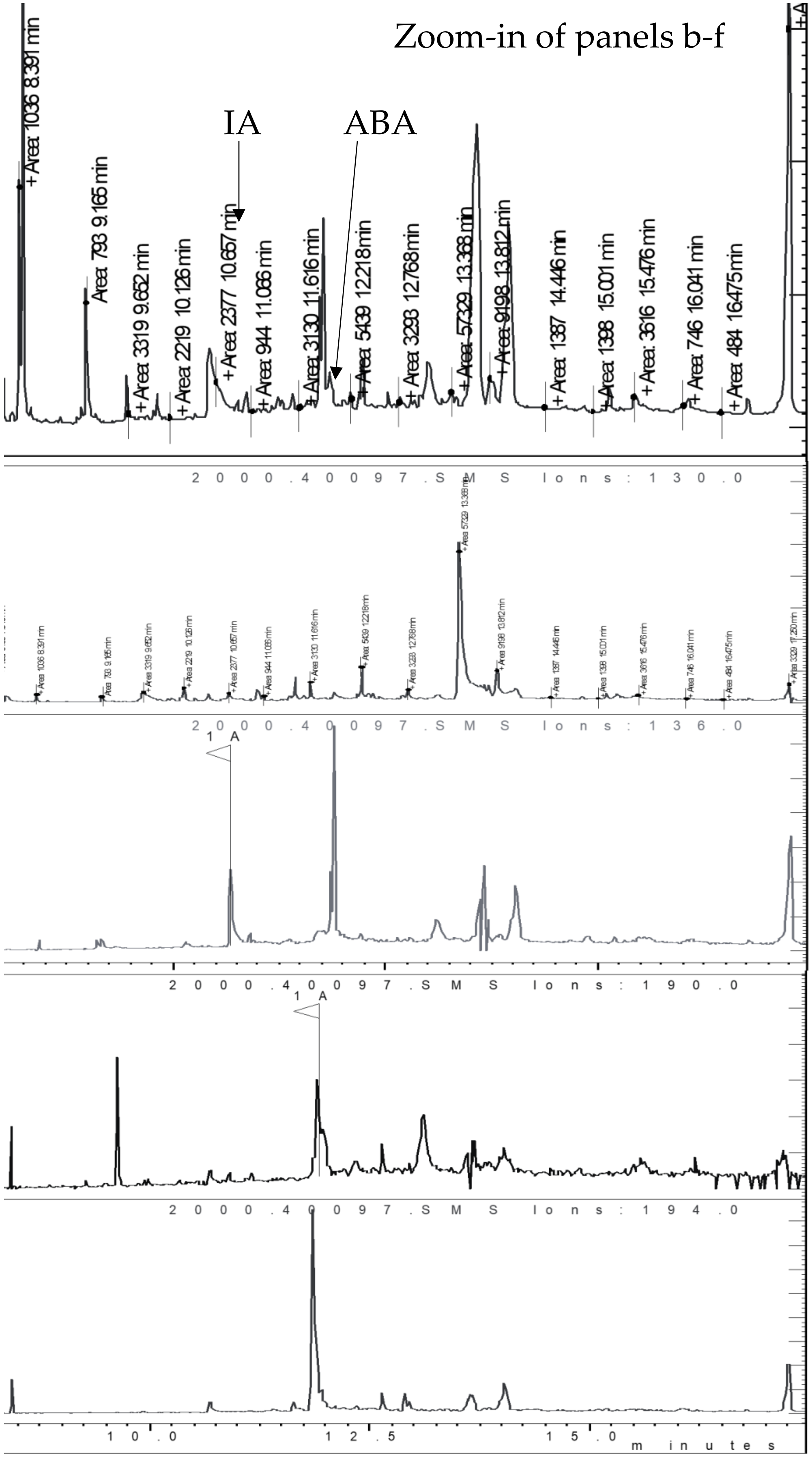

Zoom-in of panels b-f
IA
ABA
+ Area: 1036 8.391 min
Area: 793 9.165 min
+ Area: 3319 9.652 min
+ Area: 2219 10.126 min
+ Area: 2377 10.657 min
+ Area: 944 11.066 min
+ Area: 3130 11.616 min
+ Area: 5439 12.218 min
+ Area: 3293 12.768 min
+ Area: 57329 13.368 min
+ Area: 9198 13.812 min
+ Area: 1387 14.446 min
+ Area: 1398 15.001 min
+ Area: 3616 15.476 min
+ Area: 746 16.041 min
+ Area: 484 16.475 min
2000.40097.SMS Ions: 130.0
2000.40097.SMS Ions: 136.0
1 A
2000.40097.SMS Ions: 190.0
1 A
2000.40097.SMS Ions: 194.0
10.0
12.5
15.0
minutes

Figure S5. GC-MS chromatograms of standards and a selected sample. Upper panels: (a) standard chromatograms of IAA (blue line) and ABA (black line), (b) total ion chromatogram (TIC) of sample, (c) ion trace m/z 130 depicting endogenous IAA, (d) ion trace m/z 136 representing the labeled standard, (e) ion trace m/z 190 depicting endogenous ABA, (f) ion trace m/z 194 representing the labeled standard. Lower panels represent zoomed-in areas of the chromatograms in b-f. IAA and ABA peaks are indicated by arrows.
