# Supplementary material for: Adjustments of the Phytochemical Profile of Broccoli to Low and High Growing Temperatures: Implications for the Bioactivity of Its Extracts
Source: Int J Mol Sci. 2024 Mar 26;25(7):3677. doi: 10.3390/ijms25073677 (PMC11011926; doi:10.3390/ijms25073677)
Supplement: Supplementary file 1 [file ijms-25-03677-s001.zip › Table S7.pdf]

Supplementary Table S7. Retention times of compounds, ESI polarity, precursor ions, product ions and collision energy.

Compound Name	Retention time (min)	ESI polarity	Precursor Ion (m/z)	Product Ion (m/z)	Collision Energy (V)
L-ascorbic acid	2.79	negative	174.99	**115.0**	10
				87.0	18
Ferulic acid	11.68	negative	192.99	**134.0**	14
				149.0	10
				177.9	10
Sinapic acid	11.61	negative	222.99	**192.9**	22
				93.0	38
		positive	225.01	**207.0**	6
				65.0	54
				91.0	34
				118.9	22
Kaempferol	14.53	negative	284.99	**93.0**	38
				65.0	70
				117.0	50
		positive	287.01	**152.9**	38
				68.9	58
Quercetin	13.71	negative	300.99	**151.0**	22
				65.0	50
				121.0	30
